# Supplementary material for: Heterogeneity in the association between youth unemployment and mental health later in life: a quantile regression analysis of longitudinal data from English schoolchildren
Source: BMJ Open. 2021 Jul 19;11(7):e047997. doi: 10.1136/bmjopen-2020-047997 (PMC8291333; doi:10.1136/bmjopen-2020-047997)
Supplement: Supplementary data [file bmjopen-2020-047997supp001.pdf]

Supplementary Information

Methods

Locus of Control

Locus of control (LOC) was measured at age 14/15 with participants asked for their level of agreement (strongly agree, agree, disagree, strongly disagree, don't know) with six separate statements. Three of the statements were worded to reflect an internal LOC ("if someone is not a success in life, it is usually their own fault"; "I can pretty much decide what will happen in my life"; "if you work hard at something you'll usually succeed") and three worded to reflect an external LOC ("even if I do well at school, I'll have a hard time getting the right type of job", "people like me don't have much of a chance in life", "how well you get on in this world is mostly a matter of luck"). I place responses onto a five-point scale, centred around "don't know", with external-worded items reverse coded so higher scores indicate less external LOC.

There is no agreed way of combining items to operationalise LOC, both in the Next Steps data specifically and in other large-scale survey datasets which measure LOC (Buddelmeyer & Powdthavee, 2016; Caliendo et al., 2015; Cobb-Clark et al., 2014; Piatek & Pinger, 2016). Researchers using Next Steps data have used different subsets of the LOC items (cf. Crawford et al., 2011; Mendolia & Walker, 2015; Ng-Knight & Schoon, 2017; Wijedasa, 2017),[1] and combined them in several ways, including summing Likert responses (Crawford et al., 2011; Ng-Knight & Schoon, 2017) or extracting latent LOC scores using principal component analysis (Wijedasa, 2017), EFA (Mendolia & Walker, 2015), or CFA (Gladwell et al., 2016).

Though internal and external-worded items were originally conceptualized as tapping opposite ends of a single spectrum (Rotter, 1966), a number of exploratory factor analyses have found internal and external worded items load onto separate factors (Caliendo et al., 2015; Cobb-Clark & Schurer, 2013; Piatek & Pinger, 2016), including an analysis of Next Steps (Mendolia & Walker, 2015). In the Next Steps data, a CFA with two correlated latent factors has superior fit statistics to a single factor CFA model (Table 1). The correlation between the latent factors is just 0.4.

Table 1: Fit Statistics from Confirmatory Factor Analyses of Locus of Control Items

Model	RMSEA (95% CI)	CFI
One Factor	0.073 (0.069, 0.078)	0.818
Two Factor	0.044 (0.039, 0.05)	0.941
Hankins (2008)	0.034 (0.028, 0.04)	0.975

However, an alternative explanation is that responses reflect method effects. A single factor CFA with internal-worded items allowed to covary (i.e akin to Hankins' (2008) GHQ model) has superior fit statistics to the two-factor solution (Table 1). Given the possibility of method effects, the widespread use of only the first factor from EFA (Cobb-Clark, 2015), and the appeal of understanding locus of

[1] Wijedasa (2017) includes another item, "[w]orking hard at school now will help me get on later in life", their LOC measure, while Crawford et al. (2011) include this plus another item, "[d]oing well at school means a lot to me". I do not include these items as they arguably capture opinions about the value of secondary education rather than LOC. Ng-Knight & Schoon (2017) and Gladwell et al. (2016) use the three internal-worded items introduced above, only.

control as a singular construct (Rotter, 1975), we measure LOC by extracting a single factor using the Hankins (2008) CFA model (DWLS estimator).[2]

Note, the items are not drawn from a validated measure of LOC and have poor reliability (Cronbach's $\alpha = 0.40$). However, the items have been used in published studies before (Chowdry et al., 2011; Crawford et al., 2011; Department for Education et al., 2012; Gladwell et al., 2016; Mendolia & Walker, 2014a, 2014b, 2015; Ng-Knight & Schoon, 2017; Wijedasa, 2017). For instance, Mendolia & Walker (2015) find that participants with external LOC are more likely to be NEET by age 20, while Ng-Knight and Schoon (2017) find evidence that internal LOC moderates the association between low SEP and youth worklessness. The LOC factor we extract is also associated with several variables it should be expected to, including GHQ scores at age 25 and youth unemployment experience.

[2] An alternate model which allows covariance among external-worded items instead has poorer fit statistics.

Figures

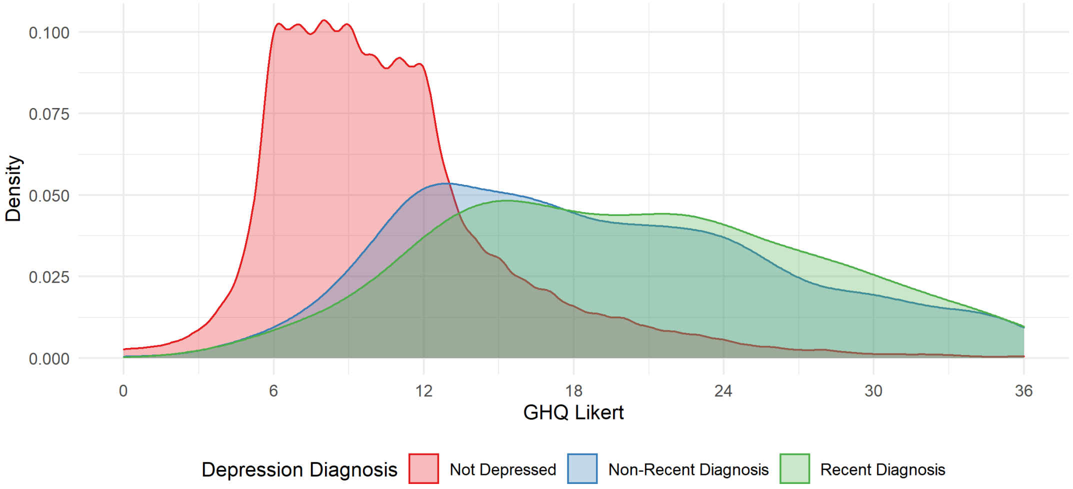

Figure S1: GHQ Likert Scores by recency of depression diagnosis. Recent diagnosis refers to diagnosis in prior two years. Non-recent diagnosis refers to diagnosis more than two years ago. Data drawn from Wave 1 of the United Kingdom Household Longitudinal Study, a representative panel survey of households in the UK.

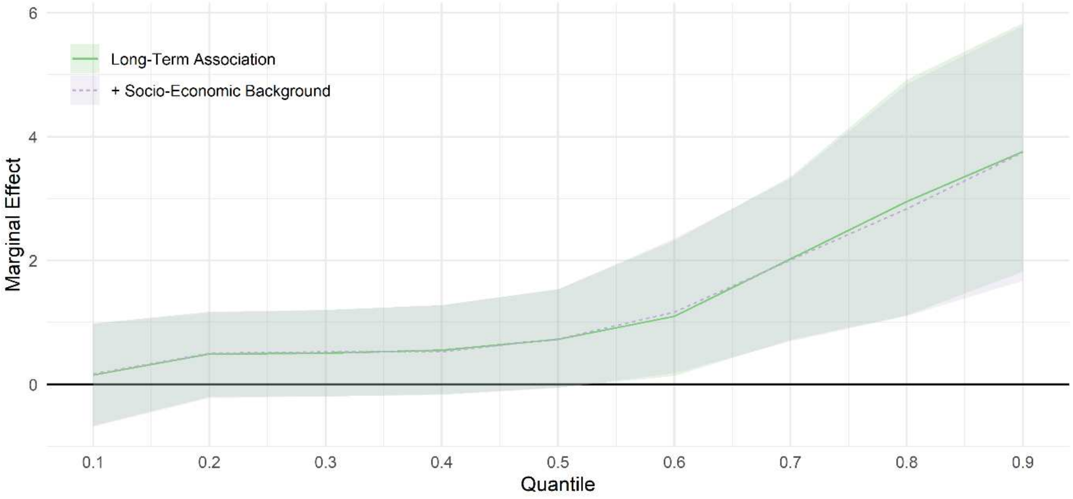

Figure S2: Association between 6+ months youth unemployment between ages 18-20 and GHQ-12 Likert scores at age 25, by decile of GHQ-12. (a) adjusted for adolescent mental health, disability and self-rated health, educational attainment, risk behaviours, attitude to school and bullying victimisation, IMD quintile, parental socio-economic class and education, gender, locus of control, and ethnicity. (b) additionally adjusted for family financial difficulties, number of household children, and household type.

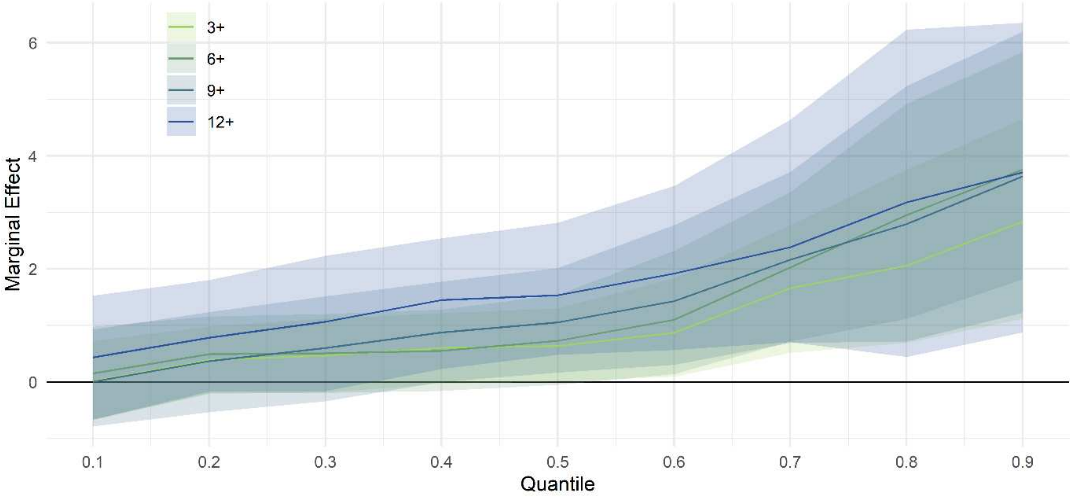

Figure S3: Association between youth unemployment between ages 18-20 and GHQ-12 Likert scores at age 25, by decile of GHQ-12 and cut-off used to define youth unemployment. Models adjusted for adolescent mental health, disability and self-rated health, educational attainment, risk behaviours, attitude to school and bullying victimisation, IMD quintile, parental socio-economic class and education, gender, locus of control, and ethnicity.

Tables

Table S1: Quantile Regression Results. Bivariate association (Column A, Table 2 in main paper)

Variable	Q10	Q20	Q30	Q40	Q50	Q60	Q70	Q80	Q90
6+ Months Unemployment	0<br>(-1, 1)	0<br>(0, 1)	1<br>(0, 2)	1<br>(0, 2)	1<br>(0, 2)	2<br>(0, 2)	3<br>(2, 4)	4<br>(3, 6)	5<br>(3, 8)
Constant	6<br>(5, 6)	7<br>(7, 7)	8<br>(8, 8)	9<br>(9, 10)	11<br>(10, 11)	12<br>(12, 12)	13<br>(13, 13)	16<br>(15, 16)	20<br>(20, 21)
Observations	7,363	7,363	7,363	7,363	7,363	7,363	7,363	7,363	7,363
Imputations	28	28	28	28	28	28	28	28	28

Table S2: Quantile Regression Results. Adjustment for adolescent mental health (Column B, Table 2 in main paper)

Variable	Q10	Q20	Q30	Q40	Q50	Q60	Q70	Q80	Q90
6+ Months Unemployment	0<br>(-0.79, 0.57)	0.36<br>(-0.31, 0.93)	0.48<br>(-0.15, 1.2)	0.67<br>(-0.07, 1.57)	1.1<br>(0.35, 1.79)	1.57<br>(0.67, 2.48)	2.41<br>(1.27, 3.85)	4<br>(1.95, 6.2)	6.2<br>(3.22, 7.62)
GHQ-12 @ Age 14/15	0.12<br>(0.02, 0.21)	0.14<br>(0.08, 0.22)	0.16<br>(0.06, 0.24)	0.18<br>(0.1, 0.27)	0.25<br>(0.17, 0.34)	0.33<br>(0.22, 0.43)	0.42<br>(0.28, 0.57)	0.56<br>(0.37, 0.8)	0.67<br>(0.49, 0.9)
GHQ-12 @ Age 16/17	0.14<br>(0.1, 0.18)	0.19<br>(0.15, 0.22)	0.21<br>(0.18, 0.25)	0.23<br>(0.2, 0.27)	0.25<br>(0.2, 0.29)	0.29<br>(0.24, 0.33)	0.33<br>(0.26, 0.38)	0.39<br>(0.32, 0.47)	0.43<br>(0.33, 0.54)
Constant	4.08<br>(3.6, 4.47)	5.01<br>(4.72, 5.36)	5.86<br>(5.55, 6.2)	6.83<br>(6.48, 7.2)	7.75<br>(7.29, 8.2)	8.43<br>(8, 8.93)	9.3<br>(8.79, 9.75)	10.5<br>(9.89, 11.09)	13.56<br>(12.76, 14.67)
Observations	7,363	7,363	7,363	7,363	7,363	7,363	7,363	7,363	7,363
Imputations	28	28	28	28	28	28	28	28	28

Table S3: Quantile Regression Results. Fully adjusted model excluding current economic activity (Column C, Table 2 in main paper)

	Variable	Q10	Q20	Q30	Q40	Q50	Q60	Q70	Q80	Q90
	6+ Months Unemployment	0.15 (-0.67, 0.98)	0.49 (-0.2, 1.16)	0.51 (-0.19, 1.2)	0.55 (-0.16, 1.28)	0.73 (-0.05, 1.54)	1.1 (0.14, 2.32)	2.03 (0.72, 3.36)	2.95 (1.12, 4.92)	3.76 (1.82, 5.83)
	Female	0.52 (0.17, 0.88)	0.58 (0.26, 0.89)	0.48 (0.16, 0.81)	0.35 (0.01, 0.71)	0.3 (-0.05, 0.65)	0.3 (-0.09, 0.68)	0.21 (-0.27, 0.68)	0 (-0.67, 0.64)	-0.68 (-1.59, 0.22)
	IMD	0 (-0.02, 0.01)	0 (-0.01, 0.01)	0 (-0.01, 0.01)	0 (-0.01, 0.01)	0 (-0.01, 0.02)	0 (-0.01, 0.02)	0.01 (-0.01, 0.02)	0.01 (-0.01, 0.04)	0.03 (-0.01, 0.06)
	Locus of Control	-0.2 (-0.39, -0.01)	-0.11 (-0.32, 0.09)	-0.13 (-0.34, 0.08)	-0.16 (-0.37, 0.06)	-0.12 (-0.35, 0.11)	-0.13 (-0.37, 0.12)	-0.02 (-0.33, 0.28)	0.03 (-0.38, 0.43)	0.02 (-0.54, 0.56)
	GHQ-12 @ Age 14/15	0.08 (-0.02, 0.17)	0.12 (0.04, 0.19)	0.09 (0.01, 0.18)	0.1 (0.01, 0.19)	0.17 (0.07, 0.27)	0.24 (0.14, 0.35)	0.29 (0.16, 0.44)	0.37 (0.18, 0.59)	0.48 (0.24, 0.72)
	GHQ-12 @ Age 16/17	0.13 (0.09, 0.17)	0.17 (0.13, 0.2)	0.2 (0.16, 0.23)	0.21 (0.17, 0.24)	0.23 (0.19, 0.27)	0.27 (0.22, 0.32)	0.31 (0.25, 0.37)	0.35 (0.27, 0.43)	0.4 (0.31, 0.49)
Self-Rated Health @ Age 14/15	Fairly Good	0.13 (-0.27, 0.55)	0.23 (-0.09, 0.55)	0.34 (-0.02, 0.69)	0.39 (0.03, 0.75)	0.37 (-0.02, 0.74)	0.31 (-0.1, 0.71)	0.3 (-0.21, 0.79)	0.51 (-0.15, 1.17)	0.97 (0, 1.98)
	Not Very Good	0.31 (-1.01, 1.58)	0.38 (-0.83, 1.56)	0.42 (-0.82, 2.06)	0.99 (-0.81, 2.23)	0.85 (-0.48, 2.06)	0.67 (-0.73, 2.65)	1.13 (-0.84, 3.71)	1.64 (-0.95, 4.78)	1.7 (-1.14, 5.03)
	Not Good at All	0.12 (-2.52, 1.88)	-0.34 (-1.67, 1.81)	-0.63 (-2.07, 2.18)	-0.31 (-2.67, 2.97)	-0.06 (-3.17, 3.68)	0.08 (-3.01, 4.34)	0.26 (-3.4, 5.97)	-0.01 (-3.86, 7.18)	1.42 (-4.87, 5.87)
Self-Rated Health @ Age 16/17	Fairly Good	0.26 (-0.14, 0.68)	0.18 (-0.16, 0.52)	0.15 (-0.21, 0.52)	0.22 (-0.15, 0.61)	0.17 (-0.23, 0.57)	0.1 (-0.31, 0.54)	0.19 (-0.35, 0.72)	0.24 (-0.47, 0.97)	0.14 (-0.87, 1.22)

	Variable	Q10	Q20	Q30	Q40	Q50	Q60	Q70	Q80	Q90
	Not Very Good	-0.03 (-0.9, 0.75)	-0.09 (-1.1, 0.87)	0.31 (-0.65, 1.32)	0.61 (-0.28, 1.46)	0.59 (-0.25, 1.44)	0.52 (-0.43, 1.85)	0.7 (-0.56, 2.23)	0.61 (-1.01, 2.34)	0.12 (-1.9, 2.15)
	Not Good at All	-0.88 (-2.98, 1.87)	0.15 (-2.45, 1.91)	0.1 (-1.93, 1.93)	0.23 (-1.83, 2.22)	0.69 (-1.93, 2.64)	0.39 (-1.82, 4.8)	1.47 (-1.8, 5.56)	1.59 (-2.31, 5.5)	-0.16 (-3.49, 4.02)
Disabled	Yes, school not affected	0.12 (-0.57, 0.69)	0.08 (-0.42, 0.56)	-0.11 (-0.61, 0.41)	-0.14 (-0.75, 0.55)	0.06 (-0.59, 0.7)	0.24 (-0.51, 1.16)	0.65 (-0.28, 1.51)	0.79 (-0.38, 2.25)	1.49 (-0.39, 3.44)
	Yes, school affected	0.39 (-0.67, 1.32)	0.4 (-0.42, 1.15)	0.33 (-0.4, 1.04)	0.31 (-0.51, 1.23)	0.57 (-0.26, 1.34)	0.6 (-0.29, 1.55)	0.76 (-0.48, 2.51)	1.4 (-0.38, 3.66)	2.24 (-0.18, 4.91)
	Risk Behaviours	-0.03 (-0.18, 0.08)	-0.1 (-0.2, 0.03)	-0.11 (-0.25, 0.06)	-0.07 (-0.23, 0.08)	-0.04 (-0.18, 0.13)	-0.01 (-0.19, 0.18)	0.01 (-0.2, 0.23)	0.01 (-0.29, 0.38)	0.03 (-0.37, 0.41)
	Attitude to School	-0.02 (-0.05, 0.01)	-0.01 (-0.04, 0.02)	-0.02 (-0.05, 0.01)	-0.02 (-0.05, 0.01)	-0.02 (-0.05, 0.01)	-0.02 (-0.05, 0.01)	-0.03 (-0.07, 0.01)	-0.05 (-0.11, 0.01)	-0.06 (-0.13, 0.02)
	# Waves Bullied, 1-3	0.07 (-0.09, 0.24)	0.11 (-0.02, 0.25)	0.17 (0.02, 0.33)	0.23 (0.08, 0.39)	0.27 (0.11, 0.43)	0.33 (0.15, 0.5)	0.39 (0.16, 0.62)	0.55 (0.21, 0.9)	0.7 (0.26, 1.17)
Qualifications	NVQ 4	0.52 (-0.02, 1.08)	0.38 (-0.04, 0.82)	0.48 (0, 0.94)	0.31 (-0.18, 0.82)	0.28 (-0.22, 0.77)	0.35 (-0.19, 0.88)	0.41 (-0.23, 1.06)	0.36 (-0.62, 1.25)	0.27 (-1.23, 1.68)
	NVQ 3	0.55 (-0.05, 1.14)	0.38 (-0.03, 0.82)	0.38 (-0.1, 0.86)	0.32 (-0.23, 0.87)	0.38 (-0.16, 0.9)	0.42 (-0.16, 0.99)	0.46 (-0.21, 1.1)	0.32 (-0.7, 1.32)	0.21 (-1.29, 1.66)
	NVQ 2	0.26 (-0.33, 0.86)	0.26 (-0.19, 0.72)	0.26 (-0.24, 0.77)	0.09 (-0.45, 0.64)	-0.02 (-0.55, 0.5)	-0.06 (-0.67, 0.54)	-0.09 (-0.82, 0.65)	-0.56 (-1.65, 0.49)	-1.33 (-2.89, 0.12)

	Variable	Q10	Q20	Q30	Q40	Q50	Q60	Q70	Q80	Q90
	NVQ 1	-0.48 (-1.42, 0.35)	-0.41 (-1.12, 0.3)	-0.28 (-1.04, 0.46)	-0.32 (-1.1, 0.49)	-0.13 (-0.9, 0.68)	0.13 (-0.73, 0.98)	0.23 (-0.77, 1.46)	0.73 (-0.89, 2.35)	0.69 (-1.45, 3.03)
	No/Other Qual	-0.39 (-1.37, 0.58)	-0.19 (-1.04, 0.59)	-0.11 (-0.93, 0.73)	-0.16 (-1.05, 0.76)	-0.04 (-0.97, 0.91)	0.29 (-0.75, 1.37)	0.74 (-0.56, 2.36)	1.17 (-0.78, 3.17)	0.15 (-2.06, 2.48)
Parental NS-SEC	Intermediate	0.14 (-0.39, 0.64)	0.09 (-0.32, 0.49)	0 (-0.4, 0.41)	-0.08 (-0.52, 0.38)	-0.15 (-0.63, 0.39)	-0.15 (-0.69, 0.4)	-0.08 (-0.7, 0.59)	0.23 (-0.64, 1.16)	0.43 (-0.83, 1.77)
	Routine	0.3 (-0.15, 0.75)	0.24 (-0.15, 0.64)	0.34 (-0.1, 0.75)	0.38 (-0.09, 0.84)	0.3 (-0.2, 0.79)	0.22 (-0.31, 0.78)	0.35 (-0.27, 0.96)	0.69 (-0.12, 1.49)	0.77 (-0.52, 2)
	LTU	-0.19 (-1.51, 0.97)	-0.25 (-1.31, 0.74)	-0.2 (-1.22, 0.9)	-0.05 (-1.2, 1.2)	0 (-1.24, 1.28)	0.32 (-1.15, 1.95)	1.03 (-0.64, 2.78)	1.44 (-0.62, 3.75)	1.98 (-0.85, 4.88)
Parental Education	Other HE	-0.24 (-0.8, 0.32)	-0.11 (-0.57, 0.35)	-0.21 (-0.75, 0.36)	-0.01 (-0.58, 0.54)	0.05 (-0.55, 0.63)	0.09 (-0.54, 0.69)	0.13 (-0.64, 0.89)	0.07 (-0.87, 1.07)	0.33 (-0.97, 1.74)
	A-Level	-0.24 (-0.84, 0.33)	-0.19 (-0.68, 0.31)	-0.37 (-0.88, 0.2)	-0.27 (-0.83, 0.3)	-0.19 (-0.83, 0.45)	-0.11 (-0.78, 0.52)	-0.29 (-1.04, 0.43)	-0.61 (-1.57, 0.42)	-0.36 (-1.83, 1.21)
	GCSE A-C	-0.08 (-0.65, 0.49)	0 (-0.48, 0.49)	-0.11 (-0.6, 0.4)	-0.15 (-0.66, 0.35)	-0.16 (-0.79, 0.47)	-0.04 (-0.71, 0.59)	-0.1 (-0.82, 0.6)	-0.44 (-1.38, 0.54)	-0.5 (-1.95, 0.93)
	Other/None	-0.42 (-1.08, 0.28)	-0.03 (-0.66, 0.57)	-0.06 (-0.72, 0.57)	0.08 (-0.59, 0.81)	0.37 (-0.41, 1.16)	0.51 (-0.35, 1.34)	0.48 (-0.53, 1.5)	0.46 (-0.79, 1.75)	1.43 (-0.58, 3.39)
Ethnicity	Mixed	-0.69 (-1.77, 0.17)	-0.67 (-1.62, 0.16)	-0.6 (-1.56, 0.19)	-0.72 (-1.54, 0.02)	-0.98 (-1.75, -0.01)	-0.86 (-1.94, 0.5)	-0.23 (-1.75, 1.75)	0.47 (-1.27, 3.12)	1.42 (-1.57, 5.03)

Variable	Q10	Q20	Q30	Q40	Q50	Q60	Q70	Q80	Q90
Indian	-1.17 (-1.92, -0.27)	-0.81 (-1.43, -0.22)	-0.86 (-1.52, -0.07)	-0.62 (-1.44, 0.16)	-0.62 (-1.36, 0.13)	-0.48 (-1.35, 0.43)	-0.31 (-1.29, 0.96)	0.13 (-1.37, 1.66)	0.12 (-1.98, 3.03)
Pakistani	-0.65 (-1.49, 0.22)	-0.44 (-1.19, 0.22)	-0.61 (-1.27, 0.07)	-0.89 (-1.65, -0.14)	-1.21 (-1.99, -0.43)	-1.2 (-1.99, -0.29)	-1 (-2.13, 0.16)	-1.24 (-2.62, 0.16)	-1.9 (-3.98, 0.47)
Bangladeshi	-1.72 (-3.04, -0.33)	-1.18 (-2.63, 0.03)	-0.75 (-1.87, 0.13)	-0.86 (-1.84, 0.12)	-1.08 (-2.16, 0.15)	-1.05 (-2.26, 0.07)	-1.42 (-2.66, 0)	-1.95 (-3.68, -0.23)	-3.41 (-5.54, -0.95)
Black African	-0.45 (-1.54, 0.41)	-0.54 (-1.43, 0.46)	-0.44 (-1.54, 0.6)	-0.42 (-1.52, 0.69)	-0.6 (-1.66, 0.47)	-0.73 (-2, 0.73)	-0.69 (-2.04, 0.87)	-1.02 (-2.72, 0.86)	-1.8 (-3.95, 1.38)
Black Caribbean	-1.8 (-2.93, -0.58)	-1.22 (-2.08, -0.33)	-1.06 (-1.89, -0.22)	-1.03 (-1.95, 0.07)	-0.75 (-1.88, 0.17)	-0.76 (-1.68, 0.34)	-0.66 (-1.87, 0.82)	-0.72 (-2.34, 1.28)	-1.28 (-3.57, 1.99)
Other	-0.82 (-3.1, 0.55)	-0.32 (-1.54, 0.75)	-0.18 (-1.23, 1.16)	0.27 (-1.19, 1.63)	0.47 (-0.9, 2.13)	1.02 (-0.72, 2.46)	1.11 (-0.38, 2.64)	0.86 (-0.82, 3.36)	-0.07 (-2.45, 2.77)
Constant	4.37 (2.88, 5.65)	5.07 (3.85, 6.31)	6.28 (5.02, 7.4)	7.06 (5.87, 8.29)	7.72 (6.36, 9.07)	8.02 (6.61, 9.42)	9.03 (7.29, 10.89)	10.91 (8.12, 13.58)	13.59 (10.19, 16.82)
Observations	7,363	7,363	7,363	7,363	7,363	7,363	7,363	7,363	7,363
Imputations	28	28	28	28	28	28	28	28	28

Table S4: Quantile Regression Results. Fully adjusted model including current economic activity (Column D, Table 2 in main paper)

	Variable	Q10	Q20	Q30	Q40	Q50	Q60	Q70	Q80	Q90
	6+ Months Unemployment	0.1 (-0.78, 0.95)	0.42 (-0.3, 1.03)	0.28 (-0.4, 0.94)	0.22 (-0.46, 0.92)	0.22 (-0.59, 1.09)	0.52 (-0.4, 1.73)	1.55 (0.07, 2.82)	2.24 (0.73, 4.02)	2.69 (0.92, 4.42)
	Female	0.45 (0.1, 0.81)	0.54 (0.23, 0.84)	0.46 (0.14, 0.78)	0.31 (-0.03, 0.65)	0.21 (-0.16, 0.57)	0.2 (-0.19, 0.58)	0.11 (-0.37, 0.56)	-0.18 (-0.79, 0.41)	-0.76 (-1.64, 0.11)
	IMD	0 (-0.02, 0.01)	0 (-0.01, 0.01)	0 (-0.01, 0.01)	0 (-0.02, 0.01)	0 (-0.01, 0.01)	0 (-0.01, 0.02)	0 (-0.02, 0.02)	0 (-0.02, 0.02)	0.02 (-0.01, 0.05)
	Locus of Control	-0.19 (-0.38, 0.01)	-0.09 (-0.29, 0.1)	-0.06 (-0.27, 0.16)	-0.08 (-0.29, 0.15)	-0.05 (-0.28, 0.18)	-0.02 (-0.26, 0.2)	0.05 (-0.25, 0.35)	0.14 (-0.27, 0.51)	-0.02 (-0.54, 0.5)
Current Economic Activity	Education	0.67 (-0.26, 1.47)	0.59 (-0.09, 1.26)	0.58 (-0.2, 1.39)	0.68 (-0.14, 1.58)	0.84 (-0.11, 1.78)	1.12 (0.16, 2.2)	1.6 (0.29, 3.03)	2.23 (0.41, 4.37)	2.39 (0.55, 4.47)
	Inactive	0.53 (-0.08, 1.28)	0.4 (-0.26, 1.02)	0.48 (-0.26, 1.23)	0.83 (-0.03, 1.7)	1.21 (0.28, 2.41)	1.95 (0.74, 3.2)	2.78 (1.33, 4.35)	3.74 (1.94, 5.96)	4.64 (2.58, 6.78)
	Unemployed	1.45 (0.34, 2.59)	2.16 (0.98, 3.03)	2.36 (1.53, 3.17)	2.52 (1.65, 3.54)	2.88 (1.67, 4.03)	3.28 (2.13, 4.48)	4.01 (2.51, 5.82)	4.71 (3.02, 6.28)	4.67 (2.84, 6.64)
	GHQ-12 @ Age 14/15	0.09 (-0.01, 0.18)	0.12 (0.04, 0.2)	0.1 (0.02, 0.18)	0.11 (0.02, 0.2)	0.17 (0.07, 0.27)	0.24 (0.13, 0.34)	0.28 (0.16, 0.43)	0.37 (0.2, 0.57)	0.51 (0.29, 0.75)
	GHQ-12 @ Age 16/17	0.13 (0.09, 0.17)	0.16 (0.12, 0.2)	0.19 (0.15, 0.23)	0.21 (0.17, 0.24)	0.23 (0.19, 0.27)	0.26 (0.22, 0.31)	0.29 (0.24, 0.35)	0.32 (0.25, 0.4)	0.37 (0.29, 0.46)
Self-Rated Health @ Age 14/15	Fairly Good	0.11 (-0.29, 0.52)	0.21 (-0.12, 0.53)	0.3 (-0.05, 0.65)	0.42 (0.07, 0.76)	0.46 (0.06, 0.85)	0.37 (-0.04, 0.77)	0.31 (-0.18, 0.79)	0.48 (-0.15, 1.12)	0.99 (0.05, 1.97)

	Variable	Q10	Q20	Q30	Q40	Q50	Q60	Q70	Q80	Q90
	Not Very Good	0.18 (-1.22, 1.5)	0.5 (-0.94, 1.68)	0.46 (-0.73, 1.95)	0.87 (-0.79, 2.16)	0.86 (-0.57, 2.02)	0.47 (-0.77, 2.23)	0.8 (-1.06, 3.7)	1.61 (-1.26, 5.06)	2.45 (-0.4, 5.22)
	Not Good at All	-0.27 (-2.68, 1.73)	-0.46 (-2.57, 1.71)	-0.73 (-2.33, 2.02)	-0.21 (-2.84, 2.66)	-0.24 (-3.1, 3)	-0.56 (-3.13, 3.44)	-0.98 (-3.85, 5.02)	-0.38 (-4.85, 5.76)	-0.11 (-5.23, 5.46)
Self-Rated Health @ Age 16/17	Fairly Good	0.24 (-0.18, 0.66)	0.21 (-0.14, 0.55)	0.13 (-0.23, 0.5)	0.2 (-0.18, 0.6)	0.2 (-0.21, 0.61)	0.08 (-0.33, 0.51)	0.01 (-0.5, 0.53)	0.24 (-0.44, 0.93)	0.19 (-0.8, 1.2)
	Not Very Good	-0.07 (-0.94, 0.82)	-0.03 (-1.06, 0.92)	0.33 (-0.66, 1.31)	0.55 (-0.34, 1.39)	0.52 (-0.37, 1.39)	0.41 (-0.55, 1.6)	0.57 (-0.71, 2.09)	0.69 (-1.01, 2.26)	-0.27 (-1.96, 1.53)
	Not Good at All	-0.93 (-2.87, 1.78)	-0.13 (-2.54, 1.99)	0.24 (-2.38, 2.07)	0.38 (-1.69, 2.36)	0.84 (-1.66, 2.7)	0.61 (-1.66, 4.31)	1.09 (-1.6, 5.38)	1.31 (-2.16, 4.9)	-1.04 (-4.08, 2.68)
Disabled	Yes, school not affected	0.15 (-0.51, 0.72)	0.06 (-0.45, 0.55)	-0.04 (-0.54, 0.48)	-0.11 (-0.69, 0.54)	0.1 (-0.61, 0.79)	0.21 (-0.47, 1.06)	0.63 (-0.34, 1.57)	0.63 (-0.46, 1.91)	1.23 (-0.5, 3.11)
	Yes, school affected	0.25 (-0.88, 1.19)	0.38 (-0.47, 1.13)	0.35 (-0.37, 1.02)	0.16 (-0.56, 0.97)	0.29 (-0.62, 1.2)	0.39 (-0.53, 1.34)	0.48 (-0.61, 1.95)	0.83 (-0.81, 2.66)	1.39 (-1.1, 4.42)
	Risk Behaviours	-0.06 (-0.21, 0.07)	-0.1 (-0.21, 0.03)	-0.1 (-0.24, 0.07)	-0.07 (-0.24, 0.08)	-0.06 (-0.22, 0.11)	-0.04 (-0.22, 0.15)	-0.02 (-0.22, 0.19)	-0.03 (-0.3, 0.29)	-0.09 (-0.43, 0.27)
	Attitude to School	-0.02 (-0.05, 0.01)	-0.01 (-0.04, 0.02)	-0.03 (-0.05, 0.01)	-0.02 (-0.05, 0.01)	-0.02 (-0.05, 0.01)	-0.03 (-0.06, 0.01)	-0.03 (-0.07, 0)	-0.05 (-0.11, 0.01)	-0.05 (-0.12, 0.03)
	# Waves Bullied, 1-3	0.08 (-0.09, 0.25)	0.1 (-0.04, 0.23)	0.16 (0.01, 0.31)	0.21 (0.06, 0.36)	0.23 (0.06, 0.4)	0.3 (0.12, 0.47)	0.34 (0.13, 0.56)	0.5 (0.17, 0.82)	0.63 (0.24, 1.04)

	Variable	Q10	Q20	Q30	Q40	Q50	Q60	Q70	Q80	Q90
Qualifications	NVQ 4	0.49 (-0.04, 1.03)	0.34 (-0.07, 0.79)	0.39 (-0.08, 0.86)	0.31 (-0.18, 0.81)	0.3 (-0.21, 0.8)	0.37 (-0.16, 0.9)	0.47 (-0.18, 1.11)	0.43 (-0.51, 1.31)	0.25 (-1.1, 1.57)
	NVQ 3	0.53 (-0.07, 1.12)	0.41 (-0.02, 0.86)	0.36 (-0.12, 0.85)	0.29 (-0.25, 0.84)	0.38 (-0.19, 0.94)	0.48 (-0.09, 1.05)	0.53 (-0.15, 1.19)	0.37 (-0.63, 1.37)	0.26 (-1.12, 1.56)
	NVQ 2	0.26 (-0.34, 0.89)	0.29 (-0.16, 0.75)	0.26 (-0.24, 0.76)	0.12 (-0.41, 0.67)	0.02 (-0.51, 0.56)	-0.08 (-0.67, 0.53)	-0.12 (-0.88, 0.58)	-0.62 (-1.63, 0.36)	-1.15 (-2.59, 0.19)
	NVQ 1	-0.58 (-1.54, 0.29)	-0.57 (-1.26, 0.19)	-0.35 (-1.05, 0.37)	-0.4 (-1.18, 0.39)	-0.26 (-1.05, 0.56)	0.02 (-0.85, 0.89)	0.08 (-0.95, 1.14)	0.28 (-1.31, 1.95)	0.19 (-1.81, 2.27)
	No/Other Qual	-0.48 (-1.46, 0.5)	-0.24 (-1.11, 0.58)	-0.24 (-1.05, 0.63)	-0.23 (-1.15, 0.67)	-0.14 (-1.09, 0.81)	0.03 (-0.98, 1.05)	0.29 (-1.02, 1.65)	0.08 (-1.64, 1.85)	-1.06 (-3.09, 1.16)
Parental NS-SEC	Intermediate	0.13 (-0.38, 0.66)	0.1 (-0.31, 0.48)	-0.03 (-0.43, 0.36)	-0.11 (-0.55, 0.35)	-0.17 (-0.69, 0.36)	-0.11 (-0.64, 0.44)	0.05 (-0.54, 0.67)	0.32 (-0.54, 1.23)	0.54 (-0.64, 1.83)
	Routine	0.27 (-0.2, 0.74)	0.25 (-0.15, 0.67)	0.29 (-0.13, 0.7)	0.32 (-0.13, 0.78)	0.26 (-0.24, 0.79)	0.17 (-0.35, 0.74)	0.38 (-0.23, 0.98)	0.66 (-0.15, 1.4)	0.41 (-0.77, 1.68)
	LTU	-0.12 (-1.46, 1.04)	-0.2 (-1.23, 0.76)	-0.14 (-1.13, 0.95)	0.08 (-1.09, 1.36)	0.17 (-1.11, 1.36)	0.09 (-1.13, 1.53)	0.72 (-0.96, 2.58)	1.3 (-0.75, 3.62)	1.76 (-0.83, 4.31)
Parental Education	Other HE	-0.2 (-0.76, 0.35)	-0.1 (-0.57, 0.37)	-0.17 (-0.71, 0.41)	0.02 (-0.54, 0.56)	0.08 (-0.5, 0.65)	0.14 (-0.5, 0.74)	0.18 (-0.56, 0.9)	0.21 (-0.73, 1.23)	0.76 (-0.52, 2.1)
	A-Level	-0.22 (-0.83, 0.35)	-0.17 (-0.66, 0.32)	-0.33 (-0.85, 0.22)	-0.26 (-0.81, 0.29)	-0.13 (-0.79, 0.5)	-0.09 (-0.75, 0.53)	-0.22 (-0.96, 0.49)	-0.36 (-1.31, 0.64)	0.09 (-1.32, 1.58)

	Variable	Q10	Q20	Q30	Q40	Q50	Q60	Q70	Q80	Q90
	GCSE A-C	-0.05 (-0.64, 0.55)	0.05 (-0.45, 0.54)	-0.07 (-0.57, 0.43)	-0.13 (-0.62, 0.37)	-0.11 (-0.74, 0.51)	0.01 (-0.64, 0.65)	0.02 (-0.68, 0.7)	-0.25 (-1.12, 0.69)	-0.01 (-1.42, 1.29)
	Other/None	-0.51 (-1.18, 0.2)	-0.12 (-0.72, 0.49)	-0.08 (-0.73, 0.54)	-0.01 (-0.65, 0.69)	0.28 (-0.54, 1.12)	0.45 (-0.4, 1.26)	0.29 (-0.66, 1.27)	0.48 (-0.79, 1.84)	1.67 (-0.16, 3.46)
Ethnicity	Mixed	-0.77 (-1.92, 0.18)	-0.64 (-1.57, 0.13)	-0.63 (-1.54, 0.17)	-0.73 (-1.59, -0.03)	-1 (-1.73, -0.09)	-0.93 (-1.99, 0.45)	-0.37 (-1.78, 1.59)	0.43 (-1.32, 3.03)	1.71 (-1.44, 5.01)
	Indian	-1.17 (-1.88, -0.38)	-0.85 (-1.6, -0.22)	-0.78 (-1.47, -0.05)	-0.64 (-1.42, 0.17)	-0.55 (-1.35, 0.22)	-0.42 (-1.22, 0.44)	-0.18 (-1.21, 1.03)	0.18 (-1.16, 1.37)	-0.77 (-2.28, 1.4)
	Pakistani	-0.59 (-1.5, 0.25)	-0.59 (-1.35, 0.07)	-0.71 (-1.43, -0.04)	-0.98 (-1.71, -0.2)	-1.26 (-2.06, -0.44)	-1.27 (-2.1, -0.35)	-1.03 (-2.19, 0.14)	-1.07 (-2.45, 0.29)	-1.98 (-3.85, 0.47)
	Bangladeshi	-1.71 (-3.03, -0.3)	-1.23 (-2.68, -0.07)	-0.79 (-1.95, 0.13)	-0.84 (-1.85, 0.14)	-1.07 (-2.15, 0.2)	-0.92 (-2.14, 0.25)	-1.02 (-2.31, 0.37)	-1.44 (-3.16, 0.2)	-2.82 (-4.84, -0.58)
	Black African	-0.47 (-1.51, 0.44)	-0.52 (-1.38, 0.35)	-0.52 (-1.48, 0.46)	-0.62 (-1.65, 0.34)	-0.94 (-1.98, 0.34)	-0.76 (-2.16, 0.79)	-0.56 (-1.99, 0.99)	-0.64 (-2.29, 1.28)	-1.28 (-3.4, 1.69)
	Black Caribbean	-1.77 (-2.87, -0.63)	-1.28 (-2.17, -0.42)	-1.16 (-1.99, -0.36)	-1.22 (-2.08, -0.14)	-0.99 (-2.2, 0.06)	-0.82 (-1.83, 0.2)	-0.75 (-1.89, 0.67)	-0.85 (-2.3, 1.2)	-1.46 (-3.66, 1.08)
	Other	-0.92 (-3.08, 0.41)	-0.45 (-1.8, 0.7)	-0.21 (-1.34, 0.89)	-0.07 (-1.33, 1.43)	0.34 (-1.22, 1.88)	0.71 (-0.94, 2.18)	0.91 (-0.73, 2.57)	0.72 (-1.11, 3.13)	0.4 (-2.41, 3.51)
	Constant	4.34 (2.8, 5.66)	4.94 (3.73, 6.2)	6.36 (5.07, 7.49)	7.06 (5.89, 8.29)	7.68 (6.3, 9.05)	8.32 (6.83, 9.78)	9.27 (7.55, 11.14)	11.15 (8.6, 13.56)	13.06 (9.91, 16.24)

Variable	Q10	Q20	Q30	Q40	Q50	Q60	Q70	Q80	Q90
Observations	7,363	7,363	7,363	7,363	7,363	7,363	7,363	7,363	7,363
Imputations	28	28	28	28	28	28	28	28	28

Table S5: Quantile Regression Results. Fully adjusted model, males only (Column E, Table 2 in main paper)

	Variable	Q10	Q20	Q30	Q40	Q50	Q60	Q70	Q80	Q90
	6+ Months Unemployment	0.83 (-0.26, 1.78)	0.79 (-0.13, 1.6)	0.61 (-0.32, 1.52)	0.59 (-0.35, 1.65)	0.75 (-0.33, 1.92)	0.99 (-0.28, 2.51)	1.76 (-0.07, 4.05)	3.22 (0.47, 6.4)	4.16 (1.21, 7.14)
	IMD	-0.01 (-0.03, 0.01)	-0.01 (-0.02, 0.01)	-0.01 (-0.03, 0.01)	-0.01 (-0.03, 0.02)	0 (-0.02, 0.02)	0 (-0.02, 0.02)	0 (-0.03, 0.03)	0 (-0.04, 0.04)	0.01 (-0.04, 0.06)
	Locus of Control	-0.07 (-0.37, 0.25)	-0.06 (-0.35, 0.23)	-0.15 (-0.5, 0.18)	-0.26 (-0.59, 0.11)	-0.22 (-0.54, 0.12)	-0.15 (-0.48, 0.2)	0 (-0.45, 0.44)	0.16 (-0.47, 0.8)	0.32 (-0.46, 1.06)
	GHQ-12 @ Age 14/15	0.06 (-0.13, 0.23)	0.07 (-0.08, 0.2)	0.04 (-0.11, 0.21)	0.08 (-0.09, 0.26)	0.19 (-0.01, 0.38)	0.29 (0.09, 0.47)	0.38 (0.15, 0.67)	0.56 (0.2, 0.93)	0.64 (0.2, 1.09)
	GHQ-12 @ Age 16/17	0.18 (0.12, 0.24)	0.21 (0.15, 0.26)	0.24 (0.18, 0.3)	0.25 (0.19, 0.31)	0.26 (0.19, 0.33)	0.28 (0.21, 0.35)	0.3 (0.21, 0.4)	0.33 (0.2, 0.47)	0.41 (0.24, 0.57)
Self-Rated Health @ Age 14/15	Fairly Good	0.07 (-0.51, 0.68)	0.2 (-0.28, 0.68)	0.25 (-0.29, 0.78)	0.29 (-0.27, 0.82)	0.32 (-0.27, 0.88)	0.21 (-0.44, 0.86)	0.16 (-0.65, 1.02)	0.64 (-0.5, 1.77)	1.38 (-0.18, 2.92)
	Not Very Good	-0.1 (-2.14, 2.2)	0.06 (-2.21, 2.22)	-0.03 (-2.33, 2.48)	-0.03 (-2.37, 2.76)	0.29 (-2.75, 2.87)	0.19 (-2.84, 3.25)	0.02 (-3.23, 5.55)	0.12 (-3.89, 7.7)	2.5 (-4.47, 7.83)
	Not Good at All	1.09 (-4.24, 4.36)	0.76 (-3.2, 4.69)	0.46 (-3.18, 5.69)	0.18 (-3.73, 5.22)	0.08 (-4.13, 5.69)	-0.12 (-4.35, 10.17)	-0.77 (-5.26, 10.26)	-0.85 (-6.44, 9.45)	-1.05 (-6.9, 9.15)
Self-Rated Health @ Age 16/17	Fairly Good	0.29 (-0.37, 0.92)	0.17 (-0.37, 0.71)	0.14 (-0.4, 0.69)	0.19 (-0.41, 0.79)	0.14 (-0.48, 0.77)	0.19 (-0.45, 0.84)	0.28 (-0.59, 1.15)	0.1 (-1.03, 1.21)	0.15 (-1.39, 1.86)
	Not Very Good	0.48 (-1, 1.86)	0.12 (-1.51, 2)	0.46 (-1.5, 2.36)	0.85 (-1.12, 2.26)	0.76 (-0.68, 2.49)	0.85 (-0.83, 3.38)	1.21 (-1.3, 4.2)	1.3 (-1.95, 4.79)	0.53 (-2.83, 4.13)

	Variable	Q10	Q20	Q30	Q40	Q50	Q60	Q70	Q80	Q90
	Not Good at All	0.69 (-4.73, 3.6)	0.83 (-3.31, 3.32)	0.36 (-2.69, 2.98)	0.49 (-2.63, 2.69)	0.58 (-2.96, 6.11)	0.28 (-3.19, 8.45)	0.86 (-3.07, 9.22)	2.01 (-3.52, 8.09)	-1.29 (-5.46, 5.65)
Disabled	Yes, school not affected	0.09 (-0.84, 0.96)	0.08 (-0.66, 0.77)	-0.15 (-0.86, 0.58)	-0.25 (-1.04, 0.64)	-0.11 (-1.03, 0.8)	-0.01 (-0.98, 1.15)	0.18 (-0.97, 1.38)	0.13 (-1.37, 2.26)	1.25 (-1.31, 4.09)
	Yes, school affected	0.68 (-0.86, 1.97)	0.83 (-0.34, 1.93)	0.75 (-0.41, 1.89)	0.69 (-0.49, 1.89)	0.78 (-0.39, 2.02)	0.91 (-0.43, 2.61)	1.35 (-0.6, 3.91)	1.78 (-0.73, 5.54)	3.2 (-0.75, 6.65)
	Risk Behaviours	0 (-0.21, 0.19)	-0.04 (-0.23, 0.16)	-0.03 (-0.23, 0.18)	-0.03 (-0.24, 0.17)	-0.02 (-0.24, 0.24)	0.01 (-0.25, 0.28)	0.01 (-0.32, 0.38)	0.05 (-0.42, 0.63)	0.14 (-0.45, 0.66)
	Attitude to School	0 (-0.05, 0.06)	-0.01 (-0.05, 0.04)	-0.01 (-0.06, 0.04)	-0.02 (-0.06, 0.03)	-0.02 (-0.07, 0.03)	-0.03 (-0.08, 0.02)	-0.05 (-0.12, 0.01)	-0.07 (-0.15, 0.01)	-0.08 (-0.18, 0.03)
	# Waves Bullied, 1-3	0.2 (-0.06, 0.47)	0.15 (-0.06, 0.35)	0.21 (-0.02, 0.45)	0.29 (0.05, 0.52)	0.3 (0.05, 0.54)	0.34 (0.07, 0.61)	0.37 (0.02, 0.73)	0.47 (-0.06, 1.06)	0.71 (-0.01, 1.49)
Qualifications	NVQ 4	0.54 (-0.29, 1.5)	0.45 (-0.23, 1.18)	0.44 (-0.33, 1.17)	0.18 (-0.65, 0.99)	-0.07 (-0.89, 0.79)	-0.06 (-0.91, 0.8)	-0.02 (-1.07, 0.99)	0.1 (-1.52, 1.55)	-0.17 (-2.75, 2.24)
	NVQ 3	0.93 (0.02, 1.87)	0.76 (0.08, 1.45)	0.73 (-0.07, 1.53)	0.62 (-0.27, 1.52)	0.37 (-0.51, 1.28)	0.24 (-0.62, 1.1)	0.09 (-0.96, 1.12)	-0.03 (-1.59, 1.51)	-0.6 (-3.34, 1.87)
	NVQ 2	0.7 (-0.24, 1.7)	0.74 (0.03, 1.44)	0.68 (-0.14, 1.48)	0.39 (-0.51, 1.25)	-0.04 (-0.88, 0.86)	-0.24 (-1.18, 0.69)	-0.31 (-1.45, 0.78)	-0.72 (-2.36, 0.81)	-1.77 (-4.36, 0.62)
	NVQ 1	-0.59 (-1.92, 0.73)	-0.3 (-1.35, 0.72)	-0.18 (-1.26, 0.89)	-0.35 (-1.46, 0.84)	-0.31 (-1.44, 0.89)	-0.2 (-1.43, 1.04)	0 (-1.61, 1.84)	0.52 (-1.78, 3.09)	0.49 (-2.83, 4.04)

	Variable	Q10	Q20	Q30	Q40	Q50	Q60	Q70	Q80	Q90
	No/Other Qual	-0.53 (-1.86, 0.88)	-0.42 (-1.66, 0.96)	-0.13 (-1.53, 1.21)	-0.24 (-1.64, 1.18)	-0.36 (-1.77, 1.1)	-0.24 (-1.8, 1.49)	0.25 (-1.76, 2.67)	0.34 (-2.36, 3.27)	-1.01 (-4.36, 2.66)
Parental NS-SEC	Intermediate	-0.2 (-0.96, 0.56)	-0.36 (-0.96, 0.24)	-0.44 (-1.09, 0.22)	-0.54 (-1.25, 0.17)	-0.51 (-1.27, 0.29)	-0.27 (-1.05, 0.58)	0.12 (-0.89, 1.22)	0.48 (-0.94, 1.97)	0.98 (-1, 3.24)
	Routine	-0.25 (-0.97, 0.49)	-0.22 (-0.82, 0.41)	-0.13 (-0.8, 0.53)	-0.14 (-0.84, 0.54)	-0.17 (-0.86, 0.51)	-0.16 (-0.89, 0.62)	-0.02 (-0.95, 0.95)	0 (-1.3, 1.33)	0.04 (-1.8, 1.96)
	LTU	-0.24 (-1.69, 1.09)	-0.55 (-1.87, 1.14)	-0.39 (-1.95, 1.4)	-0.46 (-2.12, 1.43)	-0.42 (-2.17, 1.44)	-0.29 (-2.18, 1.88)	-0.08 (-2.44, 2.93)	0.54 (-2.67, 4.39)	1.56 (-2.5, 5.76)
Parental Education	Other HE	-0.39 (-1.32, 0.44)	-0.16 (-0.88, 0.54)	-0.18 (-0.98, 0.66)	-0.06 (-0.89, 0.79)	0.15 (-0.75, 0.98)	0.15 (-0.76, 1.08)	0.11 (-1.04, 1.35)	0.18 (-1.37, 1.84)	0.91 (-1.35, 3.62)
	A-Level	0.19 (-0.65, 1)	0.29 (-0.45, 0.98)	0.13 (-0.67, 0.99)	0.26 (-0.61, 1.12)	0.46 (-0.44, 1.34)	0.55 (-0.42, 1.52)	0.46 (-0.69, 1.67)	0.62 (-1.1, 2.31)	1.37 (-0.95, 3.84)
	GCSE A-C	0.23 (-0.67, 1.06)	0.45 (-0.28, 1.16)	0.37 (-0.41, 1.14)	0.32 (-0.48, 1.17)	0.42 (-0.49, 1.25)	0.35 (-0.59, 1.25)	0.2 (-0.93, 1.32)	0.02 (-1.58, 1.5)	-0.14 (-2.43, 2.03)
	Other/None	0.07 (-1, 1.16)	0.27 (-0.67, 1.19)	0.41 (-0.64, 1.5)	0.79 (-0.31, 1.93)	1.21 (0.1, 2.27)	1.33 (0.14, 2.56)	1.67 (0.09, 3.26)	2.1 (0.02, 4.32)	3.42 (0.52, 6.15)
Ethnicity	Mixed	-0.56 (-1.82, 0.7)	-0.46 (-1.8, 0.83)	-0.36 (-1.8, 0.76)	-0.53 (-1.82, 0.55)	-0.84 (-2.01, 0.71)	-0.74 (-2.24, 1.3)	-0.45 (-2.43, 1.78)	-0.55 (-2.77, 2.18)	-0.3 (-3.47, 5.21)
	Indian	-1.28 (-2.3, -0.25)	-0.89 (-1.98, 0.17)	-0.7 (-1.86, 0.52)	-0.59 (-1.79, 0.52)	-0.74 (-1.89, 0.54)	-0.61 (-1.95, 0.96)	-0.41 (-1.96, 1.7)	0.1 (-2.34, 4.45)	1.69 (-2.42, 5.34)

Variable	Q10	Q20	Q30	Q40	Q50	Q60	Q70	Q80	Q90
Pakistani	-0.58 (-2.31, 0.58)	-0.46 (-1.52, 0.44)	-0.84 (-1.93, 0.08)	-1.41 (-2.47, -0.38)	-1.87 (-2.97, -0.59)	-1.84 (-3.17, -0.53)	-1.78 (-3.32, -0.23)	-2.28 (-4.18, -0.28)	-3.17 (-5.89, 0.44)
Bangladeshi	-1.23 (-3.45, 0.67)	-0.67 (-3.02, 1.13)	-0.46 (-2.54, 1.19)	-0.66 (-2.39, 1.06)	-0.92 (-2.74, 0.93)	-0.97 (-2.81, 0.98)	-1.16 (-3.3, 1.14)	-1.93 (-4.41, 0.54)	-3.44 (-6.3, -0.15)
Black African	-0.84 (-3.16, 0.6)	-0.91 (-2.18, 0.38)	-1.15 (-2.5, 0.96)	-0.87 (-2.8, 0.85)	-1.05 (-2.86, 0.42)	-1.63 (-3.16, 0.19)	-2.04 (-4.02, 0.08)	-2.89 (-4.97, -0.36)	-4.23 (-6.67, -0.65)
Black Caribbean	-1.36 (-3.54, 0.94)	-0.07 (-1.71, 1.12)	-0.18 (-1.4, 1.18)	-0.23 (-1.64, 1.15)	-0.23 (-1.74, 1.35)	-0.18 (-1.7, 1.87)	0.09 (-1.83, 2.5)	0.01 (-2.56, 4.32)	-0.89 (-4.21, 4.34)
Other	0.26 (-3.61, 1.92)	0.53 (-1.01, 1.81)	0.41 (-1, 2.57)	0.8 (-1.29, 3.09)	1 (-1.16, 3.3)	1.36 (-0.97, 3.36)	1.28 (-1.06, 3.97)	1.04 (-1.66, 4.99)	-0.11 (-3.26, 4.34)
Constant	3.03 (0.28, 5.34)	4.41 (2.4, 6.27)	5.41 (3.44, 7.29)	6.47 (4.54, 8.33)	7.38 (5.38, 9.4)	8.37 (6.11, 10.66)	9.89 (7, 12.89)	11.75 (7.8, 15.55)	13.71 (8.5, 18.4)
Observations	3,196	3,196	3,196	3,196	3,196	3,196	3,196	3,196	3,196
Imputations	28	28	28	28	28	28	28	28	28

Table S6: Quantile Regression Results. Fully adjusted model, females only (Column F, Table 2 in main paper)

	Variable	Q10	Q20	Q30	Q40	Q50	Q60	Q70	Q80	Q90
	6+ Months Unemployment	-0.49 (-1.55, 0.87)	0.31 (-1.07, 1.35)	0.49 (-0.52, 1.43)	0.46 (-0.53, 1.46)	0.58 (-0.64, 1.96)	1.19 (-0.32, 3.03)	2.11 (0.18, 3.84)	2.3 (0.33, 4.36)	2.48 (0.34, 4.59)
	IMD	0.01 (-0.01, 0.02)	0 (-0.01, 0.02)	0 (-0.01, 0.02)	0 (-0.01, 0.02)	0.01 (-0.01, 0.02)	0.01 (-0.01, 0.03)	0.01 (-0.01, 0.04)	0.02 (-0.01, 0.05)	0.04 (0, 0.09)
	Locus of Control	-0.22 (-0.48, 0.07)	-0.13 (-0.39, 0.13)	-0.08 (-0.33, 0.17)	-0.03 (-0.3, 0.22)	-0.04 (-0.33, 0.25)	-0.09 (-0.43, 0.26)	0 (-0.42, 0.4)	0.04 (-0.5, 0.54)	-0.24 (-0.87, 0.41)
	GHQ-12 @ Age 14/15	0.09 (-0.01, 0.2)	0.13 (0.03, 0.23)	0.13 (0.03, 0.22)	0.14 (0.04, 0.25)	0.18 (0.06, 0.29)	0.22 (0.09, 0.37)	0.25 (0.09, 0.42)	0.3 (0.08, 0.54)	0.45 (0.17, 0.7)
	GHQ-12 @ Age 16/17	0.1 (0.06, 0.15)	0.14 (0.09, 0.19)	0.17 (0.12, 0.21)	0.18 (0.13, 0.23)	0.2 (0.15, 0.26)	0.26 (0.19, 0.33)	0.31 (0.23, 0.38)	0.35 (0.26, 0.44)	0.36 (0.25, 0.46)
Self-Rated Health @ Age 14/15	Fairly Good	0.22 (-0.31, 0.76)	0.25 (-0.21, 0.71)	0.41 (-0.02, 0.84)	0.47 (0.01, 0.91)	0.43 (-0.07, 0.97)	0.51 (-0.06, 1.07)	0.48 (-0.16, 1.1)	0.48 (-0.36, 1.34)	0.81 (-0.33, 1.98)
	Not Very Good	0.86 (-0.9, 2.34)	0.75 (-0.68, 2.21)	1.04 (-0.5, 2.66)	1.45 (-0.27, 2.65)	1.23 (-0.19, 2.74)	1.17 (-0.45, 3.64)	1.72 (-0.52, 5.2)	2.47 (-0.77, 5.54)	2 (-0.98, 4.94)
	Not Good at All	0.08 (-2.2, 1.82)	-0.68 (-2.71, 1.58)	-0.95 (-2.75, 3.35)	-1.01 (-3.36, 4.69)	-0.3 (-4.29, 5.38)	0.58 (-4.7, 6.06)	1.08 (-4.73, 7.59)	0.98 (-4.27, 8.35)	1.8 (-5.52, 7.52)
Self-Rated Health @ Age 16/17	Fairly Good	0.2 (-0.35, 0.71)	0.21 (-0.25, 0.69)	0.14 (-0.29, 0.6)	0.23 (-0.24, 0.7)	0.21 (-0.31, 0.72)	0.06 (-0.55, 0.66)	0.11 (-0.55, 0.77)	0.26 (-0.7, 1.19)	0.27 (-0.89, 1.5)
	Not Very Good	-0.55 (-1.84, 0.4)	-0.26 (-1.45, 0.99)	0.27 (-0.78, 1.29)	0.48 (-0.53, 1.46)	0.53 (-0.58, 1.61)	0.45 (-0.75, 1.85)	0.49 (-0.84, 2.13)	0.3 (-1.42, 2.18)	0.43 (-2.22, 3.01)

	Variable	Q10	Q20	Q30	Q40	Q50	Q60	Q70	Q80	Q90
	Not Good at All	-1.04 (-3.03, 1.79)	-0.51 (-3.23, 1.92)	-0.48 (-2.67, 2.33)	0.06 (-2.55, 3.24)	0.78 (-2.6, 4.77)	1.04 (-2.6, 6.02)	1.62 (-2.64, 5.78)	1.19 (-2.77, 6.38)	1.46 (-3.71, 7.18)
Disabled	Yes, school not affected	0.17 (-0.85, 0.98)	0.19 (-0.57, 0.97)	0.12 (-0.61, 0.86)	0.07 (-0.75, 1.06)	0.46 (-0.69, 1.75)	0.95 (-0.35, 2.26)	1.25 (-0.14, 2.87)	1.48 (-0.16, 3.8)	1.79 (-0.63, 4.17)
	Yes, school affected	0.14 (-1.32, 1.33)	-0.07 (-1.2, 0.96)	-0.08 (-1.15, 0.96)	-0.07 (-1.2, 1.19)	0.26 (-1.05, 1.52)	0.38 (-0.91, 1.72)	0.32 (-1.14, 2.42)	0.79 (-1.48, 3.39)	1.72 (-1.13, 4.66)
	Risk Behaviours	-0.03 (-0.23, 0.1)	-0.14 (-0.27, 0.01)	-0.2 (-0.35, 0.04)	-0.13 (-0.37, 0.12)	-0.06 (-0.3, 0.17)	-0.03 (-0.26, 0.23)	0 (-0.26, 0.28)	0.03 (-0.33, 0.43)	-0.08 (-0.51, 0.34)
	Attitude to School	-0.04 (-0.07, 0)	-0.03 (-0.06, 0)	-0.03 (-0.07, 0)	-0.03 (-0.07, 0.01)	-0.02 (-0.06, 0.02)	-0.01 (-0.05, 0.04)	-0.01 (-0.07, 0.04)	-0.02 (-0.11, 0.05)	-0.03 (-0.12, 0.06)
	# Waves Bullied, 1-3	-0.03 (-0.25, 0.19)	0.08 (-0.12, 0.27)	0.13 (-0.08, 0.33)	0.17 (-0.03, 0.37)	0.23 (0.02, 0.44)	0.34 (0.09, 0.58)	0.46 (0.15, 0.77)	0.61 (0.2, 1.01)	0.68 (0.14, 1.21)
Qualifications	NVQ 4	0.47 (-0.3, 1.18)	0.3 (-0.27, 0.92)	0.38 (-0.2, 0.99)	0.48 (-0.11, 1.06)	0.54 (-0.09, 1.16)	0.7 (-0.03, 1.4)	0.71 (-0.2, 1.54)	0.45 (-0.79, 1.6)	0.39 (-1.11, 1.92)
	NVQ 3	0.24 (-0.57, 0.99)	0.03 (-0.57, 0.65)	-0.05 (-0.66, 0.57)	0.06 (-0.58, 0.7)	0.28 (-0.41, 0.98)	0.54 (-0.25, 1.39)	0.67 (-0.27, 1.54)	0.5 (-0.79, 1.8)	0.8 (-0.79, 2.58)
	NVQ 2	-0.18 (-1.03, 0.67)	-0.29 (-0.97, 0.4)	-0.24 (-0.86, 0.38)	-0.17 (-0.83, 0.51)	-0.03 (-0.77, 0.68)	0.05 (-0.78, 0.88)	0.1 (-0.92, 1.06)	-0.33 (-1.78, 1.05)	-0.85 (-2.51, 0.89)
	NVQ 1	-0.3 (-1.61, 0.78)	-0.47 (-1.42, 0.59)	-0.31 (-1.27, 0.69)	-0.19 (-1.21, 0.86)	-0.07 (-1.2, 1.11)	0.29 (-1.04, 1.52)	0.49 (-0.96, 2.06)	0.63 (-1.44, 2.55)	0.04 (-2.22, 2.6)

	Variable	Q10	Q20	Q30	Q40	Q50	Q60	Q70	Q80	Q90
	No/Other Qual	0.17 (-1.3, 1.42)	-0.01 (-0.95, 1.06)	-0.07 (-1.11, 1.01)	0.02 (-1.04, 1.2)	0.25 (-0.99, 1.51)	0.6 (-0.76, 2.07)	1.1 (-0.69, 3.3)	1.34 (-1.13, 3.68)	0.45 (-2.1, 3.1)
Parental NS-SEC	Intermediate	0.5 (-0.16, 1.21)	0.45 (-0.13, 1.01)	0.46 (-0.08, 0.97)	0.37 (-0.19, 0.94)	0.3 (-0.32, 0.91)	0.09 (-0.66, 0.82)	-0.14 (-0.93, 0.7)	-0.14 (-1.17, 0.98)	0.05 (-1.52, 1.58)
	Routine	0.62 (0.02, 1.29)	0.72 (0.18, 1.26)	0.81 (0.24, 1.35)	0.8 (0.22, 1.37)	0.81 (0.15, 1.46)	0.83 (0.02, 1.62)	0.81 (-0.03, 1.67)	1.17 (0.07, 2.3)	1.44 (-0.09, 2.94)
	LTU	-0.33 (-3, 1.53)	0.08 (-1.51, 1.42)	0.1 (-1.27, 1.45)	0.19 (-1.23, 1.89)	0.58 (-1.19, 2.49)	1.07 (-0.95, 3.32)	1.81 (-0.34, 4.17)	2.21 (-0.37, 5)	2.23 (-0.75, 5.23)
Parental Education	Other HE	-0.04 (-0.83, 0.67)	-0.11 (-0.79, 0.55)	-0.15 (-0.84, 0.58)	-0.01 (-0.78, 0.75)	-0.01 (-0.83, 0.8)	0 (-0.91, 0.89)	0.08 (-0.95, 1.07)	0.14 (-1.15, 1.44)	0.11 (-1.59, 1.76)
	A-Level	-0.39 (-1.24, 0.34)	-0.58 (-1.26, 0.09)	-0.73 (-1.38, -0.04)	-0.71 (-1.45, -0.01)	-0.86 (-1.64, -0.06)	-0.89 (-1.8, 0.01)	-1.03 (-2.03, -0.04)	-1.36 (-2.59, -0.15)	-1.76 (-3.53, 0.09)
	GCSE A-C	-0.42 (-1.24, 0.32)	-0.49 (-1.17, 0.17)	-0.5 (-1.15, 0.14)	-0.54 (-1.21, 0.14)	-0.68 (-1.45, 0.15)	-0.55 (-1.5, 0.44)	-0.43 (-1.42, 0.56)	-0.45 (-1.72, 0.89)	-0.5 (-2.3, 1.26)
	Other/None	-0.3 (-1.31, 0.6)	-0.25 (-1.07, 0.54)	-0.38 (-1.17, 0.43)	-0.36 (-1.22, 0.48)	-0.44 (-1.38, 0.57)	-0.46 (-1.6, 0.71)	-0.69 (-1.97, 0.6)	-0.89 (-2.55, 0.75)	-0.88 (-2.96, 1.34)
Ethnicity	Mixed	-0.78 (-2.17, 0.52)	-0.66 (-1.82, 0.36)	-0.73 (-1.79, 0.24)	-0.95 (-2, 0.24)	-0.99 (-2.19, 0.5)	-0.74 (-2.3, 1.28)	0.17 (-2.15, 3.6)	1.61 (-1.3, 5.99)	3.46 (-0.87, 8.99)
	Indian	-1.22 (-2.47, -0.06)	-0.93 (-1.86, -0.2)	-1.01 (-1.78, -0.1)	-0.81 (-1.77, 0.12)	-0.59 (-1.59, 0.36)	-0.51 (-1.47, 0.53)	-0.27 (-1.5, 1.3)	0.16 (-1.5, 1.83)	-0.45 (-2.45, 1.82)

Variable	Q10	Q20	Q30	Q40	Q50	Q60	Q70	Q80	Q90
Pakistani	-0.75 (-1.99, 0.44)	-0.56 (-1.81, 0.44)	-0.4 (-1.4, 0.51)	-0.44 (-1.46, 0.57)	-0.52 (-1.61, 0.72)	-0.34 (-1.64, 1.03)	0.02 (-1.49, 1.67)	0 (-1.9, 2.07)	-0.34 (-2.96, 2.46)
Bangladeshi	-2.56 (-4.15, -0.34)	-1.72 (-3.95, -0.21)	-1.19 (-2.63, -0.17)	-1.22 (-2.39, 0.04)	-1.24 (-2.64, 0.21)	-1.26 (-2.79, 0.1)	-1.52 (-2.97, 0.22)	-1.85 (-3.99, 0.38)	-2.53 (-5.46, 1.31)
Black African	-0.24 (-1.27, 1)	-0.03 (-1.21, 0.93)	-0.3 (-1.24, 0.88)	-0.34 (-1.57, 1.48)	0.02 (-1.73, 2.05)	0.36 (-1.5, 2.26)	0.3 (-1.52, 2.51)	0.26 (-2.02, 3.37)	0.56 (-2.79, 4.85)
Black Caribbean	-2.4 (-3.58, -1.02)	-1.96 (-3.07, -1.04)	-1.93 (-2.96, -0.87)	-1.8 (-2.95, -0.46)	-1.53 (-3, -0.18)	-1.33 (-2.78, 0.05)	-1.29 (-2.71, 0.2)	-1.53 (-3.32, 0.32)	-2.37 (-4.63, 0.3)
Other	-1.31 (-3.64, 0.12)	-1.41 (-2.77, 0.23)	-0.98 (-2.67, 0.71)	-0.52 (-2.37, 1.32)	-0.02 (-2.08, 1.5)	0.13 (-1.69, 2.43)	0.82 (-1.37, 2.94)	0.76 (-1.56, 3.49)	-0.04 (-2.82, 2.4)
Constant	5.87 (4.36, 7.39)	6.63 (5.1, 8.15)	7.57 (6.12, 8.95)	7.89 (6.38, 9.59)	8.21 (6.23, 10.11)	7.98 (5.92, 9.97)	8.58 (6.26, 11)	10.06 (6.65, 13.69)	12.55 (8.41, 16.47)
Observations	4,167	4,167	4,167	4,167	4,167	4,167	4,167	4,167	4,167
Imputations	28	28	28	28	28	28	28	28	28

References

Buddelmeyer, H., & Powdthavee, N. (2016). Can having internal locus of control insure against negative shocks? Psychological evidence from panel data. *Journal of Economic Behavior & Organization*, *122*, 88–109. https://doi.org/10.1016/j.jebo.2015.11.014

Caliendo, M., Cobb-Clark, D. A., & Uhlendorff, A. (2015). Locus of control and job search strategies. *Review of Economics and Statistics*, *97*(1), 88–103. https://doi.org/10.1162/REST_a_00459

Chowdry, H., Crawford, C., & Goodman, A. (2011). The role of attitudes and behaviours in explaining socio-economic differences in attainment at age 16. *Longitudinal and Life Course Studies*, *2*(1). https://doi.org/10.14301/llcs.v2i1.141

Cobb-Clark, D. A. (2015). Locus of control and the labor market. *IZA Journal of Labor Economics*, *4*(1), 3. https://doi.org/10.1186/s40172-014-0017-x

Cobb-Clark, D. A., Kassenboehmer, S. C., & Schurer, S. (2014). Healthy habits: The connection between diet, exercise, and locus of control. *Journal of Economic Behavior & Organization*, *98*, 1–28. https://doi.org/10.1016/j.jebo.2013.10.011

Cobb-Clark, D. A., & Schurer, S. (2013). Two economists' musings on the stability of locus of control. *Economic Journal*, *123*(570). https://doi.org/10.1111/ecoj.12069

Crawford, C., Dearden, L., & Greaves, E. (2011). *Does when you are born matter?The impact ofmonthof birth onchildren's cognitive and non-cognitive skills in England*. Institue for Fiscal Studies. https://www.ifs.org.uk/bns/bn122.pdf

Department for Education, Crawford, C., & Cribb, J. (2012). *Gap year takers: Uptake, trends and long term outcomes*. Department for Education. https://assets.publishing.service.gov.uk/government/uploads/system/uploads/attachment_data/file/219637/DFE-RR252.pdf https://www.gov.uk/government/publications/gap-year-takers-uptake-trends-and-long-term-outcomes

Gladwell, D., Popli, G., & Tsuchiya, A. (2016). *A Dynamic Analysis of Skill Formation and NEET status*. https://cemapre.iseg.ulisboa.pt/educonf/4e3/files/Papers/Popli.pdf

Hankins, M. (2008). The factor structure of the twelve item General Health Questionnaire (GHQ-12): The result of negative phrasing? *Clinical Practice and Epidemiology in Mental Health*, *4*(1), 10. https://doi.org/10.1186/1745-0179-4-10

Mendolia, S., & Walker, I. (2014a). THE EFFECT OF NONCOGNITIVE TRAITS ON HEALTH BEHAVIOURS IN ADOLESCENCE: THE EFFECT OF NONCOGNITIVE TRAITS ON HEALTH BEHAVIOURS IN ADOLESCENCE. *Health Economics*, *23*(9), 1146–1158. https://doi.org/10.1002/hec.3043

Mendolia, S., & Walker, I. (2014b). The effect of personality traits on subject choice and performance in high school: Evidence from an English cohort. *Economics of Education Review*, *43*, 47–65. https://doi.org/10.1016/j.econedurev.2014.09.004

Mendolia, S., & Walker, I. (2015). Youth unemployment and personality traits. *IZA Journal of Labor Economics*, *4*(1). https://doi.org/10.1186/s40172-015-0035-3

Ng-Knight, T., & Schoon, I. (2017). Can Locus of Control Compensate for Socioeconomic Adversity in the Transition from School to Work? *Journal of Youth and Adolescence*, *46*(10), 2114–2128. https://doi.org/10.1007/s10964-017-0720-6

Piatek, R., & Pinger, P. (2016). Maintaining (Locus of) Control? Data Combination for the Identification and Inference of Factor Structure Models. *Journal of Applied Econometrics*, *31*(4), 734–755. https://doi.org/10.1002/jae.2456

Rotter, J. B. (1966). Generalized expectancies for internal versus external control of reinforcement. *Psychological Monographs*, *80*(1), 1–28. https://doi.org/10.1037/h0092976

Rotter, J. B. (1975). Some problems and misconceptions related to the construct of internal versus external control of reinforcement. *Journal of Consulting and Clinical Psychology*, *43*(1), 56–67. https://doi.org/10.1037/h0076301

Wijedasa, D. (2017). 'People like me don't have much of a chance in life': Comparing the locus of control of young people in foster care with that of adoptees, children from disadvantaged backgrounds and children in the general population. *Adoption & Fostering*, *41*(1), 5–19. https://doi.org/10.1177/0308575916684299
