# Supplementary material for: Heterogeneity in the association between youth unemployment and mental health later in life: a quantile regression analysis of longitudinal data from English schoolchildren
Source: BMJ Open. 2021 Jul 19;11(7):e047997. doi: 10.1136/bmjopen-2020-047997 (PMC8291333; doi:10.1136/bmjopen-2020-047997)
Supplement: Author's manuscript [file bmjopen-2020-047997.draft_revisions.pdf]

BMJ Open

BMJ Open is committed to open peer review. As part of this commitment we make the peer review history of every article we publish publicly available.

When an article is published we post the peer reviewers' comments and the authors' responses online. We also post the versions of the paper that were used during peer review. These are the versions that the peer review comments apply to.

The versions of the paper that follow are the versions that were submitted during the peer review process. They are not the versions of record or the final published versions. They should not be cited or distributed as the published version of this manuscript.

BMJ Open is an open access journal and the full, final, typeset and author-corrected version of record of the manuscript is available on our site with no access controls, subscription charges or pay-per-view fees (http://bmjopen.bmj.com).

If you have any questions on BMJ Open's open peer review process please email info.bmjopen@bmj.com

BMJ Open

Heterogeneity in the Association Between Youth Unemployment and Mental Health Later in Life: A Quantile Regression Analysis of Longitudinal Data from English Schoolchildren

Journal:	*BMJ Open*
Manuscript ID	bmjopen-2020-047997
Article Type:	Original research
Date Submitted by the Author:	14-Dec-2020

Complete List of Authors:	Wright, Liam; University College London, Department of Epidemiology and Public Health<br>Head, Jenny; University College London, Department of Epidemiology and Public Health<br>Jivraj, Stephen ; University College London, Departmnent of Epidemiology and Public Health

Keywords:	MENTAL HEALTH, PUBLIC HEALTH, Depression & mood disorders < PSYCHIATRY

SCHOLARONE™
Manuscripts

I, the Submitting Author has the right to grant and does grant on behalf of all authors of the Work (as defined in the below author licence), an exclusive licence and/or a non-exclusive licence for contributions from authors who are: i) UK Crown employees; ii) where BMJ has agreed a CC-BY licence shall apply, and/or iii) in accordance with the terms applicable for US Federal Government officers or employees acting as part of their official duties; on a worldwide, perpetual, irrevocable, royalty-free basis to BMJ Publishing Group Ltd ("BMJ") its licensees and where the relevant Journal is co-owned by BMJ to the co-owners of the Journal, to publish the Work in this journal and any other BMJ products and to exploit all rights, as set out in our licence.

The Submitting Author accepts and understands that any supply made under these terms is made by BMJ to the Submitting Author unless you are acting as an employee on behalf of your employer or a postgraduate student of an affiliated institution which is paying any applicable article publishing charge ("APC") for Open Access articles. Where the Submitting Author wishes to make the Work available on an Open Access basis (and intends to pay the relevant APC), the terms of reuse of such Open Access shall be governed by a Creative Commons licence – details of these licences and which Creative Commons licence will apply to this Work are set out in our licence referred to above.

Other than as permitted in any relevant BMJ Author's Self Archiving Policies, I confirm this Work has not been accepted for publication elsewhere, is not being considered for publication elsewhere and does not duplicate material already published. I confirm all authors consent to publication of this Work and authorise the granting of this licence.

For peer review only

Heterogeneity in the Association Between Youth Unemployment and Mental Health Later in Life: A Quantile Regression Analysis of Longitudinal Data from English Schoolchildren

Liam Wright, Jenny Head and Stephen Jivraj

Department of Epidemiology and Public Health, University College London

Corresponding Author: Liam Wright, 1-19 Torrington Place, London WC1E 7HB, liam.wright.17@ucl.ac.uk

Word count: 3,315

For peer review only

For peer review only - http://bmjopen.bmj.com/site/about/guidelines.xhtml

Heterogeneity in the Association Between Youth Unemployment and Mental Health Later in Life: A Quantile Regression Analysis of Longitudinal Data from English Schoolchildren

Abstract

Objectives: An association between youth unemployment and poorer mental health later in life has been found in several countries. Little is known about whether this association is consistent across individuals or differs in strength. We adopt a quantile regression approach to explore heterogeneity in the association between youth unemployment and later mental health along the mental health distribution. **Design:** Prospective longitudinal cohort of secondary schoolchildren in England followed from age 13/14 in 2004 to age 25 in 2015. **Setting:** England, United Kingdom. **Participants:** 7,707 participants interviewed at age 25. **Primary and Secondary Outcome Measures:** 12-Item General Health Questionnaire Likert (GHQ) score, a measure of minor psychiatric morbidity. **Results:** Youth unemployment was related to worse mental health at age 25. The association was several times stronger at deciles of GHQ representing the poorest levels of mental health. This association was only partly attenuated when adjusting for confounding variables and for current employment status. The results were robust to choice of control variables. **Conclusions:** There is heterogeneity in the longitudinal association between youth unemployment and mental health, with associations more pronounced at higher levels of psychological ill-health. Youth unemployment may signal clinically relevant future psychological problems among some individuals.

Keywords: Youth Unemployment; School-to-Work Transition; Mental Health; Life Course Epidemiology; Unemployment Scarring; Quantile Regression.

Strengths and Limitations of this Study

- The study includes longitudinal data from a large sample of young English adults, enabling us to control for several background factors that may explain selection into unemployment.
- To our knowledge, this is the first study on the long-term association between youth unemployment and later mental health using data from adults who entered the adulthood during the aftermath of the Global Financial Crisis.
- We use quantile regression to study whether the association is heterogeneous or consistent across all individuals.
- We run thousands of models to test whether are results are robust to alternative model specifications.
- While we control for several adolescent factors, we use observational data so results may not indicate causality.

For peer review only - http://bmjopen.bmj.com/site/about/guidelines.xhtml

INTRODUCTION

The pandemic of coronavirus disease 2019 (COVID-19) looks set to plunge the world into recession and unemployment rates have begun to rise sharply across the globe [1]. A large body of research finds that unemployed individuals have worse mental health and wellbeing than their peers, with evidence from longitudinal and quasi-experiemental studies suggesting the association between unemployment and mental health is causal [2,3,though also see 4]. Several theories have been proposed for why unemployment may negatively affect mental health [5], including loss of material and non-material ("latent") benefits of employment [6]. Given this literature, there is concern that the increasing unemployment rates will lead to higher rates of mental ill health [7].

The effect of unemployment on mental health may not be transient. Several studies suggest that unemployment "scars", defined as an effect of (prior) unemployment and poor mental health persisting even upon re-employment [8]. A focus of the literature has been on the long-term effect of youth unemployment specifically, with studies from the United States [9], the United Kingdom [10], Canada [11], and Sweden [12,13] finding associations between early unemployment and subjective wellbeing, depression, and anxiety symptoms measured decades later. These associations remain (where tested) after adjusting for adolescent mental health. This suggests that associations cannot be explained by health-related selection *into* unemployment.

Focusing on youth unemployment (typically defined as unemployment before age 25) is valuable for two reasons. First, unemployment rates are higher among young people and recessions have a disproportionate impact on the job prospects of younger workers [10]. Second, adolescence and young adulthood are important from a life course perspective - unemployment at these ages may have larger long-term effects. Unemployment early in the transition to adulthood has been argued to hinder maturation into normative adult roles and identity [12] and to set off "chains of risk" [14], with initial disadvantage begetting further disadvantage, such as increased future unemployment risk and lower lifetime wages [15,16] - factors which are themselves related to poorer mental health. Adolescence and early adulthood are also sensitive periods of physiological and neurobehavioural development [14,17]. Stressors experienced during this age may have long term effects on physiology and stress responses, negatively impacting lifelong physical and mental health.

While an overall association between youth unemployment and mental health later in life appears well established, little empirical research has explored the reasons behind such a link. Further, little research has studied whether scarring is uniform across all formerly unemployed individuals or whether effects differ in strength or are confined to particular individuals. Yet, such information is useful for directing resources towards the most vulnerable and for providing insight into the life-course processes that may generate long-term effects.

In this paper, we extend the literature by using quantile regression to investigate heterogeneity in the strength of these long-term effects. Unlike in typical ordinary least squares regression, which estimates changes in the conditional mean of a dependent variable, quantile regression estimates changes at specified quantiles of the distribution (i.e. 10th percentile, median, etc.) [18]. Quantile regression thus allows researchers to model change in both the location and shape of the distribution of a dependent variable in response to changes in other variables, and, accordingly, to see whether associations differ in direction or strength across the distribution.

A small number of studies have used quantile regression to investigate heterogeneity in the contemporary association between unemployment and mental health using data from working-age adults [19–21]. Only one study has looked at long-term effects but has looked at life satisfaction, specifically [22]. Each of these studies finds that the negative association between unemployment and mental health is stronger at poorest levels of mental health or wellbeing. This is consistent with studies

For peer review only

of trajectories in mental wellbeing which show that responses to episodes of unemployment are not uniform, with a minority of individuals appearing more strongly affected [23,24].

We hypothesise that long-term effects will be heterogeneous and that they will be strongest at quantiles representing the poorest levels of mental health. We reason that the strength of the proposed pathways linking youth unemployment to later mental health are likely to differ across individuals. For instance, the consequences of early unemployment for later labour market outcomes (i.e. "chains of risk") is unlikely to be uniform. A recent German study finds that the scarring effect of youth unemployment on future unemployment is primarily driven by a minority of formerly unemployed individuals experiencing particularly long periods of later unemployment [16], while the literature on "compensatory disadvantage" shows that some social adversities can be overcome by individuals with sufficient socioeconomic resources [25]. Further, studies of psychological resilience shows that many individuals experience little psychological injury in response to many life stressors [26] – job-loss included [24,27] – while others experience larger and longer-lasting effects. This literature also provides some evidence that those with pre-existing mental health problems are more negatively affected by adverse events [28].

In this paper, we also test whether associations differ by gender. Previous research shows that the contemporary effect of unemployment is typically greater among males [2], though studies using more recent data in contexts of greater female labour force participation do not find differences by gender [29]. We also test whether long-term associations remain after adjusting for current economic activity, in line with original conceptions of scarring as a long-term effect that is independent of later employment status [8].

METHODS

Sample

We use data from Next Steps (formerly, the Longitudinal Study of Young People in England), a cohort of English schoolchildren recruited at age 13/14 in 2003/04 [30]. The cohort were followed annually for seven years from age 13/14 to age 19/20 and surveyed again at age 25 in 2014/15. Participants were interviewed at each wave, with their primary and secondary caregivers also interviewed for the first four waves.

Participants were recruited using a two-stage stratified sampling design with participants selected from a sample of state and independent schools. 15,7770 individuals were originally recruited with individuals from ethnic minority backgrounds and schools with a high proportion of students on free school meals over-sampled (response rate: 74%). A sample boost of 352 ethnic minority participants was also added in age 16/17. From 2004-2010, only previous responders were followed across waves and per wave drop-out varied between 8-14%. At the 2014/15 wave, efforts were made to contact previous non-responders. Our sample is all individuals who participated at the age 25 survey (n = 7,707; 47.8% of total sample).

Measures

Mental Health at Age 25

We measure mental health at age 25 using the 12-Item General Health Questionnaire (GHQ-12). The GHQ was developed as a screening tool for minor non-psychotic psychiatric morbidity in the general population [31] and has been found to be a valid measure of mood and anxiety disorders [32]. Items relate to the appearance of new, distressing phenomena and reduced functioning due to psychiatric problems compared with usual experience. Each item is scored on a four-point scale. We use the summed Likert score as our outcome variable. Higher scores indicate worse mental health (range 0-36).

An issue with this measure is that by using usual experience as the reference condition, the GHQ may not adequately capture chronic problems or long-term effects of unemployment [33]. However, a recent

For peer review only - http://bmjopen.bmj.com/site/about/guidelines.xhtml

validation study finds that the GHQ displays good sensitivity for detecting depression [32] and in the Supplementary Information, we show visual evidence from the United Kingdom Household Longitudinal Study that GHQ scores are related to long-term depression.

Youth Unemployment

We measure youth unemployment using a binary indicator for whether the participant experienced 6+ months (continuous) unemployment between October 2008 - May 2010 (approximately ages 18-20). This period corresponds to the first twenty months following the summer holiday after the normative end date of further education for this cohort. It also overlaps with the beginning of the Global Financial Crisis, following which youth unemployment rates rose precipitously worldwide [10]. We derive the variable from activity history data which participants provided at each wave on main activities carried out since their previous interview.

Covariates

We add several control variables to partly account for non-random selection into unemployment. To account for mental health-related selection, we use scores from the 12-item GHQ at ages 14/15 and 16/17. We use the *Caseness* score at age 14/15 (1 if has experienced the symptom more than usual, 0 otherwise; range 0-12) as participants were able to respond "don't know" to each item at this interview (we assume this reflects not experiencing the symptom). We use the Likert score at age 16/17 (range 0-36). The GHQ-12 has been shown to have acceptable validity in adolescent samples [29,30].

To account for physical health-related selection, we control for self-rated health at ages 14/15 and 16/17 (categories: very good, fairly good, not very good, not good at all) and whether the participant had a disability at age 14/15 or 15/16 (categories: no disability; disability, but schooling unaffected; disabled and schooling affected).

We also adjust for gender and ethnicity (categories: White, mixed, Indian, Pakistani, Bangladeshi, Black African, Black Caribbean, other), and to account for differences in social background, we include variables for the participant's family social class (categories: higher, intermediate, routine/manual, long-term unemployed) and highest parental education (further education or higher vs secondary or lower), both measured when the participant was aged 13/14. We also include a measure of neighbourhood deprivation at age 14/15, the Index of Multiple Deprivation 2004 (IMD), which is produced by the UK Government and captures local area deprivation across seven dimensions (income, employment, health, education, barriers to housing and services, living environment and crime). We use quintiles of IMD, with higher quintiles indicating greater deprivation [34].

To account for differences in human capital, we control for educational attainment at age 25 (six categories: National Vocational Qualification levels 1-5, no qualifications). (Education data is not publicly available for ages prior to this.) Social adjustment and non-cognitive skills are likely to cause both labour market outcomes and mental health. As proxies for some of these, we include variables for positive attitude to school (age 14/15; summed response to 12-item measure, range 0-48), risk behaviours (age 13/14; summed response to 8-item measure on anti-social behaviour, alcohol, smoking and drug use in previous 12 months, range 0-8), and bullying victimisation (number of waves reported being bullied in prior 12 months, age 13/14 – 15/16, range 0-3). We also include a measure of *internal* locus of control (beliefs about the extent to which the participant controls their life), produced from confirmatory factor analysis of six questionnaire item. Further detail on this measure is provided in the Supplementary Information.

Current economic activity at age 25 was derived from a question on main activity (categories: employed, education, unemployed, inactive).

For peer review only

Statistical Analysis

We use quantile regression to estimate the association between youth unemployment and GHQ scores at age 25 at each decile of the outcome variable. We run unadjusted models, covariate adjusted models, and covariate adjusted models with current economic activity as a further control variable. We use bootstrapping to estimate confidence intervals (500 replications) and apply survey weights supplied with the data to account for differences in the likelihood of recruitment to, and exit from, the study. To account for item-missingness, we use multiple imputation (m = 28, burn-in=10), adding the survey weights as linear terms in the imputation models to partly account for weighting in our final analysis [35]. We use data from all sample members interviewed at age 25 in the imputations, but discard participants with missing outcome data in the quantile regressions (final sample n = 7,363). We conduct multiple imputations separately for male and female participants.

A potential issue with our analysis is that some control variables may bias associations. For instance, education may be a mediator and the use of proxies for social adjustment and non-cognitive skills could open backdoor paths between youth unemployment and later mental health [36]. Therefore, as a sensitivity analysis, we run a random selection of 20,000 models drawn from all possible combinations of the covariates defined above. To minimise the computational cost, we use only a single imputed dataset for these regressions.

Multiple imputations [37] and data analysis were carried out in R version 3.6.3 [38]. The code to replicate this analysis is available at https://osf.io/3gwap/. Next Steps data are publicly available through the UK Data Service [30]. Ethical approval for Next Steps has been gained from the National Health Service (NHS) Research Ethics Committee (REC) system. This is a secondary data analysis of Next Steps so ethical approval was not required. The public were not involved in the design, or conduct, or reporting, or dissemination plans of our research.

RESULTS

Descriptive Statistics

Descriptive statistics are displayed in Table 1. Individuals with 6+ months unemployment between ages 18-20 differ from those with less unemployment experience along a number of dimensions, including mental and physical health and socio-economic background. This is consistent with other studies showing that selection into youth unemployment is not random [39,40]. There is little difference according to GHQ scores at age 16/17, which may suggest measurement error in this variable.

Table 1: Descriptive statistics.

		Unweighted Observed Data			Weighted Imputed Data	
	Variable	<6 + Months Unemployment	6+ Months Unemployment	% Missing	<6 + Months Unemployment	6+ Months Unemployment
	n	6,589 (91.76%)	592 (8.24%)		6,599.90 (88.03%)	897.13 (11.97%)
	GHQ-12 @ Age 25	11.53 (6)	13.42 (7.59)	4.46%	11.71 (6.19)	13.62 (7.65)*
Gender	Male	2,799 (42.48%)	317 (53.55%)	0%	3,183.39 (48.23%)	538.54 (60.03%)*
	Female	3,790 (57.52%)	275 (46.45%)		3,416.51 (51.77%)	358.59 (39.97%)

For peer review only

	IMD	22.56 (16.76)	30.42 (18.13)	8.78%	22.18 (16.18)	30.08 (18.26)*
	Locus of Control	0.05 (0.97)	-0.51 (1.14)	12.72%	-0.05 (1.01)	-0.64 (1.13)*
Current Economic Activity	Employed	5,569 (85.19%)	370 (62.93%)	1.12%	5,499.25 (83.32%)	521.59 (58.14%)*
	Education	307 (4.7%)	11 (1.87%)		271.68 (4.12%)	12.64 (1.41%)
	Inactive	402 (6.15%)	92 (15.65%)		523.71 (7.94%)	182.18 (20.31%)
	Unemployed	259 (3.96%)	115 (19.56%)		305.25 (4.63%)	180.73 (20.15%)
	GHQ-12 @ Age 14/15	1.74 (2.53)	2.06 (2.76)	13.16%	1.76 (2.58)	2.1 (2.72)*
	GHQ-12 @ Age 16/17	10.5 (5.92)	10.29 (6.48)	20.85%	10.34 (5.98)	10.3 (6.53)
Self-Rated Health @ Age 14/15	Very Good	2,576 (45.54%)	166 (34.51%)	15.22%	2,902.29 (43.97%)	306.18 (34.13%)*
	Fairly Good	2,903 (51.32%)	288 (59.88%)		3,401.69 (51.54%)	520.99 (58.07%)
	Not Very Good	150 (2.65%)	21 (4.37%)		238.64 (3.62%)	58.41 (6.51%)
	Not Good at All	28 (0.49%)	6 (1.25%)		57.27 (0.87%)	11.55 (1.29%)
Self-Rated Health @ Age 16/17	Very Good	2,922 (51.97%)	215 (43.26%)	15.67%	3,315.12 (50.23%)	384.90 (42.9%)*
	Fairly Good	2,308 (41.05%)	246 (49.5%)		2,736.67 (41.47%)	440.17 (49.06%)
	Not Very Good	329 (5.85%)	29 (5.84%)		440.10 (6.67%)	57.85 (6.45%)
	Not Good at All	63 (1.12%)	7 (1.41%)		108.00 (1.64%)	14.21 (1.58%)
Disabled	No	5,619 (87.7%)	466 (80.48%)	2.76%	5,633.49 (85.36%)	701.73 (78.22%)*
	Yes, school not affected	476 (7.43%)	47 (8.12%)		556.52 (8.43%)	74.86 (8.34%)
	Yes, school affected	312 (4.87%)	66 (11.4%)		409.89 (6.21%)	120.54 (13.44%)
	Risk Behaviours	0.76 (1.31)	1.18 (1.65)	12.24%	0.96 (1.5)	1.47 (1.84)*
	Attitude to School	33.49 (7.19)	30.1 (8.02)	10.72%	32.28 (7.72)	28.33 (8.26)*
	# Waves Bullied, 1-3	1.34 (1.15)	1.61 (1.15)	15.47%	1.47 (1.15)	1.72 (1.13)*
Qualifications	NVQ 5	1,167 (17.71%)	19 (3.21%)	0%	927.05 (14.05%)	16.90 (1.88%)*

	NVQ 4	1,846 (28.02%)	57 (9.63%)		1,611.71 (24.42%)	65.08 (7.25%)
	NVQ 3	1,355 (20.56%)	72 (12.16%)		1,147.44 (17.39%)	70.78 (7.89%)
	NVQ 2	1,350 (20.49%)	189 (31.93%)		1,613.46 (24.45%)	252.11 (28.1%)
	NVQ 1	497 (7.54%)	165 (27.87%)		815.63 (12.36%)	338.13 (37.69%)
	No/Other Qual	374 (5.68%)	90 (15.2%)		484.60 (7.34%)	154.14 (17.18%)
Parental NS-SEC	Higher	2,393 (41.27%)	117 (22.72%)	12.33%	2,503.64 (37.93%)	169.41 (18.88%)*
	Intermediate	1,208 (20.83%)	87 (16.89%)		1,393.86 (21.12%)	127.20 (14.18%)
	Routine	1,889 (32.58%)	259 (50.29%)		2,363.27 (35.81%)	516.22 (57.54%)
	LTU	308 (5.31%)	52 (10.1%)		339.13 (5.14%)	84.30 (9.4%)
Parental Education	Degree	1,080 (19.44%)	52 (10.28%)	15.71%	1,098.52 (16.64%)	67.68 (7.54%)*
	Other HE	939 (16.9%)	64 (12.65%)		1,042.91 (15.8%)	91.76 (10.23%)
	A-Level	973 (17.51%)	77 (15.22%)		1,141.75 (17.3%)	128.93 (14.37%)
	GCSE A-C	1,381 (24.86%)	128 (25.3%)		1,844.99 (27.95%)	258.99 (28.87%)
	Other/None	1,183 (21.29%)	185 (36.56%)		1,471.73 (22.3%)	349.78 (38.99%)
Ethnicity	White	4,498 (68.27%)	412 (69.59%)	0%	5,580.25 (84.55%)	776.50 (86.55%)*
	Mixed	298 (4.52%)	30 (5.07%)		165.52 (2.51%)	23.70 (2.64%)
	Indian	439 (6.66%)	15 (2.53%)		146.52 (2.22%)	8.73 (0.97%)
	Pakistani	354 (5.37%)	38 (6.42%)		158.68 (2.4%)	21.96 (2.45%)
	Bangladeshi	289 (4.39%)	41 (6.93%)		76.53 (1.16%)	12.84 (1.43%)
	Black African	197 (2.99%)	28 (4.73%)		95.29 (1.44%)	26.65 (2.97%)
	Black Caribbean	278 (4.22%)	12 (2.03%)		162.33 (2.46%)	10.42 (1.16%)
	Other	236 (3.58%)	16 (2.7%)		214.77 (3.25%)	16.34 (1.82%)

* $p < 0.05$ from Meng and Rubin [41] Likelihood Ratio test.

Quantile Regression

The results of the quantile regressions are presented in **Error! Reference source not found.**. Except at the lowest quantiles of GHQ scores (which indicate good mental health), youth unemployment is

For peer review only - http://bmjopen.bmj.com/site/about/guidelines.xhtml

associated with worse mental health at age 25 (Column A). Associations are only partly attenuated controlling for adolescent mental health (Column B) or adolescent mental health and other background characteristics (Column C). Consistent with our hypothesis, associations are stronger at quantiles representing greater levels of poor mental health. For example, the predicted GHQ score at the 50th percentile among those with 6+ months unemployment is 0.75 (CI = -0.02, 1.57; b = 0.11) points greater than at the 50th percentile among those with less than 6 months unemployment. At the 90th percentiles, the difference is 3.68 (CI = 1.85, 5.67; b = 0.57) points. Further adjustment for current employment status partly attenuates these estimates (Column D), but associations remain substantial at highest quantiles. The results for models in Columns C and D are displayed graphically in Figure 1.

Table 2: Main Regression Results

Quantile	(A)	(B)	(C)	(D)	(E)	(F)
Q10	0<br>(-1, 1)	0<br>(-0.64, 0.57)	0.19<br>(-0.61, 1)	0.16<br>(-0.68, 0.98)	0.85<br>(-0.15, 1.81)	-0.47<br>(-1.61, 0.95)
Q20	0<br>(-1, 1)	0.32<br>(-0.31, 0.89)	0.47<br>(-0.25, 1.12)	0.41<br>(-0.3, 1.01)	0.77<br>(-0.12, 1.6)	0.29<br>(-1.06, 1.33)
Q30	1<br>(0, 2)	0.5<br>(-0.19, 1.21)	0.47<br>(-0.2, 1.18)	0.25<br>(-0.41, 0.91)	0.56<br>(-0.34, 1.52)	0.43<br>(-0.57, 1.41)
Q40	1<br>(1, 2)	0.67<br>(-0.07, 1.59)	0.56<br>(-0.18, 1.32)	0.21<br>(-0.53, 0.96)	0.56<br>(-0.37, 1.63)	0.46<br>(-0.55, 1.49)
Q50	1<br>(0, 2)	1.14<br>(0.37, 1.81)	0.75<br>(-0.02, 1.57)	0.26<br>(-0.6, 1.12)	0.77<br>(-0.31, 1.93)	0.61<br>(-0.59, 1.97)
Q60	2<br>(1, 3)	1.56<br>(0.59, 2.5)	1.13<br>(0.18, 2.33)	0.59<br>(-0.35, 1.78)	1.06<br>(-0.18, 2.62)	1.15<br>(-0.3, 3.05)
Q70	3<br>(2, 4)	2.45<br>(1.26, 3.96)	2.12<br>(0.78, 3.45)	1.61<br>(0.19, 2.85)	1.88<br>(0.01, 4.02)	2.19<br>(0.24, 3.88)
Q80	4<br>(3, 6)	3.88<br>(1.97, 6.04)	2.93<br>(1.21, 4.8)	2.24<br>(0.78, 3.94)	3.28<br>(0.69, 6.3)	2.33<br>(0.42, 4.36)
Q90	5<br>(3, 8)	6.07<br>(3.33, 7.54)	3.68<br>(1.85, 5.67)	2.58<br>(0.88, 4.27)	4.16<br>(1.2, 7.14)	2.35<br>(0.32, 4.35)
Observations	7,363	7,363	7,363	7,363	3,196	4,167
Imputations	28	28	28	28	28	28

Quantile regression of GHQ-12 Likert scores at age 25 on 6+ months unemployment experience between ages 18-20. (A) Bivariate association. (B) adjustment for GHQ-12 scores at ages 14/15 and 16/17. (C) Model B plus adjustment for disability and self-rated health, educational attainment, risk behaviours, attitude to school and bullying victimisation, IMD quintile, parental socio-economic class and education, gender, locus of control, and ethnicity. (D) Model C plus adjustment for current economic activity. (E) Model C estimated for males only. (F) Model C estimated for females only.

INSERT FIGURE 1 HERE

Predicted GHQ-12 scores according to youth unemployment experience derived from fully adjusted model (excluding current activity) in Column C are displayed in Figure 2. (Other covariates kept at the sample means.) Over 30% of those with 6+ months unemployment are predicted to have GHQ Likert scores above 15, 10% points more than those with less youth unemployment experience. For context,

one recent Swedish validation study found a cut-off of 11/12 points provides adequate sensitivity and specificity for detecting depression [32].

INSERT FIGURE 2 HERE

Figure 3 shows the results of the adjusted model not including current economic activity stratifying by gender. Stronger associations are again found at poorest levels of mental health, and effect sizes are similar in each gender. The results are also displayed in Columns E and F of Table 2 (male and female, respectively). Full regression results are displayed in the Supplementary Information.

INSERT FIGURE 3 HERE

Sensitivity Analysis

The results of the 20,000 models, representing a random selection of all possible combinations of the covariates, are displayed in Figure 4. Each line represents a single model run for each decile of GHQ-12 scores at age 25. Overlaid on the figure are the results of the main fully adjusted models, with and without further control for current economic status. The results are very consistent regardless of the set of control variables used.

INSERT FIGURE 4 HERE

DISCUSSION

Using a quantile regression approach, we find that, on average, individuals who were continuously unemployed for 6+ months between ages 18-20 have poorer mental health at age 25, but that there is substantial heterogeneity with associations being more pronounced at quantiles representing the worse mental health. Effect sizes at poorer levels of mental health are substantial and clinically significant: a higher proportion of those who were unemployed as youths exceed suggested thresholds for detecting depression with GHQ-12 Likert scores [32]. Associations remain after adjusting for adolescent mental and physical health, suggesting results may not be explained by health-related selection into unemployment, though there appears to be measurement error in the instruments we use. Associations were not fully attenuated when adjusting for current employment status and were robust to using different combinations of control variables.

Our results are consistent with previous studies which show that youth unemployment is related to worse mental health later in life [9–13] irrespective of later employment outcomes [12], but we extend the literature by showing that associations differ substantially across individuals. A natural next question is to identify individuals who appear to be most impacted by youth unemployment. To date, few studies have explored moderation of scarring effects. Lee et al. [9] find little evidence of differences by neighbourhood deprivation, while Bijlsma and colleagues [15] find evidence that scarring effects are stronger among males with low education. Other possible moderators which deserve further exploration are family socio-economic position and personality traits, such as locus of control. Locus of control has been linked to psychological adjustment following adversity [42] [though, also see 43] and also to active job-seeking behaviours among the unemployed [44]. Identifying vulnerable groups would have important implications for the targeting of policy interventions.

Another avenue for future research is to identify the mechanisms through which scarring effects may operate. Biljsma and colleagues [15] find evidence that the association between unemployment and later depression is partly mediated by income, but we know of no study that has assessed whether unemployment during adolescence alters neurobehavioural development. These pathways have different implications for designing policy to reduce scarring effects and potentially for identifying vulnerable groups. At present we are unable to say whether the heterogeneity observed reflects

For peer review only - http://bmjopen.bmj.com/site/about/guidelines.xhtml

individual differences in psychological resilience or propensities for unemployment to engender future socioeconomic adversity.

Our study has a number of limitations. We use observational data and as such, our results cannot be taken as indicating causality. Associations may reflect health-related selection into unemployment, though we adjust for adolescent mental and physical health and differences in GHQ scores were observed across most of the distribution – mental health-related selection into unemployment is arguably only a plausible explanation where psychological morbidity is severe enough to preclude job-finding or job-holding. Nevertheless, there are several other factors that are unobserved or imperfectly measured in our study, such as personality traits and human capital, which may explain associations. Controlling for current economic activity is likely to have induced collider bias. Estimates may have appeared more attenuated than was accurate.

Another limitation is that, by focusing on unemployment during ages 18-20, we exclude from the unemployed group those that only enter the labour market at a later age, i.e. those that went to university. Previously research suggests scarring effects may be smaller among this group [15]. Our use of the GHQ as a measure of mental health could also have biased results as items inquire about current symptoms vis-à-vis typical experience. This may not appropriately capture chronic psychological ill health, though a recent validation study finds that the GHQ displays good sensitivity for detecting depression [32].

Our results may be specific to the cohort we studied. The period in which we measure youth unemployment overlapped with the Global Financial Crisis, following which youth unemployment rates rose worldwide [1]. The long-term consequences of unemployment may be smaller following a recession as prospective employers may look on periods of unemployment less unfavourably [45], and so social chains of risk may be weaker. Further, outcomes for those that do not become unemployed - who we use to measure long-term effects - may simultaneously get worse as labour markets get weaker [46].

Finally, the results may be specific to the age we studied. Participants were early in their labour market careers when we measured mental health outcomes. Differences between the youth unemployed and their peers may grow through time (e.g. following promotions) or become progressively more important if, for instance, individuals are less able to rely on financial help from their families as they age. Alternatively, differences could dissipate if individuals are able to recover from early labour market adversity and it is "chains of risk" that explain our results. A useful extension to this study would be to repeat the analysis at later ages and in other countries with different labour market institutions.

Figures:

Figure 1: Association between 6+ months youth unemployment between ages 18-20 and GHQ-12 Likert scores at age 25, by decile of GHQ-12. (a) adjusted for adolescent mental health, disability and self-rated health, educational attainment, risk behaviours, attitude to school and bullying victimisation, IMD quintile, parental socio-economic class and education, gender, locus of control, and ethnicity. (b) additionally adjusted for current status.

Figure 2: Predicted age 25 GHQ-12 Likert scores by youth unemployment experience, by decile of GHQ-12. Models include adjustment for adolescent mental health, disability and self-rated health, educational attainment, risk behaviours, attitude to school and bullying victimisation, IMD quintile, parental socio-economic class and education, gender, locus of control, and ethnicity. Predicted values derived used sample means for covariates.

Figure 3: Association between 6+ months youth unemployment between ages 18-20 and GHQ-12 Likert scores at age 25, by gender and decile of GHQ-12. Adjusted for adolescent mental health, disability and self-rated health, educational attainment, risk behaviours, attitude to school and bullying

For peer review only - http://bmjopen.bmj.com/site/about/guidelines.xhtml

victimisation, IMD quintile, parental socio-economic class and education, gender, locus of control, and ethnicity.

Figure 4: Association between better 6+ months youth unemployment between ages 18-20 and GHQ-12 Likert scores at age 25, by decile of GHQ-12. 20,000 models drawn from all possible combinations of covariates. Black lines are results of fully adjusted models, including (dashed) or not including (solid) further adjustment for current economic activity.

Funding: This work was funded by the Economic and Social Research Council through the UCL, Bloomsbury and East London Doctoral Training Partnership (ES/P000592/1). The funding was awarded to Liam Wright.

Conflicts of Interest: None declared.

Acknowledgements: None.

Author Contributions: All authors contributed to the design of the study. LW carried out the analysis and wrote the manuscript. JH and SJ provided detailed comments on the manuscript.

Data Statement: The code to replicate this analysis is available at https://osf.io/3gwap/. Next Steps data are publicly available through the UK Data Service [30].

For peer review only

REFERENCES

1 Bell DNF, Blanchflower DG. US AND UK LABOUR MARKETS BEFORE AND DURING THE COVID-19 CRASH. *NIER* 2020;**252**:R52–69. doi:10.1017/nie.2020.14

2 Paul KI, Moser K. Unemployment impairs mental health: Meta-analyses. *Journal of Vocational Behavior* 2009;**74**:264–282. doi:10.1016/j.jvb.2009.01.001

3 Gathergood J. An Instrumental Variable Approach To Unemployment, Psychological Health and Social Norm Effects. *Health Economics* 2013;**22**:643–654. doi:10.1002/hec.2831

4 Schmitz H. Why are the unemployed in worse health? The causal effect of unemployment on health. *Labour Economics* 2011;**18**:71–8. doi:10.1016/j.labeco.2010.08.005

5 Creed PA, Bartrum D. Explanations for deteriorating wellbeing in unemployed people: Specific unemployment theories and beyond. In: *Unemployment and health – International & interdisciplinary perspectives*. 2006.

6 Jahoda M. *Employment and Unemployment: A Social-Psychological Analysis*. Cambridge: : Cambridge University Press 1982.

7 Blustein DL, Duffy R, Ferreira JA, *et al.* Unemployment in the time of COVID-19: A research agenda. *Journal of Vocational Behavior* 2020;**119**:103436. doi:10.1016/j.jvb.2020.103436

8 Clark AE, Georgellis Y, Sanfey P. Scarring: The psychological impact of past unemployment. *Economica* 2001;**68**:221–241. doi:10.1111/1468-0335.00243

9 Lee JO, Jones TM, Yoon Y, *et al.* Young Adult Unemployment and Later Depression and Anxiety: Does Childhood Neighborhood Matter? *Journal of Youth and Adolescence* 2019;**48**:30–42. doi:10.1007/s10964-018-0957-8

10 Bell DNF, Blanchflower DG. Young people and the great recession. *Oxford Review of Economic Policy* 2011;**27**:241–267. doi:10.1093/oxrep/grr011

11 Wheaton B, Reid S. The role of timing vs. duration in the cumulative work history effects of job exits and nonemployment on women's mental health. *Advances in Life Course Research* 2008;**13**:195–232. doi:10.1016/S1040-2608(08)00008-7

12 Strandh M, Winefield A, Nilsson K, *et al.* Unemployment and mental health scarring during the life course. *European Journal of Public Health* 2014;**24**:440–445. doi:10.1093/eurpub/cku005

13 Thern E, de Munter J, Hemmingsson T, *et al.* Long-term effects of youth unemployment on mental health: Does an economic crisis make a difference? *Journal of Epidemiology and Community Health* 2017;**71**:344–349. doi:10.1136/jech-2016-208012

14 Ben-Shlomo Y, Kuh D. A life course approach to chronic disease epidemiology: conceptual models, empirical challenges and interdisciplinary perspectives. *International Journal of Epidemiology* 2002;**31**:285–293. doi:10.1093/intjepid/31.2.285

15 Bijlsma MJ, Tarkiainen L, Myrskylä M, *et al.* Unemployment and subsequent depression: A mediation analysis using the parametric G-formula. *Social Science and Medicine* 2017;**194**:142–150. doi:10.1016/j.socscimed.2017.10.011

16 Schmillen A, Umkehrer M. The scars of youth: Effects of early-career unemployment on future unemployment experience. *International Labour Review* 2017;**156**:465–94. doi:10.1111/ilr.12079

For peer review only - http://bmjopen.bmj.com/site/about/guidelines.xhtml

17 Fuhrmann D, Knoll LJ, Blakemore SJ. Adolescence as a Sensitive Period of Brain Development. *Trends in Cognitive Sciences* 2015;**19**:558–566. doi:10.1016/j.tics.2015.07.008

18 Koenker R, Bassett G. Regression Quantiles. *Econometrica* 1978;**46**:33. doi:10.2307/1913643

19 Binder M, Coad A. Heterogeneity in the Relationship Between Unemployment and Subjective Wellbeing: A Quantile Approach. *Economica* 2015;**82**:865–891. doi:10.1111/ecca.12150

20 Binder M, Coad A. Unemployment impacts differently on the extremes of the distribution of a comprehensive well-being measure. *Applied Economics Letters* 2015;**22**:619–627. doi:10.1080/13504851.2014.962219

21 Graham C, Nikolova M. Bentham or Aristotle in the Development Process? An Empirical Investigation of Capabilities and Subjective Well-Being. *World Development* 2015;**68**:163–79. doi:10.1016/j.worlddev.2014.11.018

22 Clark AE, Lepinteur A. The causes and consequences of early-adult unemployment: Evidence from cohort data. *Journal of Economic Behavior & Organization* 2019;**166**:107–24. doi:10.1016/j.jebo.2019.08.020

23 Georgellis Y, Gregoriou A, Healy J, *et al.* Unemployment and life satisfaction: a non-linear adaptation process. *Int J of Manpower* 2008;**29**:668–80. doi:10.1108/01437720810908956

24 Galatzer-Levy IR, Bonanno GA, Mancini AD. From marianthal to latent growth mixture modeling: A return to the exploration of individual differences in response to unemployment. *Journal of Neuroscience, Psychology, and Economics* 2010;**3**:116–25. doi:10.1037/a0020077

25 Bernardi F, Grätz M. Making Up for an Unlucky Month of Birth in School: Causal Evidence on the Compensatory Advantage of Family Background in England. *SocScience* 2015;**2**:235–51. doi:10.15195/v2.a12

26 Galatzer-Levy IR, Huang SH, Bonanno GA. Trajectories of resilience and dysfunction following potential trauma: A review and statistical evaluation. *Clinical Psychology Review* 2018;**63**:41–55. doi:10.1016/j.cpr.2018.05.008

27 Etilé F, Frijters P, Johson DW, *et al.* Modelling Heterogeneity in the Resilience to Major Socioeconomic Life Events. Paris School of Economics 2017. https://halshs.archives-ouvertes.fr/halshs-01485989/document

28 Nandi A, Tracy M, Beard JR, *et al.* Patterns and Predictors of Trajectories of Depression after an Urban Disaster. *Annals of Epidemiology* 2009;**19**:761–70. doi:10.1016/j.annepidem.2009.06.005

29 Hammarström A, Gustafsson PE, Janlert U, *et al.* It's no surprise! Men are not hit more than women by the health consequences of unemployment in the Northern Swedish Cohort. *Scandinavian Journal of Public Health* 2011;**39**:187–193. doi:10.1177/1403494810394906

30 Centre for Longitudinal Studies. *Next Steps: Sweeps 1-8, 2004-2016 (SN: 5545)*. 14th ed. UK Data Service 2018. http://doi.org/10.5255/UKDA-SN-5545-6

31 Goldberg DP, Hillier VF. A scaled version of the General Health Questionnaire. *Psychological Medicine* 1979;**9**:139–145. doi:10.1017/S0033291700021644

32 Lundin A, Hallgren M, Theobald H, *et al.* Validity of the 12-item version of the General Health Questionnaire in detecting depression in the general population. *Public Health* 2016;**136**:66–74. doi:10.1016/j.puhe.2016.03.005

For peer review only - http://bmjopen.bmj.com/site/about/guidelines.xhtml

33 Morrell S, Taylor R, Quine S, *et al.* A cohort study of unemployment as a cause of psychological disturbance in Australian youth. *Social Science and Medicine* 1994;**38**:1553–1564. doi:10.1016/0277-9536(94)90117-1

34 Ministry of Housing, Communities and Local Government. Index of Multiple Deprivation 2004. 2014.https://data.gov.uk/dataset/59599787-bd50-4500-a409-fc586260dbbd/index-of-multiple-deprivation-2004 (accessed 25 Oct 2019).

35 Carpenter JR, Kenward MG. *Multiple imputation and its application*. 1st ed. Chichester, West Sussex, UK: : John Wiley & Sons 2013.

36 VanderWeele TJ. Principles of confounder selection. *Eur J Epidemiol* 2019;**34**:211–9. doi:10.1007/s10654-019-00494-6

37 van Buuren S, Groothuis-Oudshoorn K. mice: Multivariate Imputation by Chained Equations in R. *J Stat Soft* 2011;**45**. doi:10.18637/jss.v045.i03

38 R Core Team. *R: A Language and Environment for Statistical Computing*. Vienna, Austria: : R Foundation for Statistical Computing 2020. https://www.R-project.org/

39 Egan M, Daly M, Delaney L. Adolescent psychological distress, unemployment, and the Great Recession: Evidence from the National Longitudinal Study of Youth 1997. *Social Science and Medicine* 2016;**156**:98–105. doi:10.1016/j.socscimed.2016.03.013

40 Caspi A, Wright BRE, Moffitt TE, *et al.* Early Failure in the Labor Market: Childhood and Adolescent Predictors of Unemployment in the Transition to Adulthood. *American Sociological Review* 1998;**63**:424. doi:10.2307/2657557

41 Meng X-L, Rubin DB. Performing likelihood ratio tests with multiply-imputed data sets. *Biometrika* 1992;**79**:103–11. doi:10.1093/biomet/79.1.103

42 Kammeyer-Mueller JD, Judge TA, Scott BA. The Role of Core Self-Evaluations in the Coping Process. *Journal of Applied Psychology* 2009;**94**:177–195. doi:10.1037/a0013214

43 Buddelmeyer H, Powdthavee N. Can having internal locus of control insure against negative shocks? Psychological evidence from panel data. *Journal of Economic Behavior & Organization* 2016;**122**:88–109. doi:10.1016/j.jebo.2015.11.014

44 Cobb-Clark DA. Locus of control and the labor market. *IZA Journal of Labor Economics* 2015;**4**:3. doi:10.1186/s40172-014-0017-x

45 Kroft K, Lange F, Notowidigdo MJ. Duration dependence and labor market conditions: Evidence from a field experiment. *Quarterly Journal of Economics* 2013;**128**. doi:10.1093/qje/qjt015

46 Green F. Unpacking the misery multiplier: How employability modifies the impacts of unemployment and job insecurity on life satisfaction and mental health. *Journal of Health Economics* 2011;**30**:265–276. doi:10.1016/j.jhealeco.2010.12.005

For peer review only - http://bmjopen.bmj.com/site/about/guidelines.xhtml

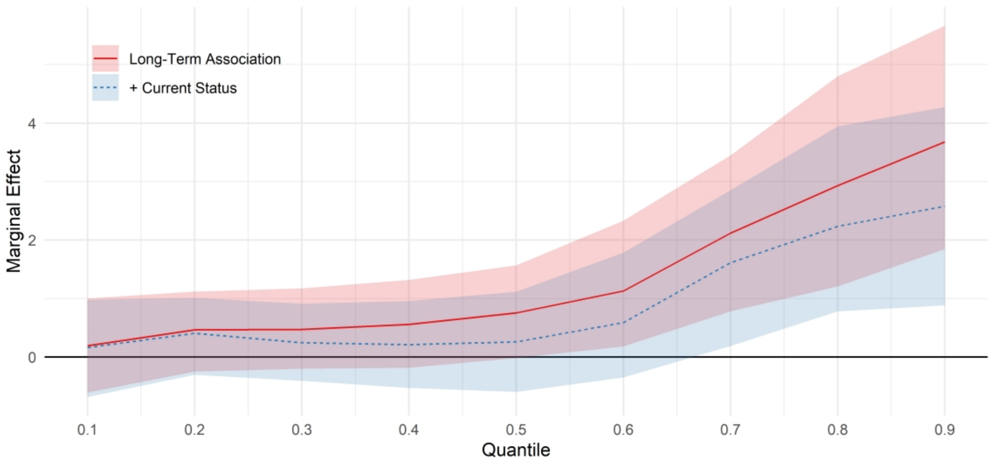

Figure 1: Association between 6+ months youth unemployment between ages 18-20 and GHQ-12 Likert scores at age 25, by decile of GHQ-12. (a) adjusted for adolescent mental health, disability and self-rated health, educational attainment, risk behaviours, attitude to school and bullying victimisation, IMD quintile, parental socio-economic class and education, gender, locus of control, and ethnicity. (b) additionally adjusted for current status.

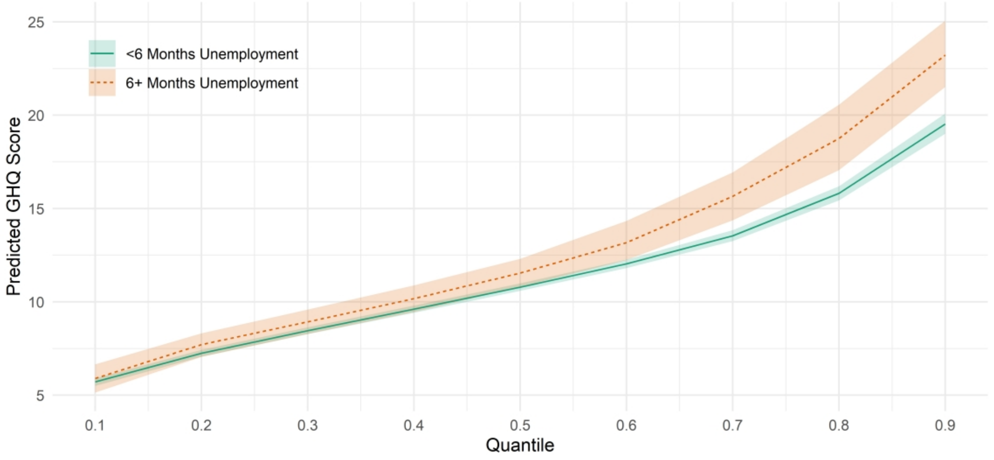

Figure 2: Predicted age 25 GHQ-12 Likert scores by youth unemployment experience, by decile of GHQ-12. Models include adjustment for adolescent mental health, disability and self-rated health, educational attainment, risk behaviours, attitude to school and bullying victimisation, IMD quintile, parental socio-economic class and education, gender, locus of control, and ethnicity. Predicted values derived used sample means for covariates.

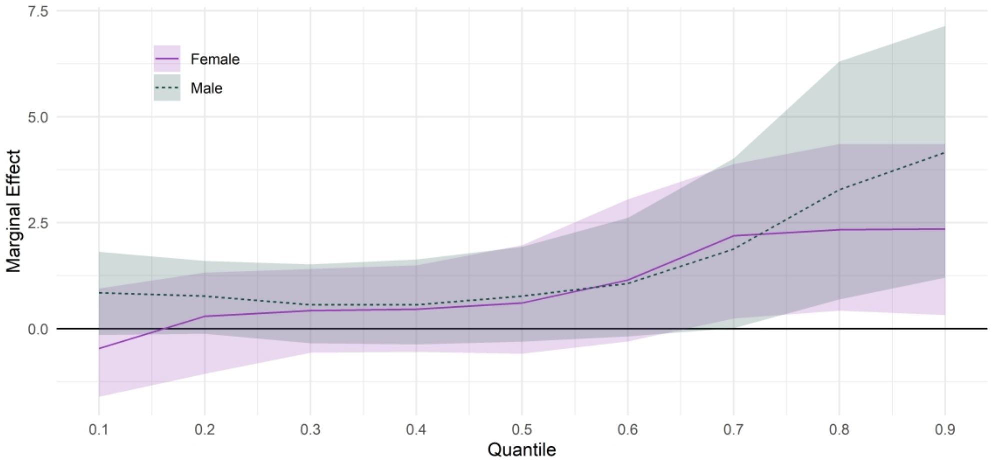

Figure 3: Association between 6+ months youth unemployment between ages 18-20 and GHQ-12 Likert scores at age 25, by gender and decile of GHQ-12. Adjusted for adolescent mental health, disability and self-rated health, educational attainment, risk behaviours, attitude to school and bullying victimisation, IMD quintile, parental socio-economic class and education, gender, locus of control, and ethnicity.

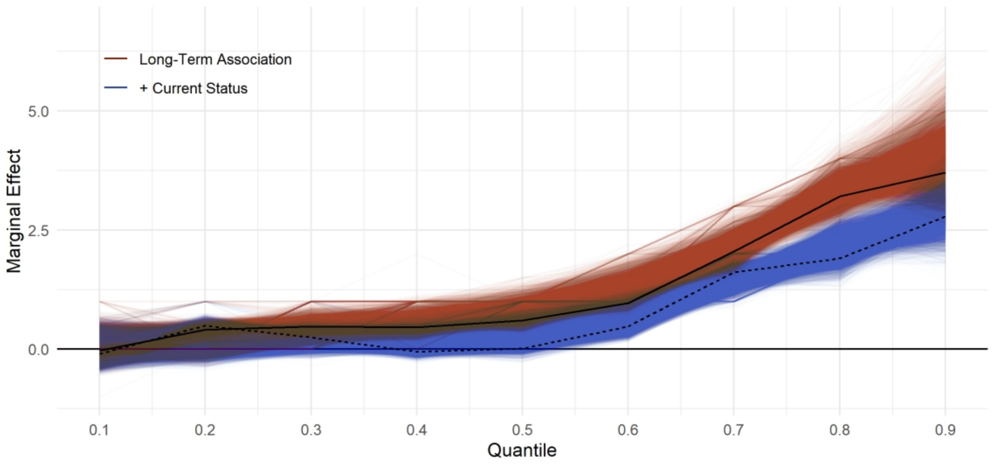

Figure 4: Association between better 6+ months youth unemployment between ages 18-20 and GHQ-12 Likert scores at age 25, by decile of GHQ-12. 20,000 models drawn from all possible combinations of covariates. Black lines are results of fully adjusted models, including (dashed) or not including (solid) further adjustment for current economic activity.

Supplementary Information

Methods

GHQ-12 Likert Scores and Chronic Depression

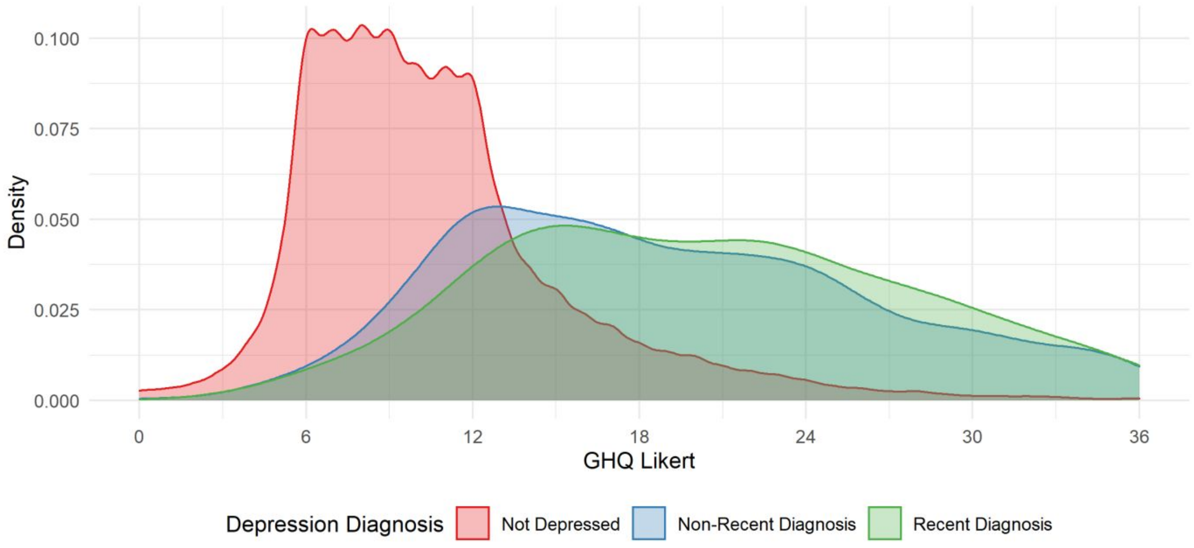

Figure S1: GHQ Likert Scores by recency of depression diagnosis. Recent diagnosis refers to diagnosis in prior two years. Non-recent diagnosis refers to diagnosis more than two years ago. Data drawn from Wave 1 of the United Kingdom Household Longitudinal Study, a representative panel survey of households in the UK.

Locus of Control

Locus of control (LOC) was measured at age 14/15 with participants asked for their level of agreement (strongly agree, agree, disagree, strongly disagree, don't know) with six separate statements. Three of the statements were worded to reflect an internal LOC ("if someone is not a success in life, it is usually their own fault"; "I can pretty much decide what will happen in my life"; "if you work hard at something you'll usually succeed") and three worded to reflect an external LOC ("even if I do well at school, I'll have a hard time getting the right type of job", "people like me don't have much of a chance in life", "how well you get on in this world is mostly a matter of luck"). I place responses onto a five-point scale, centred around "don't know", with external-worded items reverse coded so higher scores indicate less external LOC.

There is no agreed way of combining items to operationalise LOC, both in the Next Steps data specifically and in other large-scale survey datasets which measure LOC (Buddelmeyer & Powdthavee, 2016; Caliendo et al., 2015; Cobb-Clark et al., 2014; Piatek & Pinger, 2016). Researchers using Next Steps data have used different subsets of the LOC items (cf. Crawford et al., 2011; Mendolia & Walker, 2015; Ng-Knight & Schoon, 2017; Wijedasa, 2017),[1] and combined them in several ways, including summing Likert responses (Crawford et al., 2011; Ng-Knight & Schoon, 2017) or extracting latent LOC scores using principal component analysis (Wijedasa, 2017), EFA (Mendolia & Walker, 2015), or CFA (Gladwell et al., 2016).

[1] Wijedasa (2017) includes another item, "[w]orking hard at school now will help me get on later in life", their LOC measure, while Crawford et al. (2011) include this plus another item, "[d]oing well at school means a lot to me". I do not include these items as they arguably capture opinions about the value of secondary education rather than LOC. Ng-Knight & Schoon (2017) and Gladwell et al. (2016) use the three internal-worded items introduced above, only.

Though internal and external-worded items were originally conceptualized as tapping opposite ends of a single spectrum (Rotter, 1966), a number of exploratory factor analyses have found internal and external worded items load onto separate factors (Caliendo et al., 2015; Cobb-Clark & Schurer, 2013; Piatek & Pinger, 2016), including an analysis of Next Steps (Mendolia & Walker, 2015). In the Next Steps data, a CFA with two correlated latent factors has superior fit statistics to a single factor CFA model (Table). The correlation between the latent factors is just 0.4.

Table 1: Fit Statistics from Confirmatory Factor Analyses of Locus of Control Items

Model	RMSEA (95% CI)	CFI
One Factor	0.073 (0.069, 0.078)	0.818
Two Factor	0.044 (0.039, 0.05)	0.941
Hankins (2008)	0.034 (0.028, 0.04)	0.975

However, an alternative explanation is that responses reflect method effects. A single factor CFA with internal-worded items allowed to covary (i.e akin to Hankins' (2008) GHQ model) has superior fit statistics to the two-factor solution (Table 1). Given the possibility of method effects, the widespread use of only the first factor from EFA (Cobb-Clark, 2015), and the appeal of understanding locus of control as a singular construct (Rotter, 1975), we measure LOC by extracting a single factor using the Hankins (2008) CFA model (DWLS estimator).[2]

Note, the items are not drawn from a validated measure of LOC and have poor reliability (Cronbach's α = 0.40). However, the items have been used in published studies before (Chowdry et al., 2011; Crawford et al., 2011; Department for Education et al., 2012; Gladwell et al., 2016; Mendolia & Walker, 2014a, 2014b, 2015; Ng-Knight & Schoon, 2017; Wijedasa, 2017). For instance, Mendolia & Walker (2015) find that participants with external LOC are more likely to be NEET by age 20, while Ng-Knight and Schoon (2017) find evidence that internal LOC moderates the association between low SEP and youth worklessness. The LOC factor we extract is also associated with several variables it should be expected to, including GHQ scores at age 25 and youth unemployment experience.

[2] An alternate model which allows covariance among external-worded items instead has poorer fit statistics.

For peer review only

Results

Table 2: Quantile Regression Results. Bivariate association (Column A, Table 2 in main paper)

Variable	Q10	Q20	Q30	Q40	Q50	Q60	Q70	Q80	Q90
6+ Months Unemployment	0	0	1	1	1	2	3	4	5
	(-1, 1)	(-1, 1)	(0, 2)	(1, 2)	(0, 2)	(1, 3)	(2, 4)	(3, 6)	(3, 8)
Constant	6	7	8	9	11	12	13	16	20
	(5, 6)	(7, 7)	(8, 8)	(9, 10)	(10, 11)	(12, 12)	(13, 13)	(15, 16)	(20, 21)
Observations	7,363	7,363	7,363	7,363	7,363	7,363	7,363	7,363	7,363
Imputations	28	28	28	28	28	28	28	28	28

For peer review only

Table 3: Quantile Regression Results. Adjustment for adolescent mental health (Column B, Table 2 in main paper)

Variable	Q10	Q20	Q30	Q40	Q50	Q60	Q70	Q80	Q90
6+ Months Unemployment	0<br>(-0.64, 0.57)	0.32<br>(-0.31, 0.89)	0.5<br>(-0.19, 1.21)	0.67<br>(-0.07, 1.59)	1.14<br>(0.37, 1.81)	1.56<br>(0.59, 2.5)	2.45<br>(1.26, 3.96)	3.88<br>(1.97, 6.04)	6.07<br>(3.33, 7.54)
GHQ-12 @ Age 14/15	0.14<br>(0.03, 0.22)	0.14<br>(0.08, 0.23)	0.16<br>(0.07, 0.25)	0.19<br>(0.1, 0.28)	0.26<br>(0.18, 0.35)	0.33<br>(0.23, 0.44)	0.43<br>(0.3, 0.58)	0.57<br>(0.38, 0.81)	0.68<br>(0.5, 0.92)
GHQ-12 @ Age 16/17	0.13<br>(0.09, 0.18)	0.19<br>(0.14, 0.22)	0.21<br>(0.18, 0.25)	0.23<br>(0.19, 0.26)	0.24<br>(0.2, 0.28)	0.28<br>(0.23, 0.33)	0.32<br>(0.25, 0.38)	0.38<br>(0.31, 0.46)	0.42<br>(0.33, 0.53)
Constant	4.1<br>(3.6, 4.5)	5.04<br>(4.73, 5.38)	5.87<br>(5.56, 6.23)	6.85<br>(6.5, 7.2)	7.75<br>(7.34, 8.2)	8.5<br>(8, 9)	9.33<br>(8.81, 9.83)	10.53<br>(9.94, 11.13)	13.62<br>(12.77, 14.71)
Observations	7,363	7,363	7,363	7,363	7,363	7,363	7,363	7,363	7,363
Imputations	28	28	28	28	28	28	28	28	28

Table 4: Quantile Regression Results. Fully adjusted model excluding current economic activity (Column C, Table 2 in main paper)

	Variable	Q10	Q20	Q30	Q40	Q50	Q60	Q70	Q80	Q90
	6+ Months Unemployment	0.19 (-0.61, 1)	0.47 (-0.25, 1.12)	0.47 (-0.2, 1.18)	0.56 (-0.18, 1.32)	0.75 (-0.02, 1.57)	1.13 (0.18, 2.33)	2.12 (0.78, 3.45)	2.93 (1.21, 4.8)	3.68 (1.85, 5.67)
	Female	0.51 (0.16, 0.87)	0.56 (0.25, 0.88)	0.48 (0.15, 0.81)	0.34 (0, 0.7)	0.29 (-0.07, 0.63)	0.29 (-0.09, 0.68)	0.22 (-0.28, 0.68)	0.02 (-0.63, 0.65)	-0.69 (-1.64, 0.19)
	IMD	0 (-0.01, 0.01)	0 (-0.01, 0.01)	0 (-0.01, 0.01)	0 (-0.01, 0.01)	0 (-0.01, 0.02)	0 (-0.01, 0.02)	0 (-0.01, 0.02)	0.01 (-0.01, 0.04)	0.03 (0, 0.06)
	Locus of Control	-0.22 (-0.4, -0.03)	-0.13 (-0.34, 0.06)	-0.14 (-0.34, 0.06)	-0.16 (-0.37, 0.05)	-0.12 (-0.34, 0.1)	-0.11 (-0.35, 0.13)	0 (-0.28, 0.29)	0.06 (-0.35, 0.45)	0.01 (-0.53, 0.55)
	GHQ-12 @ Age 14/15	0.08 (-0.02, 0.18)	0.12 (0.03, 0.19)	0.09 (0.01, 0.18)	0.11 (0.01, 0.2)	0.17 (0.07, 0.27)	0.25 (0.14, 0.36)	0.3 (0.17, 0.44)	0.38 (0.19, 0.59)	0.48 (0.24, 0.71)
	GHQ-12 @ Age 16/17	0.12 (0.08, 0.17)	0.16 (0.12, 0.2)	0.2 (0.16, 0.23)	0.21 (0.17, 0.24)	0.22 (0.18, 0.27)	0.27 (0.21, 0.32)	0.3 (0.24, 0.36)	0.35 (0.27, 0.42)	0.4 (0.31, 0.48)
Self-Rated Health @ Age 14/15	Fairly Good	0.18 (-0.22, 0.6)	0.26 (-0.08, 0.59)	0.34 (-0.02, 0.71)	0.4 (0.02, 0.77)	0.36 (-0.04, 0.77)	0.31 (-0.12, 0.74)	0.3 (-0.22, 0.82)	0.51 (-0.16, 1.21)	1 (0.02, 2)
	Not Very Good	0.48 (-0.82, 1.69)	0.55 (-0.66, 1.72)	0.61 (-0.67, 2.18)	1.11 (-0.65, 2.26)	0.9 (-0.38, 2.07)	0.78 (-0.55, 2.64)	1.22 (-0.68, 3.9)	1.85 (-0.83, 4.98)	1.87 (-0.93, 5.39)
	Not Good at All	0.23 (-2.03, 1.86)	-0.29 (-1.56, 1.52)	-0.62 (-2, 1.84)	-0.49 (-2.63, 2.76)	-0.3 (-3.11, 3.6)	-0.26 (-3.14, 3.94)	0.21 (-3.55, 5.45)	-0.21 (-3.92, 6.79)	1.37 (-4.94, 6.37)
Self-Rated Health @ Age 16/17	Fairly Good	0.25 (-0.17, 0.68)	0.16 (-0.18, 0.51)	0.14 (-0.23, 0.5)	0.22 (-0.17, 0.6)	0.16 (-0.24, 0.55)	0.08 (-0.36, 0.51)	0.17 (-0.37, 0.7)	0.21 (-0.5, 0.95)	0.13 (-0.91, 1.19)

	Variable	Q10	Q20	Q30	Q40	Q50	Q60	Q70	Q80	Q90
	Not Very Good	-0.07 (-0.95, 0.74)	-0.1 (-1.14, 0.86)	0.27 (-0.73, 1.3)	0.57 (-0.34, 1.45)	0.56 (-0.34, 1.43)	0.48 (-0.49, 1.75)	0.61 (-0.67, 2.18)	0.56 (-1.13, 2.25)	-0.06 (-2, 2.01)
	Not Good at All	-0.83 (-2.97, 1.87)	0.13 (-2.49, 1.95)	0.2 (-2.1, 2.07)	0.31 (-1.81, 2.32)	0.77 (-1.72, 2.75)	0.48 (-1.42, 4.84)	1.6 (-1.57, 5.51)	1.48 (-2.03, 5.28)	-0.34 (-3.61, 3.43)
Disabled	Yes, school not affected	0.09 (-0.58, 0.67)	0.08 (-0.45, 0.57)	-0.09 (-0.6, 0.42)	-0.12 (-0.73, 0.6)	0.08 (-0.58, 0.71)	0.27 (-0.46, 1.2)	0.65 (-0.26, 1.53)	0.8 (-0.36, 2.28)	1.47 (-0.46, 3.35)
	Yes, school affected	0.35 (-0.71, 1.3)	0.36 (-0.49, 1.08)	0.3 (-0.48, 1.02)	0.32 (-0.51, 1.23)	0.55 (-0.29, 1.33)	0.62 (-0.29, 1.57)	0.78 (-0.47, 2.45)	1.35 (-0.34, 3.71)	2.2 (-0.19, 5)
	Risk Behaviours	-0.04 (-0.19, 0.07)	-0.1 (-0.2, 0.02)	-0.11 (-0.25, 0.05)	-0.06 (-0.22, 0.09)	-0.03 (-0.18, 0.13)	0 (-0.18, 0.19)	0.02 (-0.19, 0.23)	0.03 (-0.26, 0.38)	0.05 (-0.34, 0.42)
	Attitude to School	-0.02 (-0.05, 0.01)	-0.01 (-0.04, 0.01)	-0.02 (-0.05, 0.01)	-0.02 (-0.05, 0.01)	-0.02 (-0.05, 0.01)	-0.02 (-0.05, 0.01)	-0.03 (-0.07, 0.01)	-0.05 (-0.11, 0.01)	-0.06 (-0.13, 0.02)
	# Waves Bullied, 1-3	0.09 (-0.08, 0.26)	0.12 (-0.02, 0.26)	0.18 (0.02, 0.34)	0.24 (0.09, 0.4)	0.29 (0.12, 0.45)	0.34 (0.17, 0.52)	0.41 (0.18, 0.64)	0.58 (0.24, 0.93)	0.73 (0.3, 1.19)
Qualifications	NVQ 4	0.5 (-0.05, 1.06)	0.37 (-0.05, 0.81)	0.45 (-0.02, 0.91)	0.3 (-0.18, 0.8)	0.27 (-0.25, 0.77)	0.33 (-0.21, 0.87)	0.39 (-0.26, 1.05)	0.36 (-0.62, 1.26)	0.27 (-1.28, 1.66)
	NVQ 3	0.53 (-0.08, 1.13)	0.38 (-0.04, 0.83)	0.36 (-0.11, 0.84)	0.31 (-0.23, 0.86)	0.37 (-0.17, 0.89)	0.43 (-0.16, 1)	0.44 (-0.23, 1.08)	0.32 (-0.74, 1.35)	0.23 (-1.32, 1.68)
	NVQ 2	0.21 (-0.38, 0.83)	0.23 (-0.22, 0.68)	0.24 (-0.26, 0.73)	0.09 (-0.44, 0.64)	-0.03 (-0.56, 0.51)	-0.07 (-0.69, 0.53)	-0.12 (-0.87, 0.62)	-0.58 (-1.66, 0.43)	-1.3 (-2.89, 0.15)

	Variable	Q10	Q20	Q30	Q40	Q50	Q60	Q70	Q80	Q90
	NVQ 1	-0.56 (-1.51, 0.28)	-0.45 (-1.15, 0.29)	-0.32 (-1.06, 0.42)	-0.35 (-1.14, 0.45)	-0.16 (-0.94, 0.66)	0.11 (-0.74, 0.98)	0.21 (-0.86, 1.44)	0.64 (-0.94, 2.25)	0.77 (-1.42, 3)
	No/Other Qual	-0.45 (-1.48, 0.51)	-0.24 (-1.05, 0.55)	-0.18 (-0.97, 0.66)	-0.18 (-1.09, 0.72)	-0.09 (-1.01, 0.86)	0.28 (-0.75, 1.29)	0.65 (-0.63, 2.21)	0.97 (-0.94, 3.01)	0.04 (-2.22, 2.43)
Parental NS-SEC	Intermediate	0.15 (-0.39, 0.65)	0.08 (-0.34, 0.49)	0.02 (-0.38, 0.42)	-0.06 (-0.49, 0.38)	-0.12 (-0.62, 0.39)	-0.11 (-0.64, 0.45)	-0.03 (-0.67, 0.62)	0.26 (-0.59, 1.15)	0.47 (-0.89, 1.77)
	Routine	0.29 (-0.18, 0.76)	0.24 (-0.15, 0.64)	0.33 (-0.09, 0.74)	0.37 (-0.08, 0.82)	0.31 (-0.16, 0.76)	0.23 (-0.3, 0.77)	0.33 (-0.28, 0.95)	0.67 (-0.15, 1.46)	0.79 (-0.57, 2.06)
	LTU	-0.23 (-1.56, 0.95)	-0.26 (-1.33, 0.74)	-0.2 (-1.22, 0.87)	-0.03 (-1.2, 1.23)	0.1 (-1.14, 1.34)	0.39 (-0.98, 1.96)	1.06 (-0.6, 2.82)	1.47 (-0.6, 3.83)	1.97 (-0.88, 4.93)
Parental Education	Other HE	-0.27 (-0.85, 0.29)	-0.09 (-0.56, 0.34)	-0.21 (-0.75, 0.38)	0.01 (-0.54, 0.54)	0.06 (-0.49, 0.61)	0.1 (-0.51, 0.69)	0.13 (-0.61, 0.85)	0.02 (-0.9, 1.01)	0.29 (-1.01, 1.66)
	A-Level	-0.26 (-0.88, 0.31)	-0.19 (-0.68, 0.27)	-0.37 (-0.88, 0.16)	-0.28 (-0.83, 0.27)	-0.22 (-0.82, 0.38)	-0.12 (-0.76, 0.51)	-0.28 (-1, 0.46)	-0.65 (-1.6, 0.36)	-0.49 (-1.93, 1.13)
	GCSE A-C	-0.08 (-0.67, 0.48)	0 (-0.47, 0.48)	-0.11 (-0.59, 0.39)	-0.17 (-0.66, 0.32)	-0.2 (-0.76, 0.39)	-0.07 (-0.69, 0.55)	-0.12 (-0.82, 0.59)	-0.5 (-1.45, 0.52)	-0.59 (-2.06, 0.9)
	Other/None	-0.43 (-1.1, 0.28)	-0.01 (-0.61, 0.59)	-0.03 (-0.67, 0.61)	0.11 (-0.55, 0.81)	0.39 (-0.31, 1.09)	0.51 (-0.28, 1.31)	0.51 (-0.51, 1.5)	0.44 (-0.82, 1.78)	1.33 (-0.62, 3.29)
Ethnicity	Mixed	-0.69 (-1.77, 0.17)	-0.67 (-1.55, 0.14)	-0.63 (-1.56, 0.17)	-0.73 (-1.59, 0.02)	-0.97 (-1.78, 0.02)	-0.82 (-1.93, 0.45)	-0.3 (-1.79, 1.73)	0.43 (-1.41, 3.11)	1.54 (-1.48, 5.04)

For peer review only - http://bmjopen.bmj.com/site/about/guidelines.xhtml

Variable	Q10	Q20	Q30	Q40	Q50	Q60	Q70	Q80	Q90
Indian	-1.2 (-1.95, -0.27)	-0.81 (-1.43, -0.23)	-0.83 (-1.5, -0.03)	-0.6 (-1.42, 0.17)	-0.63 (-1.34, 0.12)	-0.47 (-1.33, 0.39)	-0.38 (-1.35, 0.88)	0.1 (-1.48, 1.68)	0.09 (-1.96, 3.02)
Pakistani	-0.68 (-1.57, 0.18)	-0.46 (-1.24, 0.21)	-0.63 (-1.29, 0.05)	-0.9 (-1.68, -0.15)	-1.2 (-1.98, -0.39)	-1.17 (-1.98, -0.24)	-0.95 (-2.11, 0.23)	-1.24 (-2.6, 0.17)	-1.95 (-3.97, 0.46)
Bangladeshi	-1.69 (-2.94, -0.31)	-1.23 (-2.69, 0)	-0.75 (-1.94, 0.13)	-0.82 (-1.81, 0.15)	-1.07 (-2.11, 0.08)	-1.03 (-2.23, 0.04)	-1.46 (-2.65, -0.11)	-1.99 (-3.67, -0.3)	-3.4 (-5.47, -0.87)
Black African	-0.5 (-1.54, 0.43)	-0.53 (-1.42, 0.41)	-0.47 (-1.49, 0.58)	-0.44 (-1.57, 0.72)	-0.63 (-1.66, 0.48)	-0.73 (-2.03, 0.76)	-0.72 (-2.15, 0.84)	-1.02 (-2.76, 0.84)	-1.73 (-3.87, 1.2)
Black Caribbean	-1.74 (-2.87, -0.55)	-1.22 (-2.1, -0.36)	-1.11 (-1.89, -0.35)	-1.13 (-1.98, -0.02)	-0.84 (-2.06, 0.18)	-0.74 (-1.76, 0.41)	-0.6 (-1.88, 0.87)	-0.73 (-2.37, 1.34)	-1.19 (-3.51, 1.99)
Other	-0.85 (-3.2, 0.6)	-0.33 (-1.52, 0.75)	-0.17 (-1.25, 1.11)	0.25 (-1.19, 1.65)	0.45 (-0.89, 2.04)	1.03 (-0.7, 2.45)	1.11 (-0.4, 2.67)	0.8 (-0.85, 3.25)	-0.1 (-2.5, 2.87)
Constant	4.43 (2.92, 5.72)	5.11 (3.9, 6.29)	6.3 (5.06, 7.44)	7.06 (5.87, 8.29)	7.73 (6.39, 9.08)	8.1 (6.7, 9.48)	9.06 (7.37, 10.81)	10.91 (8.31, 13.49)	13.51 (10.18, 16.73)
Observations	7,363	7,363	7,363	7,363	7,363	7,363	7,363	7,363	7,363
Imputations	28	28	28	28	28	28	28	28	28

Table 5: Quantile Regression Results. Fully adjusted model including current economic activity (Column D, Table 2 in main paper)

	Variable	Q10	Q20	Q30	Q40	Q50	Q60	Q70	Q80	Q90
	6+ Months Unemployment	0.16 (-0.68, 0.98)	0.41 (-0.3, 1.01)	0.25 (-0.41, 0.91)	0.21 (-0.53, 0.96)	0.26 (-0.6, 1.12)	0.59 (-0.35, 1.78)	1.61 (0.19, 2.85)	2.24 (0.78, 3.94)	2.58 (0.88, 4.27)
	Female	0.44 (0.08, 0.8)	0.54 (0.23, 0.84)	0.45 (0.12, 0.77)	0.3 (-0.04, 0.64)	0.2 (-0.17, 0.57)	0.2 (-0.18, 0.59)	0.12 (-0.35, 0.58)	-0.17 (-0.79, 0.42)	-0.76 (-1.62, 0.08)
	IMD	0 (-0.02, 0.02)	0 (-0.01, 0.01)	0 (-0.01, 0.01)	0 (-0.02, 0.01)	0 (-0.01, 0.01)	0 (-0.01, 0.02)	0 (-0.02, 0.02)	0 (-0.02, 0.02)	0.02 (-0.01, 0.05)
	Locus of Control	-0.21 (-0.4, -0.02)	-0.1 (-0.3, 0.08)	-0.06 (-0.26, 0.14)	-0.08 (-0.28, 0.13)	-0.05 (-0.28, 0.18)	-0.01 (-0.24, 0.22)	0.06 (-0.22, 0.36)	0.13 (-0.25, 0.5)	0.01 (-0.49, 0.5)
Current Economic Activity	Education	0.69 (-0.31, 1.5)	0.59 (-0.07, 1.25)	0.56 (-0.19, 1.36)	0.66 (-0.16, 1.61)	0.84 (-0.12, 1.79)	1.14 (0.16, 2.19)	1.59 (0.3, 3.04)	2.22 (0.35, 4.23)	2.27 (0.44, 4.39)
	Inactive	0.54 (-0.07, 1.28)	0.37 (-0.28, 1.01)	0.48 (-0.27, 1.25)	0.83 (-0.05, 1.69)	1.18 (0.27, 2.35)	1.86 (0.67, 3.08)	2.66 (1.22, 4.28)	3.76 (1.9, 5.95)	4.6 (2.56, 6.77)
	Unemployed	1.5 (0.42, 2.6)	2.15 (0.99, 3.02)	2.37 (1.54, 3.16)	2.52 (1.66, 3.52)	2.84 (1.69, 3.99)	3.29 (2.14, 4.46)	3.97 (2.46, 5.8)	4.73 (2.98, 6.32)	4.69 (2.89, 6.58)
	GHQ-12 @ Age 14/15	0.09 (-0.01, 0.18)	0.12 (0.03, 0.2)	0.1 (0.02, 0.18)	0.12 (0.02, 0.21)	0.17 (0.07, 0.27)	0.24 (0.13, 0.35)	0.29 (0.16, 0.43)	0.37 (0.2, 0.57)	0.51 (0.29, 0.74)
	GHQ-12 @ Age 16/17	0.12 (0.09, 0.17)	0.16 (0.12, 0.2)	0.19 (0.15, 0.22)	0.2 (0.17, 0.24)	0.22 (0.18, 0.27)	0.26 (0.21, 0.3)	0.29 (0.23, 0.35)	0.32 (0.24, 0.39)	0.37 (0.28, 0.45)
Self-Rated Health @ Age 14/15	Fairly Good	0.16 (-0.26, 0.55)	0.24 (-0.09, 0.57)	0.32 (-0.04, 0.67)	0.43 (0.06, 0.78)	0.45 (0.04, 0.87)	0.35 (-0.06, 0.78)	0.32 (-0.18, 0.82)	0.49 (-0.17, 1.18)	1.04 (0.1, 2.01)

	Variable	Q10	Q20	Q30	Q40	Q50	Q60	Q70	Q80	Q90
	Not Very Good	0.34 (-1.05, 1.63)	0.72 (-0.71, 1.85)	0.65 (-0.54, 2.06)	1 (-0.56, 2.19)	0.92 (-0.49, 2.08)	0.61 (-0.65, 2.28)	0.94 (-0.87, 3.76)	1.73 (-1.1, 5.34)	2.62 (-0.21, 5.53)
	Not Good at All	-0.16 (-2.2, 1.71)	-0.37 (-2.43, 1.44)	-0.69 (-2.28, 1.8)	-0.47 (-2.83, 2.13)	-0.49 (-3.15, 2.99)	-0.69 (-3.28, 3.39)	-1 (-3.89, 4.82)	-0.39 (-4.72, 4.85)	-0.25 (-5.14, 5.73)
Self-Rated Health @ Age 16/17	Fairly Good	0.23 (-0.19, 0.67)	0.2 (-0.15, 0.54)	0.12 (-0.25, 0.49)	0.18 (-0.2, 0.57)	0.18 (-0.23, 0.56)	0.05 (-0.38, 0.46)	-0.03 (-0.54, 0.5)	0.22 (-0.45, 0.9)	0.18 (-0.83, 1.2)
	Not Very Good	-0.11 (-0.97, 0.81)	-0.06 (-1.13, 0.89)	0.29 (-0.71, 1.31)	0.55 (-0.35, 1.38)	0.5 (-0.38, 1.37)	0.35 (-0.56, 1.52)	0.47 (-0.83, 2.03)	0.64 (-1.1, 2.19)	-0.36 (-1.98, 1.47)
	Not Good at All	-0.94 (-2.93, 1.76)	-0.16 (-2.62, 2)	0.34 (-2.53, 2.17)	0.47 (-1.63, 2.44)	0.89 (-1.52, 2.84)	0.66 (-1.28, 4.32)	1.15 (-1.46, 5.32)	1.26 (-2.04, 4.86)	-1.15 (-4.11, 2.18)
Disabled	Yes, school not affected	0.13 (-0.5, 0.72)	0.06 (-0.46, 0.55)	-0.03 (-0.53, 0.49)	-0.08 (-0.66, 0.58)	0.11 (-0.59, 0.78)	0.24 (-0.43, 1.1)	0.62 (-0.32, 1.54)	0.6 (-0.5, 1.92)	1.21 (-0.57, 3.03)
	Yes, school affected	0.19 (-0.93, 1.16)	0.34 (-0.55, 1.08)	0.32 (-0.41, 1.01)	0.15 (-0.57, 0.99)	0.29 (-0.66, 1.17)	0.41 (-0.56, 1.36)	0.5 (-0.57, 1.94)	0.8 (-0.8, 2.62)	1.37 (-1.08, 4.53)
	Risk Behaviours	-0.06 (-0.22, 0.06)	-0.1 (-0.21, 0.02)	-0.1 (-0.23, 0.06)	-0.07 (-0.23, 0.08)	-0.05 (-0.2, 0.11)	-0.03 (-0.21, 0.16)	0 (-0.21, 0.2)	-0.01 (-0.27, 0.31)	-0.06 (-0.4, 0.28)
	Attitude to School	-0.02 (-0.05, 0.01)	-0.01 (-0.04, 0.02)	-0.02 (-0.05, 0.01)	-0.02 (-0.05, 0.01)	-0.02 (-0.05, 0.01)	-0.03 (-0.06, 0)	-0.03 (-0.07, 0)	-0.05 (-0.11, 0.01)	-0.05 (-0.12, 0.03)
	# Waves Bullied, 1-3	0.09 (-0.08, 0.27)	0.11 (-0.03, 0.24)	0.16 (0.01, 0.32)	0.22 (0.06, 0.38)	0.24 (0.07, 0.42)	0.31 (0.13, 0.49)	0.37 (0.15, 0.59)	0.53 (0.2, 0.87)	0.66 (0.27, 1.08)

	Variable	Q10	Q20	Q30	Q40	Q50	Q60	Q70	Q80	Q90
Qualifications	NVQ 4	0.46 (-0.07, 1)	0.33 (-0.08, 0.75)	0.36 (-0.11, 0.83)	0.3 (-0.19, 0.78)	0.29 (-0.2, 0.79)	0.37 (-0.15, 0.91)	0.46 (-0.22, 1.11)	0.42 (-0.54, 1.33)	0.25 (-1.14, 1.54)
	NVQ 3	0.51 (-0.09, 1.1)	0.39 (-0.04, 0.84)	0.34 (-0.14, 0.82)	0.28 (-0.27, 0.82)	0.38 (-0.19, 0.92)	0.49 (-0.1, 1.05)	0.52 (-0.16, 1.18)	0.35 (-0.65, 1.38)	0.27 (-1.15, 1.6)
	NVQ 2	0.22 (-0.37, 0.84)	0.25 (-0.2, 0.71)	0.23 (-0.28, 0.73)	0.11 (-0.44, 0.66)	0.01 (-0.53, 0.56)	-0.07 (-0.68, 0.51)	-0.13 (-0.89, 0.6)	-0.63 (-1.68, 0.37)	-1.1 (-2.56, 0.29)
	NVQ 1	-0.68 (-1.6, 0.18)	-0.61 (-1.29, 0.15)	-0.37 (-1.09, 0.35)	-0.45 (-1.21, 0.36)	-0.28 (-1.13, 0.54)	0.03 (-0.86, 0.89)	0.03 (-1.02, 1.12)	0.21 (-1.4, 1.82)	0.27 (-1.78, 2.36)
	No/Other Qual	-0.54 (-1.55, 0.41)	-0.3 (-1.16, 0.49)	-0.31 (-1.12, 0.56)	-0.26 (-1.18, 0.64)	-0.17 (-1.1, 0.74)	0.02 (-0.97, 0.97)	0.23 (-0.99, 1.54)	-0.08 (-1.74, 1.7)	-1.15 (-3.23, 1.12)
Parental NS-SEC	Intermediate	0.14 (-0.38, 0.66)	0.1 (-0.31, 0.49)	-0.01 (-0.4, 0.39)	-0.1 (-0.52, 0.32)	-0.16 (-0.67, 0.35)	-0.07 (-0.61, 0.47)	0.08 (-0.52, 0.69)	0.36 (-0.5, 1.24)	0.58 (-0.69, 1.9)
	Routine	0.26 (-0.22, 0.73)	0.25 (-0.15, 0.65)	0.29 (-0.13, 0.69)	0.32 (-0.13, 0.76)	0.26 (-0.21, 0.73)	0.17 (-0.34, 0.7)	0.35 (-0.24, 0.95)	0.66 (-0.16, 1.41)	0.45 (-0.8, 1.73)
	LTU	-0.17 (-1.47, 1)	-0.19 (-1.27, 0.77)	-0.13 (-1.13, 0.96)	0.1 (-1.06, 1.35)	0.25 (-1.04, 1.41)	0.15 (-1.01, 1.55)	0.77 (-0.85, 2.62)	1.24 (-0.75, 3.55)	1.74 (-1.01, 4.3)
Parental Education	Other HE	-0.23 (-0.8, 0.3)	-0.08 (-0.54, 0.36)	-0.15 (-0.71, 0.42)	0.04 (-0.52, 0.57)	0.09 (-0.47, 0.63)	0.12 (-0.46, 0.71)	0.17 (-0.55, 0.86)	0.17 (-0.75, 1.16)	0.66 (-0.58, 2.01)
	A-Level	-0.26 (-0.88, 0.32)	-0.16 (-0.64, 0.3)	-0.34 (-0.86, 0.19)	-0.27 (-0.82, 0.28)	-0.15 (-0.75, 0.47)	-0.1 (-0.72, 0.5)	-0.22 (-0.94, 0.49)	-0.39 (-1.3, 0.59)	-0.09 (-1.49, 1.47)

	Variable	Q10	Q20	Q30	Q40	Q50	Q60	Q70	Q80	Q90
	GCSE A-C	-0.05 (-0.63, 0.53)	0.05 (-0.43, 0.52)	-0.07 (-0.57, 0.45)	-0.15 (-0.65, 0.34)	-0.14 (-0.71, 0.44)	-0.03 (-0.63, 0.58)	0 (-0.7, 0.72)	-0.3 (-1.2, 0.66)	-0.16 (-1.63, 1.21)
	Other/None	-0.52 (-1.21, 0.2)	-0.09 (-0.71, 0.51)	-0.06 (-0.68, 0.56)	0.02 (-0.63, 0.69)	0.32 (-0.43, 1.08)	0.45 (-0.34, 1.23)	0.3 (-0.63, 1.26)	0.5 (-0.78, 1.85)	1.5 (-0.35, 3.3)
Ethnicity	Mixed	-0.79 (-1.92, 0.19)	-0.63 (-1.53, 0.11)	-0.67 (-1.55, 0.16)	-0.75 (-1.66, -0.03)	-1 (-1.75, -0.07)	-0.92 (-2, 0.45)	-0.35 (-1.8, 1.65)	0.41 (-1.35, 2.84)	1.84 (-1.35, 5.15)
	Indian	-1.2 (-1.93, -0.41)	-0.86 (-1.55, -0.23)	-0.75 (-1.45, -0.02)	-0.62 (-1.39, 0.17)	-0.57 (-1.35, 0.19)	-0.41 (-1.23, 0.43)	-0.24 (-1.25, 0.95)	0.09 (-1.26, 1.34)	-0.74 (-2.26, 1.4)
	Pakistani	-0.62 (-1.54, 0.23)	-0.62 (-1.38, 0.05)	-0.75 (-1.44, -0.06)	-0.98 (-1.73, -0.23)	-1.28 (-2.06, -0.46)	-1.23 (-2.07, -0.34)	-1.03 (-2.16, 0.18)	-1.06 (-2.4, 0.29)	-2.02 (-3.83, 0.52)
	Bangladeshi	-1.69 (-2.95, -0.35)	-1.25 (-2.71, -0.08)	-0.79 (-2.01, 0.15)	-0.82 (-1.83, 0.16)	-1.06 (-2.09, 0.18)	-0.92 (-2.13, 0.22)	-1.06 (-2.34, 0.29)	-1.44 (-3.17, 0.12)	-2.75 (-4.75, -0.37)
	Black African	-0.49 (-1.48, 0.44)	-0.51 (-1.38, 0.36)	-0.54 (-1.48, 0.47)	-0.65 (-1.66, 0.32)	-0.97 (-2.02, 0.35)	-0.76 (-2.19, 0.83)	-0.55 (-2.04, 0.96)	-0.62 (-2.27, 1.24)	-1.21 (-3.32, 1.66)
	Black Caribbean	-1.69 (-2.8, -0.58)	-1.29 (-2.14, -0.46)	-1.23 (-2.01, -0.43)	-1.29 (-2.14, -0.2)	-1.07 (-2.25, 0.04)	-0.85 (-1.95, 0.23)	-0.77 (-1.94, 0.7)	-0.81 (-2.36, 1.36)	-1.37 (-3.59, 0.91)
	Other	-0.96 (-3.1, 0.39)	-0.46 (-1.85, 0.68)	-0.22 (-1.32, 0.88)	-0.12 (-1.34, 1.39)	0.31 (-1.3, 1.93)	0.74 (-0.94, 2.19)	0.89 (-0.74, 2.52)	0.62 (-1.13, 3.03)	0.4 (-2.4, 3.46)
	Constant	4.41 (2.87, 5.69)	4.96 (3.81, 6.19)	6.38 (5.11, 7.53)	7.08 (5.89, 8.3)	7.7 (6.36, 9.06)	8.41 (6.95, 9.84)	9.28 (7.59, 11.09)	11.09 (8.62, 13.54)	13.07 (9.98, 16.31)

Variable	Q10	Q20	Q30	Q40	Q50	Q60	Q70	Q80	Q90
Observations	7,363	7,363	7,363	7,363	7,363	7,363	7,363	7,363	7,363
Imputations	28	28	28	28	28	28	28	28	28

For peer review only

Table 6: Quantile Regression Results. Fully adjusted model, males only (Column E, Table 2 in main paper)

	Variable	Q10	Q20	Q30	Q40	Q50	Q60	Q70	Q80	Q90
	6+ Months Unemployment	0.85 (-0.15, 1.81)	0.77 (-0.12, 1.6)	0.56 (-0.34, 1.52)	0.56 (-0.37, 1.63)	0.77 (-0.31, 1.93)	1.06 (-0.18, 2.62)	1.88 (0.01, 4.02)	3.28 (0.69, 6.3)	4.16 (1.2, 7.14)
	IMD	-0.01 (-0.03, 0.02)	-0.01 (-0.02, 0.01)	-0.01 (-0.03, 0.01)	-0.01 (-0.03, 0.02)	0 (-0.02, 0.02)	0 (-0.02, 0.02)	0 (-0.03, 0.03)	0 (-0.03, 0.05)	0.01 (-0.03, 0.06)
	Locus of Control	-0.08 (-0.38, 0.22)	-0.07 (-0.36, 0.21)	-0.15 (-0.49, 0.17)	-0.26 (-0.58, 0.09)	-0.22 (-0.54, 0.11)	-0.14 (-0.46, 0.21)	0.01 (-0.4, 0.45)	0.17 (-0.45, 0.77)	0.31 (-0.51, 1.06)
	GHQ-12 @ Age 14/15	0.07 (-0.12, 0.23)	0.08 (-0.07, 0.21)	0.05 (-0.1, 0.22)	0.08 (-0.09, 0.27)	0.19 (0, 0.39)	0.3 (0.1, 0.49)	0.39 (0.16, 0.68)	0.57 (0.22, 0.94)	0.65 (0.2, 1.1)
	GHQ-12 @ Age 16/17	0.18 (0.11, 0.24)	0.21 (0.15, 0.26)	0.24 (0.18, 0.3)	0.25 (0.19, 0.32)	0.26 (0.19, 0.33)	0.27 (0.2, 0.35)	0.29 (0.2, 0.4)	0.32 (0.19, 0.46)	0.41 (0.23, 0.58)
Self-Rated Health @ Age 14/15	Fairly Good	0.14 (-0.44, 0.74)	0.25 (-0.27, 0.75)	0.27 (-0.29, 0.81)	0.32 (-0.27, 0.87)	0.33 (-0.28, 0.93)	0.24 (-0.42, 0.93)	0.18 (-0.66, 1.05)	0.61 (-0.54, 1.8)	1.39 (-0.24, 2.99)
	Not Very Good	-0.12 (-2.1, 2.38)	0.18 (-2.17, 2.44)	0.18 (-2.1, 2.73)	0.3 (-2.12, 2.94)	0.66 (-2.48, 3.09)	0.48 (-2.26, 3.53)	0.3 (-2.78, 6.59)	0.49 (-3.43, 8.71)	2.77 (-4.13, 8.31)
	Not Good at All	1.27 (-2.77, 4.31)	0.82 (-2.04, 4.43)	0.39 (-2.83, 5.01)	0.07 (-3.28, 4.92)	-0.09 (-3.19, 5.94)	-0.41 (-3.47, 9.15)	-0.93 (-4.89, 9.35)	-0.74 (-6.2, 8.7)	-0.68 (-6.84, 9.78)
Self-Rated Health @ Age 16/17	Fairly Good	0.26 (-0.39, 0.88)	0.14 (-0.41, 0.7)	0.1 (-0.46, 0.66)	0.15 (-0.45, 0.74)	0.1 (-0.52, 0.73)	0.13 (-0.56, 0.8)	0.25 (-0.63, 1.14)	0.1 (-1.04, 1.24)	0.14 (-1.52, 1.89)
	Not Very Good	0.42 (-1.11, 1.89)	0.09 (-1.56, 2.02)	0.35 (-1.59, 2.3)	0.7 (-1.3, 2.15)	0.6 (-0.91, 2.32)	0.67 (-1.06, 3.26)	1.02 (-1.48, 4.04)	1.13 (-2.04, 4.56)	0.27 (-3.05, 3.93)

	Variable	Q10	Q20	Q30	Q40	Q50	Q60	Q70	Q80	Q90
	Not Good at All	0.85 (-5.04, 3.54)	0.78 (-3.73, 3.44)	0.27 (-3.46, 3.02)	0.41 (-3.13, 2.7)	0.58 (-3.15, 6.1)	0.32 (-3.15, 8.41)	0.71 (-2.76, 9.03)	1.95 (-3.43, 8.17)	-1.28 (-5.49, 5.53)
Disabled	Yes, school not affected	0.04 (-0.84, 0.91)	0.05 (-0.73, 0.74)	-0.15 (-0.89, 0.58)	-0.24 (-1.04, 0.68)	-0.12 (-1.01, 0.8)	0 (-0.95, 1.11)	0.17 (-0.99, 1.35)	0.08 (-1.36, 2.19)	1.11 (-1.39, 3.94)
	Yes, school affected	0.54 (-0.97, 1.84)	0.75 (-0.47, 1.87)	0.7 (-0.47, 1.9)	0.69 (-0.53, 1.88)	0.77 (-0.45, 1.99)	0.93 (-0.43, 2.56)	1.36 (-0.53, 3.75)	1.65 (-0.67, 5.33)	2.97 (-0.88, 6.52)
	Risk Behaviours	0 (-0.2, 0.18)	-0.04 (-0.22, 0.15)	-0.03 (-0.23, 0.18)	-0.03 (-0.23, 0.17)	-0.02 (-0.23, 0.22)	0.01 (-0.24, 0.29)	0.02 (-0.31, 0.39)	0.06 (-0.38, 0.65)	0.16 (-0.42, 0.67)
	Attitude to School	0 (-0.05, 0.06)	-0.01 (-0.05, 0.04)	-0.02 (-0.06, 0.03)	-0.02 (-0.07, 0.03)	-0.02 (-0.07, 0.03)	-0.03 (-0.09, 0.02)	-0.05 (-0.12, 0.01)	-0.07 (-0.15, 0.01)	-0.08 (-0.18, 0.04)
	# Waves Bullied, 1-3	0.22 (-0.05, 0.49)	0.16 (-0.05, 0.37)	0.22 (-0.02, 0.47)	0.31 (0.06, 0.55)	0.33 (0.07, 0.56)	0.36 (0.08, 0.63)	0.39 (0.05, 0.75)	0.51 (-0.03, 1.08)	0.72 (0.02, 1.46)
Qualifications	NVQ 4	0.49 (-0.31, 1.47)	0.44 (-0.27, 1.15)	0.44 (-0.33, 1.19)	0.18 (-0.64, 0.98)	-0.08 (-0.9, 0.79)	-0.07 (-0.91, 0.8)	0 (-1.07, 1.02)	0.16 (-1.4, 1.6)	-0.13 (-2.67, 2.28)
	NVQ 3	0.9 (-0.01, 1.86)	0.77 (0.09, 1.46)	0.75 (-0.03, 1.56)	0.62 (-0.29, 1.51)	0.36 (-0.51, 1.25)	0.24 (-0.61, 1.08)	0.08 (-0.96, 1.08)	-0.03 (-1.56, 1.54)	-0.58 (-3.27, 1.92)
	NVQ 2	0.66 (-0.24, 1.66)	0.71 (0.01, 1.42)	0.67 (-0.15, 1.47)	0.38 (-0.53, 1.24)	-0.06 (-0.92, 0.85)	-0.26 (-1.19, 0.66)	-0.33 (-1.48, 0.76)	-0.72 (-2.34, 0.78)	-1.73 (-4.32, 0.72)
	NVQ 1	-0.68 (-1.97, 0.66)	-0.33 (-1.38, 0.72)	-0.2 (-1.3, 0.88)	-0.38 (-1.51, 0.8)	-0.33 (-1.49, 0.85)	-0.23 (-1.44, 0.99)	-0.05 (-1.65, 1.75)	0.41 (-1.83, 2.91)	0.53 (-2.89, 4.14)

	Variable	Q10	Q20	Q30	Q40	Q50	Q60	Q70	Q80	Q90
	No/Other Qual	-0.54 (-1.85, 0.85)	-0.41 (-1.62, 0.94)	-0.09 (-1.41, 1.23)	-0.24 (-1.58, 1.11)	-0.41 (-1.8, 1.04)	-0.27 (-1.86, 1.43)	0.23 (-1.74, 2.49)	0.27 (-2.24, 3.25)	-0.89 (-4.26, 2.9)
Parental NS-SEC	Intermediate	-0.21 (-0.95, 0.53)	-0.37 (-0.96, 0.24)	-0.42 (-1.07, 0.2)	-0.51 (-1.21, 0.18)	-0.49 (-1.26, 0.31)	-0.24 (-1.04, 0.63)	0.15 (-0.88, 1.2)	0.44 (-0.93, 1.89)	0.93 (-1.13, 3.22)
	Routine	-0.27 (-0.96, 0.47)	-0.22 (-0.81, 0.38)	-0.12 (-0.79, 0.53)	-0.12 (-0.82, 0.54)	-0.18 (-0.87, 0.49)	-0.18 (-0.9, 0.6)	-0.05 (-0.98, 0.87)	-0.04 (-1.28, 1.25)	0.1 (-1.76, 2.06)
	LTU	-0.25 (-1.69, 1.13)	-0.52 (-1.87, 1.25)	-0.26 (-1.92, 1.47)	-0.33 (-1.99, 1.56)	-0.27 (-2.07, 1.6)	-0.16 (-2, 2.02)	0.06 (-2.36, 3)	0.5 (-2.66, 4.47)	1.49 (-2.72, 5.52)
Parental Education	Other HE	-0.41 (-1.33, 0.42)	-0.17 (-0.89, 0.53)	-0.2 (-0.99, 0.65)	-0.05 (-0.86, 0.75)	0.15 (-0.69, 0.95)	0.16 (-0.73, 1.05)	0.1 (-1.04, 1.27)	0.13 (-1.39, 1.79)	0.9 (-1.36, 3.65)
	A-Level	0.18 (-0.66, 1)	0.27 (-0.4, 0.94)	0.12 (-0.67, 0.94)	0.24 (-0.59, 1.07)	0.45 (-0.41, 1.31)	0.55 (-0.39, 1.45)	0.42 (-0.67, 1.62)	0.52 (-1.11, 2.25)	1.15 (-1.09, 3.62)
	GCSE A-C	0.2 (-0.66, 1.06)	0.43 (-0.29, 1.14)	0.34 (-0.42, 1.09)	0.28 (-0.43, 1.08)	0.38 (-0.44, 1.21)	0.34 (-0.53, 1.19)	0.15 (-0.94, 1.22)	-0.1 (-1.62, 1.38)	-0.33 (-2.63, 1.95)
	Other/None	0.03 (-1.01, 1.11)	0.24 (-0.66, 1.12)	0.38 (-0.64, 1.43)	0.78 (-0.29, 1.82)	1.18 (0.16, 2.21)	1.29 (0.16, 2.5)	1.62 (0.13, 3.1)	2.01 (0.06, 4.01)	3.16 (0.35, 5.85)
Ethnicity	Mixed	-0.6 (-1.89, 0.72)	-0.52 (-1.87, 0.77)	-0.43 (-1.89, 0.71)	-0.56 (-1.9, 0.54)	-0.83 (-2.02, 0.7)	-0.73 (-2.23, 1.23)	-0.45 (-2.4, 1.69)	-0.54 (-2.72, 2.24)	-0.3 (-3.51, 5.13)
	Indian	-1.26 (-2.28, -0.24)	-0.93 (-1.95, 0.12)	-0.72 (-1.91, 0.52)	-0.6 (-1.83, 0.54)	-0.73 (-1.88, 0.54)	-0.59 (-1.91, 0.93)	-0.47 (-2.04, 1.64)	0.05 (-2.41, 4.51)	1.67 (-2.48, 5.3)

Variable	Q10	Q20	Q30	Q40	Q50	Q60	Q70	Q80	Q90
Pakistani	-0.59 (-2.3, 0.57)	-0.51 (-1.61, 0.46)	-0.88 (-2, 0.08)	-1.4 (-2.48, -0.31)	-1.81 (-2.97, -0.5)	-1.74 (-3.07, -0.45)	-1.72 (-3.27, -0.18)	-2.27 (-4.12, -0.35)	-3.22 (-5.94, 0.36)
Bangladeshi	-1.26 (-3.26, 0.65)	-0.68 (-3.09, 1.16)	-0.41 (-2.6, 1.17)	-0.61 (-2.37, 1.03)	-0.9 (-2.63, 0.93)	-0.94 (-2.72, 0.92)	-1.2 (-3.24, 0.92)	-1.94 (-4.3, 0.4)	-3.35 (-6.09, -0.08)
Black African	-0.86 (-2.99, 0.57)	-0.95 (-2.3, 0.33)	-1.14 (-2.5, 0.92)	-0.88 (-2.79, 0.77)	-1.1 (-2.86, 0.32)	-1.66 (-3.13, 0.11)	-2.1 (-3.96, -0.02)	-2.9 (-4.91, -0.39)	-4.24 (-6.65, -0.39)
Black Caribbean	-1.3 (-3.49, 0.92)	-0.05 (-1.76, 1.07)	-0.29 (-1.47, 1.02)	-0.39 (-1.77, 1.13)	-0.25 (-1.87, 1.37)	-0.15 (-1.8, 1.9)	0.12 (-1.84, 2.58)	0.04 (-2.48, 4.29)	-0.82 (-4.03, 4.27)
Other	0.32 (-3.55, 1.9)	0.55 (-0.96, 1.8)	0.39 (-1.01, 2.49)	0.81 (-1.19, 3.17)	1 (-1.1, 3.33)	1.35 (-1.01, 3.39)	1.25 (-1.16, 3.91)	1.02 (-1.75, 5.21)	-0.08 (-3.32, 4.47)
Constant	3.06 (0.3, 5.4)	4.44 (2.46, 6.31)	5.46 (3.51, 7.38)	6.49 (4.58, 8.34)	7.41 (5.46, 9.44)	8.41 (6.16, 10.79)	9.92 (7.11, 12.85)	11.64 (7.87, 15.59)	13.62 (8.35, 18.59)
Observations	3,196	3,196	3,196	3,196	3,196	3,196	3,196	3,196	3,196
Imputations	28	28	28	28	28	28	28	28	28

Table 7: Quantile Regression Results. Fully adjusted model, females only (Column F, Table 2 in main paper)

	Variable	Q10	Q20	Q30	Q40	Q50	Q60	Q70	Q80	Q90
	6+ Months Unemployment	-0.47 (-1.61, 0.95)	0.29 (-1.06, 1.33)	0.43 (-0.57, 1.41)	0.46 (-0.55, 1.49)	0.61 (-0.59, 1.97)	1.15 (-0.3, 3.05)	2.19 (0.24, 3.88)	2.33 (0.42, 4.36)	2.35 (0.32, 4.35)
	IMD	0.01 (-0.01, 0.02)	0 (-0.01, 0.02)	0 (-0.01, 0.02)	0 (-0.01, 0.02)	0.01 (-0.01, 0.02)	0.01 (-0.01, 0.03)	0.01 (-0.02, 0.04)	0.02 (-0.02, 0.05)	0.04 (0, 0.09)
	Locus of Control	-0.23 (-0.49, 0.04)	-0.15 (-0.41, 0.11)	-0.1 (-0.34, 0.15)	-0.03 (-0.3, 0.21)	-0.03 (-0.33, 0.25)	-0.05 (-0.4, 0.29)	0.05 (-0.36, 0.43)	0.07 (-0.48, 0.58)	-0.21 (-0.86, 0.45)
	GHQ-12 @ Age 14/15	0.09 (-0.02, 0.21)	0.13 (0.02, 0.23)	0.12 (0.03, 0.22)	0.14 (0.04, 0.25)	0.18 (0.07, 0.3)	0.23 (0.1, 0.37)	0.26 (0.1, 0.42)	0.3 (0.09, 0.52)	0.45 (0.18, 0.7)
	GHQ-12 @ Age 16/17	0.1 (0.05, 0.15)	0.14 (0.08, 0.19)	0.16 (0.12, 0.21)	0.17 (0.13, 0.22)	0.2 (0.14, 0.26)	0.25 (0.18, 0.32)	0.3 (0.23, 0.37)	0.35 (0.26, 0.44)	0.36 (0.25, 0.46)
Self-Rated Health @ Age 14/15	Fairly Good	0.24 (-0.27, 0.77)	0.24 (-0.21, 0.72)	0.41 (-0.04, 0.85)	0.47 (0, 0.92)	0.4 (-0.11, 0.94)	0.45 (-0.12, 1.04)	0.43 (-0.21, 1.05)	0.46 (-0.41, 1.37)	0.9 (-0.31, 2.08)
	Not Very Good	1.01 (-0.55, 2.49)	0.9 (-0.46, 2.41)	1.11 (-0.42, 2.71)	1.42 (-0.32, 2.65)	1.13 (-0.27, 2.71)	1.09 (-0.54, 3.35)	1.62 (-0.57, 5.21)	2.48 (-0.71, 5.51)	2.03 (-0.9, 5.04)
	Not Good at All	0.19 (-1.89, 1.9)	-0.63 (-2.52, 1.47)	-0.96 (-2.69, 3.06)	-1.18 (-3.46, 4.64)	-0.71 (-4.45, 5.25)	0.18 (-5.06, 5.85)	1 (-5.26, 7.05)	0.83 (-4.9, 8.16)	1.86 (-5.49, 8.44)
Self-Rated Health @ Age 16/17	Fairly Good	0.17 (-0.36, 0.68)	0.21 (-0.25, 0.68)	0.14 (-0.31, 0.61)	0.24 (-0.25, 0.7)	0.22 (-0.3, 0.71)	0.05 (-0.53, 0.63)	0.09 (-0.56, 0.76)	0.22 (-0.76, 1.15)	0.19 (-1, 1.35)
	Not Very Good	-0.56 (-1.93, 0.38)	-0.25 (-1.41, 0.95)	0.27 (-0.82, 1.32)	0.51 (-0.51, 1.5)	0.56 (-0.49, 1.64)	0.47 (-0.66, 1.9)	0.45 (-0.85, 2.08)	0.24 (-1.51, 2.21)	0.31 (-2.25, 2.92)

	Variable	Q10	Q20	Q30	Q40	Q50	Q60	Q70	Q80	Q90
	Not Good at All	-1.08 (-3.07, 1.85)	-0.4 (-3.32, 2.14)	-0.22 (-2.75, 2.46)	0.28 (-2.44, 3.31)	1.02 (-2.2, 5.21)	1.29 (-2.25, 6.24)	1.72 (-2.32, 5.89)	1.26 (-2.77, 5.86)	0.88 (-3.81, 6.04)
Disabled	Yes, school not affected	0.15 (-0.87, 0.98)	0.21 (-0.54, 0.98)	0.14 (-0.59, 0.89)	0.12 (-0.7, 1.12)	0.5 (-0.65, 1.86)	1.02 (-0.31, 2.35)	1.32 (-0.11, 2.87)	1.55 (-0.13, 3.99)	1.82 (-0.61, 4.19)
	Yes, school affected	0.17 (-1.23, 1.36)	-0.08 (-1.24, 0.94)	-0.1 (-1.16, 1)	-0.05 (-1.25, 1.21)	0.28 (-1.05, 1.5)	0.42 (-0.87, 1.76)	0.37 (-1.06, 2.4)	0.83 (-1.44, 3.51)	1.79 (-1.11, 4.79)
	Risk Behaviours	-0.03 (-0.23, 0.1)	-0.13 (-0.25, 0.01)	-0.19 (-0.34, 0.05)	-0.12 (-0.35, 0.12)	-0.04 (-0.27, 0.18)	-0.01 (-0.24, 0.25)	0.01 (-0.25, 0.3)	0.03 (-0.34, 0.44)	-0.08 (-0.52, 0.33)
	Attitude to School	-0.04 (-0.07, 0)	-0.03 (-0.06, 0.01)	-0.03 (-0.07, 0)	-0.02 (-0.06, 0.01)	-0.02 (-0.06, 0.02)	-0.01 (-0.05, 0.03)	-0.02 (-0.07, 0.04)	-0.03 (-0.11, 0.05)	-0.03 (-0.12, 0.06)
	# Waves Bullied, 1-3	-0.03 (-0.24, 0.19)	0.09 (-0.1, 0.28)	0.13 (-0.06, 0.33)	0.17 (-0.02, 0.37)	0.24 (0.03, 0.45)	0.35 (0.1, 0.6)	0.48 (0.17, 0.8)	0.64 (0.24, 1.03)	0.69 (0.19, 1.23)
Qualifications	NVQ 4	0.47 (-0.29, 1.19)	0.27 (-0.3, 0.9)	0.32 (-0.25, 0.93)	0.45 (-0.14, 1.03)	0.52 (-0.11, 1.14)	0.67 (-0.05, 1.37)	0.65 (-0.28, 1.51)	0.44 (-0.76, 1.63)	0.32 (-1.21, 1.82)
	NVQ 3	0.23 (-0.58, 0.98)	0.02 (-0.59, 0.66)	-0.09 (-0.7, 0.53)	0.04 (-0.6, 0.69)	0.27 (-0.42, 0.95)	0.54 (-0.28, 1.37)	0.65 (-0.31, 1.54)	0.47 (-0.78, 1.78)	0.77 (-0.84, 2.54)
	NVQ 2	-0.21 (-1.07, 0.63)	-0.31 (-1.02, 0.38)	-0.29 (-0.91, 0.34)	-0.17 (-0.83, 0.52)	-0.04 (-0.77, 0.67)	0.04 (-0.77, 0.88)	0.07 (-0.99, 1.08)	-0.36 (-1.76, 1.06)	-0.85 (-2.5, 0.91)
	NVQ 1	-0.37 (-1.67, 0.72)	-0.51 (-1.48, 0.56)	-0.33 (-1.34, 0.65)	-0.21 (-1.26, 0.85)	-0.07 (-1.24, 1.1)	0.31 (-1.01, 1.54)	0.48 (-1.04, 2.13)	0.63 (-1.42, 2.56)	0.04 (-2.21, 2.54)

	Variable	Q10	Q20	Q30	Q40	Q50	Q60	Q70	Q80	Q90
	No/Other Qual	0.03 (-1.46, 1.3)	-0.11 (-1.08, 0.95)	-0.16 (-1.23, 0.88)	-0.02 (-1.1, 1.13)	0.22 (-1.02, 1.46)	0.58 (-0.77, 1.99)	0.93 (-0.78, 3.01)	1.03 (-1.37, 3.46)	0.23 (-2.33, 2.99)
Parental NS-SEC	Intermediate	0.54 (-0.12, 1.23)	0.45 (-0.12, 1.02)	0.45 (-0.07, 0.98)	0.36 (-0.21, 0.97)	0.32 (-0.36, 0.96)	0.14 (-0.62, 0.87)	-0.1 (-0.9, 0.73)	-0.06 (-1.14, 1.07)	0.07 (-1.53, 1.57)
	Routine	0.63 (0.02, 1.3)	0.72 (0.18, 1.25)	0.8 (0.22, 1.34)	0.8 (0.22, 1.36)	0.8 (0.15, 1.45)	0.83 (0.02, 1.63)	0.78 (-0.02, 1.68)	1.19 (0.07, 2.33)	1.39 (-0.14, 2.87)
	LTU	-0.46 (-2.95, 1.51)	0 (-1.62, 1.31)	0.02 (-1.31, 1.33)	0.12 (-1.26, 1.91)	0.58 (-1.22, 2.34)	1.01 (-0.93, 3.15)	1.79 (-0.39, 4.02)	2.31 (-0.32, 5.04)	2.19 (-0.7, 5.26)
Parental Education	Other HE	-0.1 (-0.87, 0.64)	-0.1 (-0.78, 0.55)	-0.11 (-0.78, 0.59)	0.03 (-0.71, 0.75)	0.03 (-0.73, 0.79)	0.01 (-0.87, 0.87)	0.11 (-0.91, 1.11)	0.09 (-1.17, 1.33)	0.07 (-1.62, 1.72)
	A-Level	-0.44 (-1.31, 0.35)	-0.61 (-1.31, 0.07)	-0.73 (-1.39, -0.03)	-0.72 (-1.45, -0.02)	-0.88 (-1.65, -0.05)	-0.91 (-1.83, 0.01)	-1 (-2.02, -0.01)	-1.39 (-2.66, -0.16)	-1.8 (-3.59, 0.06)
	GCSE A-C	-0.42 (-1.27, 0.37)	-0.49 (-1.15, 0.18)	-0.48 (-1.12, 0.18)	-0.54 (-1.2, 0.12)	-0.71 (-1.43, 0.08)	-0.59 (-1.5, 0.35)	-0.4 (-1.43, 0.58)	-0.46 (-1.79, 0.89)	-0.46 (-2.25, 1.33)
	Other/None	-0.31 (-1.32, 0.63)	-0.22 (-1.05, 0.61)	-0.32 (-1.09, 0.47)	-0.33 (-1.19, 0.52)	-0.36 (-1.29, 0.61)	-0.41 (-1.53, 0.71)	-0.62 (-1.89, 0.67)	-0.88 (-2.58, 0.79)	-0.76 (-2.84, 1.52)
Ethnicity	Mixed	-0.73 (-2.17, 0.54)	-0.59 (-1.69, 0.38)	-0.72 (-1.76, 0.25)	-0.95 (-1.98, 0.23)	-1 (-2.23, 0.5)	-0.75 (-2.26, 1.2)	0.02 (-2.13, 3.69)	1.67 (-1.47, 6.06)	3.36 (-0.92, 8.66)
	Indian	-1.27 (-2.58, -0.06)	-0.89 (-1.79, -0.17)	-0.98 (-1.7, -0.07)	-0.79 (-1.73, 0.15)	-0.61 (-1.6, 0.39)	-0.51 (-1.49, 0.54)	-0.31 (-1.51, 1.36)	0.12 (-1.61, 1.87)	-0.48 (-2.52, 1.65)

Variable	Q10	Q20	Q30	Q40	Q50	Q60	Q70	Q80	Q90
Pakistani	-0.76 (-2.03, 0.42)	-0.58 (-1.83, 0.42)	-0.42 (-1.41, 0.45)	-0.46 (-1.49, 0.54)	-0.53 (-1.63, 0.74)	-0.34 (-1.65, 1.06)	0.06 (-1.47, 1.71)	0 (-1.9, 2.11)	-0.37 (-3.11, 2.37)
Bangladeshi	-2.53 (-4.17, -0.35)	-1.73 (-3.89, -0.19)	-1.18 (-2.64, -0.18)	-1.22 (-2.37, 0.01)	-1.3 (-2.63, 0.2)	-1.24 (-2.8, 0.14)	-1.52 (-3, 0.22)	-1.9 (-3.96, 0.32)	-2.65 (-5.54, 1.08)
Black African	-0.25 (-1.31, 0.96)	-0.05 (-1.2, 0.93)	-0.29 (-1.27, 0.88)	-0.34 (-1.62, 1.55)	0.02 (-1.77, 2.1)	0.37 (-1.53, 2.39)	0.34 (-1.63, 2.57)	0.46 (-1.97, 3.36)	0.45 (-2.77, 4.73)
Black Caribbean	-2.29 (-3.51, -0.93)	-1.97 (-3, -1.03)	-1.94 (-2.91, -0.91)	-1.83 (-2.95, -0.47)	-1.57 (-3.04, -0.26)	-1.34 (-2.74, 0.11)	-1.22 (-2.72, 0.34)	-1.47 (-3.27, 0.45)	-2.29 (-4.59, 0.52)
Other	-1.31 (-3.66, 0.12)	-1.41 (-2.81, 0.28)	-0.91 (-2.69, 0.75)	-0.53 (-2.33, 1.33)	-0.05 (-2.07, 1.56)	0.16 (-1.71, 2.43)	0.85 (-1.3, 2.91)	0.69 (-1.55, 3.32)	-0.31 (-2.98, 2.33)
Constant	5.89 (4.36, 7.41)	6.61 (5.12, 8.13)	7.6 (6.12, 9.01)	7.9 (6.33, 9.58)	8.2 (6.32, 10.05)	8.09 (6.13, 9.99)	8.74 (6.45, 11.12)	10.18 (6.64, 13.67)	12.63 (8.67, 16.52)
Observations	4,167	4,167	4,167	4,167	4,167	4,167	4,167	4,167	4,167
Imputations	28	28	28	28	28	28	28	28	28

References

Buddelmeyer, H., & Powdthavee, N. (2016). Can having internal locus of control insure against negative shocks? Psychological evidence from panel data. *Journal of Economic Behavior & Organization*, *122*, 88–109. https://doi.org/10.1016/j.jebo.2015.11.014

Caliendo, M., Cobb-Clark, D. A., & Uhlendorff, A. (2015). Locus of control and job search strategies. *Review of Economics and Statistics*, *97*(1), 88–103. https://doi.org/10.1162/REST_a_00459

Chowdry, H., Crawford, C., & Goodman, A. (2011). The role of attitudes and behaviours in explaining socio-economic differences in attainment at age 16. *Longitudinal and Life Course Studies*, *2*(1). https://doi.org/10.14301/llcs.v2i1.141

Cobb-Clark, D. A. (2015). Locus of control and the labor market. *IZA Journal of Labor Economics*, *4*(1), 3. https://doi.org/10.1186/s40172-014-0017-x

Cobb-Clark, D. A., Kassenboehmer, S. C., & Schurer, S. (2014). Healthy habits: The connection between diet, exercise, and locus of control. *Journal of Economic Behavior & Organization*, *98*, 1–28. https://doi.org/10.1016/j.jebo.2013.10.011

Cobb-Clark, D. A., & Schurer, S. (2013). Two economists' musings on the stability of locus of control. *Economic Journal*, *123*(570). https://doi.org/10.1111/ecoj.12069

Crawford, C., Dearden, L., & Greaves, E. (2011). *Does when you are born matter?The impact ofmonthof birth onchildren's cognitive and non-cognitive skills in England*. Institue for Fiscal Studies. https://www.ifs.org.uk/bns/bn122.pdf

Department for Education, Crawford, C., & Cribb, J. (2012). *Gap year takers: Uptake, trends and long term outcomes*. Department for Education. https://assets.publishing.service.gov.uk/government/uploads/system/uploads/attachment_data/file/219637/DFE-RR252.pdf https://www.gov.uk/government/publications/gap-year-takers-uptake-trends-and-long-term-outcomes

Gladwell, D., Popli, G., & Tsuchiya, A. (2016). *A Dynamic Analysis of Skill Formation and NEET status*. https://cemapre.iseg.ulisboa.pt/educonf/4e3/files/Papers/Popli.pdf

Hankins, M. (2008). The factor structure of the twelve item General Health Questionnaire (GHQ-12): The result of negative phrasing? *Clinical Practice and Epidemiology in Mental Health*, *4*(1), 10. https://doi.org/10.1186/1745-0179-4-10

For peer review only

Mendolia, S., & Walker, I. (2014a). THE EFFECT OF NONCOGNITIVE TRAITS ON HEALTH BEHAVIOURS IN ADOLESCENCE: THE EFFECT OF NONCOGNITIVE TRAITS ON HEALTH BEHAVIOURS IN ADOLESCENCE. *Health Economics*, *23*(9), 1146–1158. https://doi.org/10.1002/hec.3043

Mendolia, S., & Walker, I. (2014b). The effect of personality traits on subject choice and performance in high school: Evidence from an English cohort. *Economics of Education Review*, *43*, 47–65. https://doi.org/10.1016/j.econedurev.2014.09.004

Mendolia, S., & Walker, I. (2015). Youth unemployment and personality traits. *IZA Journal of Labor Economics*, *4*(1). https://doi.org/10.1186/s40172-015-0035-3

Ng-Knight, T., & Schoon, I. (2017). Can Locus of Control Compensate for Socioeconomic Adversity in the Transition from School to Work? *Journal of Youth and Adolescence*, *46*(10), 2114–2128. https://doi.org/10.1007/s10964-017-0720-6

Piatek, R., & Pinger, P. (2016). Maintaining (Locus of) Control? Data Combination for the Identification and Inference of Factor Structure Models. *Journal of Applied Econometrics*, *31*(4), 734–755. https://doi.org/10.1002/jae.2456

Rotter, J. B. (1966). Generalized expectancies for internal versus external control of reinforcement. *Psychological Monographs*, *80*(1), 1–28. https://doi.org/10.1037/h0092976

Rotter, J. B. (1975). Some problems and misconceptions related to the construct of internal versus external control of reinforcement. *Journal of Consulting and Clinical Psychology*, *43*(1), 56–67. https://doi.org/10.1037/h0076301

Wijedasa, D. (2017). 'People like me don't have much of a chance in life': Comparing the locus of control of young people in foster care with that of adoptees, children from disadvantaged backgrounds and children in the general population. *Adoption & Fostering*, *41*(1), 5–19. https://doi.org/10.1177/0308575916684299

For peer review only

STROBE 2007 (v4) Statement—Checklist of items that should be included in reports of *cohort studies*

Section/Topic	Item #	Recommendation	Reported on page #
**Title and abstract**	1	(*a*) Indicate the study's design with a commonly used term in the title or the abstract	1
		(*b*) Provide in the abstract an informative and balanced summary of what was done and what was found	2
**Introduction**			
Background/rationale	2	Explain the scientific background and rationale for the investigation being reported	3
Objectives	3	State specific objectives, including any prespecified hypotheses	4
**Methods**			
Study design	4	Present key elements of study design early in the paper	4
Setting	5	Describe the setting, locations, and relevant dates, including periods of recruitment, exposure, follow-up, and data collection	4
Participants	6	(*a*) Give the eligibility criteria, and the sources and methods of selection of participants. Describe methods of follow-up	4
		(*b*) For matched studies, give matching criteria and number of exposed and unexposed	
Variables	7	Clearly define all outcomes, exposures, predictors, potential confounders, and effect modifiers. Give diagnostic criteria, if applicable	4-5
Data sources/ measurement	8*	For each variable of interest, give sources of data and details of methods of assessment (measurement). Describe comparability of assessment methods if there is more than one group	4-5
Bias	9	Describe any efforts to address potential sources of bias	6
Study size	10	Explain how the study size was arrived at	4
Quantitative variables	11	Explain how quantitative variables were handled in the analyses. If applicable, describe which groupings were chosen and why	4-5
Statistical methods	12	(*a*) Describe all statistical methods, including those used to control for confounding	6
		(*b*) Describe any methods used to examine subgroups and interactions	6
		(*c*) Explain how missing data were addressed	6
		(*d*) If applicable, explain how loss to follow-up was addressed	6
		(*e*) Describe any sensitivity analyses	6
**Results**			

For peer review only

Participants	13*	(a) Report numbers of individuals at each stage of study—eg numbers potentially eligible, examined for eligibility, confirmed eligible, included in the study, completing follow-up, and analysed	4
		(b) Give reasons for non-participation at each stage	
		(c) Consider use of a flow diagram	
Descriptive data	14*	(a) Give characteristics of study participants (eg demographic, clinical, social) and information on exposures and potential confounders	6-8
		(b) Indicate number of participants with missing data for each variable of interest	6-8
		(c) Summarise follow-up time (eg, average and total amount)	4
Outcome data	15*	Report numbers of outcome events or summary measures over time	6
Main results	16	(a) Give unadjusted estimates and, if applicable, confounder-adjusted estimates and their precision (eg, 95% confidence interval). Make clear which confounders were adjusted for and why they were included	9
		(b) Report category boundaries when continuous variables were categorized	
		(c) If relevant, consider translating estimates of relative risk into absolute risk for a meaningful time period	
Other analyses	17	Report other analyses done—eg analyses of subgroups and interactions, and sensitivity analyses	10
**Discussion**			
Key results	18	Summarise key results with reference to study objectives	10
**Limitations**			
Interpretation	20	Give a cautious overall interpretation of results considering objectives, limitations, multiplicity of analyses, results from similar studies, and other relevant evidence	11
Generalisability	21	Discuss the generalisability (external validity) of the study results	11
**Other information**			
Funding	22	Give the source of funding and the role of the funders for the present study and, if applicable, for the original study on which the present article is based	12

*Give information separately for cases and controls in case-control studies and, if applicable, for exposed and unexposed groups in cohort and cross-sectional studies.

Note: An Explanation and Elaboration article discusses each checklist item and gives methodological background and published examples of transparent reporting. The STROBE checklist is best used in conjunction with this article (freely available on the Web sites of PLoS Medicine at http://www.plosmedicine.org/, Annals of Internal Medicine at http://www.annals.org/, and Epidemiology at http://www.epidem.com/). Information on the STROBE Initiative is available at www.strobe-statement.org.

For peer review only

BMJ Open

Heterogeneity in the Association Between Youth Unemployment and Mental Health Later in Life: A Quantile Regression Analysis of Longitudinal Data from English Schoolchildren

Journal:	*BMJ Open*
Manuscript ID	bmjopen-2020-047997.R1
Article Type:	Original research
Date Submitted by the Author:	17-Jun-2021
Complete List of Authors:	Wright, Liam; University College London, Department of Epidemiology and Public Health<br>Head, Jenny; University College London, Department of Epidemiology and Public Health<br>Jivraj, Stephen ; University College London, Departmnent of Epidemiology and Public Health
**Primary Subject Heading**:	Epidemiology
Secondary Subject Heading:	Mental health
Keywords:	MENTAL HEALTH, PUBLIC HEALTH, Depression & mood disorders < PSYCHIATRY

SCHOLARONE™
Manuscripts

BMJ

I, the Submitting Author has the right to grant and does grant on behalf of all authors of the Work (as defined in the below author licence), an exclusive licence and/or a non-exclusive licence for contributions from authors who are: i) UK Crown employees; ii) where BMJ has agreed a CC-BY licence shall apply, and/or iii) in accordance with the terms applicable for US Federal Government officers or employees acting as part of their official duties; on a worldwide, perpetual, irrevocable, royalty-free basis to BMJ Publishing Group Ltd ("BMJ") its licensees and where the relevant Journal is co-owned by BMJ to the co-owners of the Journal, to publish the Work in this journal and any other BMJ products and to exploit all rights, as set out in our licence.

The Submitting Author accepts and understands that any supply made under these terms is made by BMJ to the Submitting Author unless you are acting as an employee on behalf of your employer or a postgraduate student of an affiliated institution which is paying any applicable article publishing charge ("APC") for Open Access articles. Where the Submitting Author wishes to make the Work available on an Open Access basis (and intends to pay the relevant APC), the terms of reuse of such Open Access shall be governed by a Creative Commons licence – details of these licences and which Creative Commons licence will apply to this Work are set out in our licence referred to above.

Other than as permitted in any relevant BMJ Author's Self Archiving Policies, I confirm this Work has not been accepted for publication elsewhere, is not being considered for publication elsewhere and does not duplicate material already published. I confirm all authors consent to publication of this Work and authorise the granting of this licence.

For peer review only

Heterogeneity in the Association Between Youth Unemployment and Mental Health Later in Life: A Quantile Regression Analysis of Longitudinal Data from English Schoolchildren

Liam Wright, Jenny Head and Stephen Jivraj

Department of Epidemiology and Public Health, University College London

Corresponding Author: Liam Wright, 1-19 Torrington Place, London WC1E 7HB, liam.wright.17@ucl.ac.uk

Word count: 3,926

For peer review only

Heterogeneity in the Association Between Youth Unemployment and Mental Health Later in Life: A Quantile Regression Analysis of Longitudinal Data from English Schoolchildren

Abstract

Objectives: An association between youth unemployment and poorer mental health later in life has been found in several countries. Little is known about whether this association is consistent across individuals or differs in strength. We adopt a quantile regression approach to explore heterogeneity in the association between youth unemployment and later mental health along the mental health distribution. **Design:** Prospective longitudinal cohort of secondary schoolchildren in England followed from age 13/14 in 2004 to age 25 in 2015. **Setting:** England, United Kingdom. **Participants:** 7,707 participants interviewed at age 25. **Primary and Secondary Outcome Measures:** 12-Item General Health Questionnaire (GHQ) Likert score, a measure of minor psychiatric morbidity. **Results:** Youth unemployment was related to worse mental health at age 25. The association was several times stronger at deciles of GHQ representing the poorest levels of mental health. This association was only partly attenuated when adjusting for confounding variables and for current employment status. In fully adjusted models not including current employment status, marginal effects at the 50th percentile were 0.73 (CI = -0.05, 1.54, b = 0.11) points, while marginal effects at the 90th percentile were 3.76 (CI = 1.82, 5.83; b = 0.58) points. The results were robust to different combinations of control variables. **Conclusions:** There is heterogeneity in the longitudinal association between youth unemployment and mental health, with associations more pronounced at higher levels of psychological ill-health. Youth unemployment may signal clinically relevant future psychological problems among some individuals.

Keywords: Youth Unemployment; School-to-Work Transition; Mental Health; Life Course Epidemiology; Unemployment Scarring; Quantile Regression.

Strengths and Limitations of this Study

- The study includes longitudinal data from a large sample of young English adults, enabling us to control for several background factors that may explain selection into unemployment.
- To our knowledge, this is the first study on the long-term association between youth unemployment and later mental health using data from adults who entered adulthood during the aftermath of the 2008 Global Financial Crisis.
- We use quantile regression to study whether the association is heterogeneous or consistent across all individuals.
- We run thousands of models to test whether our results are robust to alternative model specifications.
- While we control for several adolescent factors, we use observational data so results may not indicate causality.

For peer review only

INTRODUCTION

The pandemic of coronavirus disease 2019 (COVID-19) looks set to plunge the world into recession and unemployment rates have begun to rise sharply across the globe [1]. A large body of research finds that unemployed individuals have worse mental health and wellbeing than their peers, with evidence from longitudinal and quasi-experimental studies suggesting the association between unemployment and mental health is causal [2,3,though also see 4]. Several theories have been proposed for why unemployment may negatively affect mental health [5], including loss of material and non-material ("latent") benefits of employment [6]. Given this literature, there is concern that the increasing unemployment rates will lead to higher rates of mental ill health [7].

The effect of unemployment on mental health may not be transient. Several studies suggest that unemployment "scars", defined as an effect of (prior) unemployment and poor mental health persisting even upon re-employment [8]. A focus of the literature has been on the long-term effect of youth unemployment specifically, with studies from the United States [9], the United Kingdom [10], Canada [11], and Sweden [12,13] finding associations between early unemployment and subjective wellbeing, depression, and anxiety symptoms measured decades later. These associations remain (where tested) after adjusting for adolescent mental health. This suggests that associations cannot be explained by health-related selection *into* unemployment.

Focusing on youth unemployment (typically defined as unemployment before age 25) is valuable for two reasons. First, unemployment rates are higher among young people and recessions have a disproportionate impact on the job prospects of younger workers [10]. Second, adolescence and young adulthood are important from a life course perspective - unemployment at these ages may have larger long-term effects. Unemployment early in the transition to adulthood has been argued to hinder maturation into normative adult roles and identity [12] and to set off "chains of risk" [14], with initial disadvantage begetting further disadvantage, such as increased future unemployment risk and lower lifetime wages [15,16] - factors which are themselves related to poorer mental health. Adolescence and early adulthood are also sensitive periods of physiological and neurobehavioural development [14,17]. Stressors experienced during this age may have long term effects on physiology and stress responses, negatively impacting lifelong physical and mental health.

While an overall association between youth unemployment and mental health later in life appears well established, little empirical research has explored the reasons behind such a link. Further, little research has studied whether scarring is uniform across all formerly unemployed individuals or whether effects differ in strength or are confined to particular individuals. Yet, this information is useful for directing resources towards the most vulnerable and for providing insight into the life-course processes that may generate long-term effects.

In this paper, we extend the literature by using quantile regression to investigate heterogeneity in the strength of these long-term effects. Unlike in typical ordinary least squares regression, which estimates changes in the conditional mean of a dependent variable, quantile regression estimates changes at specified quantiles of the distribution (i.e. 10th percentile, median, etc.) [18]. Quantile regression thus allows researchers to model change in both the location and shape of the distribution of a dependent variable in response to changes in other variables, and, accordingly, to see whether associations differ in direction or strength across the distribution.

A small number of studies have used quantile regression to investigate heterogeneity in the contemporary association between unemployment and mental health using data from working-age adults [19–21]. Only one study has looked at long-term effects but has looked at life satisfaction, specifically [22]. Each of these studies finds that the negative association between unemployment and mental health is stronger at poorest levels of mental health or wellbeing. This is consistent with studies

For peer review only - http://bmjopen.bmj.com/site/about/guidelines.xhtml

of trajectories in mental wellbeing which show that responses to episodes of unemployment are not uniform, with a minority of individuals appearing more strongly affected [23,24].

We hypothesise that long-term effects will be heterogeneous and that they will be strongest at quantiles representing the poorest levels of mental health. We reason that the strength of the proposed pathways linking youth unemployment to later mental health are likely to differ across individuals. For instance, the consequences of early unemployment for later labour market outcomes (i.e. "chains of risk") is unlikely to be uniform. A recent German study finds that the scarring effect of youth unemployment on future unemployment is primarily driven by a minority of formerly unemployed individuals experiencing particularly long periods of later unemployment [16], while the literature on "compensatory disadvantage" shows that some social adversities can be overcome by individuals with sufficient socioeconomic resources [25]. Further, studies of psychological resilience show that many individuals experience little psychological injury in response to many life stressors [26] – job-loss included [24,27] – while others experience larger and longer-lasting effects. This literature also provides some evidence that those with pre-existing mental health problems are more negatively affected by adverse events [28].

In this paper, we also test whether associations differ by gender. Previous research shows that the contemporary effect of unemployment is typically greater among males [2], though studies using more recent data in contexts of greater female labour force participation do not find differences by gender [29]. We also test whether long-term associations remain after adjusting for current economic activity, in line with original conceptions of scarring as a long-term effect that is independent of later employment status [8].

METHODS

Sample

We use data from Next Steps (formerly, the Longitudinal Study of Young People in England), a cohort of English schoolchildren recruited at age 13/14 in 2003/04 [30]. The cohort were followed annually for seven years from age 13/14 to age 19/20 and surveyed again at age 25 in 2014/15. Participants were interviewed at each wave, with their primary and secondary caregivers also interviewed for the first four waves.

Participants were recruited using a two-stage stratified sampling design with participants selected from a sample of state and independent schools. 15,770 individuals were originally recruited with individuals from ethnic minority backgrounds and schools with a high proportion of students on free school meals over-sampled (response rate: 74%). A sample boost of 352 ethnic minority participants was also added in age 16/17. From 2004-2010, only previous responders were followed across waves and per wave drop-out varied between 8-14%. At the 2014/15 wave, efforts were made to contact previous non-responders. Our sample is all individuals who participated at the age 25 survey (n = 7,707; 47.8% of total sample).

Measures

Mental Health at Age 25

We measure mental health at age 25 using the 12-Item General Health Questionnaire (GHQ-12). The GHQ was developed as a screening tool for minor non-psychotic psychiatric morbidity in the general population [31] and has been found to be a valid measure of mood and anxiety disorders [32]. Items relate to the appearance of new, distressing phenomena and reduced functioning due to psychiatric problems compared with usual experience. Each item is scored on a four-point scale. We use the summed Likert score as our outcome variable. Higher scores indicate worse mental health (range 0-36).

An issue with this measure is that by using usual experience as the reference condition, the GHQ may not adequately capture chronic problems or long-term effects of unemployment [33]. However, a recent

For peer review only - http://bmjopen.bmj.com/site/about/guidelines.xhtml

validation study finds that the GHQ displays good sensitivity for detecting depression [32] and in Supplementary Figure S1, we show visual evidence from the United Kingdom Household Longitudinal Study that GHQ scores are related to long-term depression.

Youth Unemployment

We measure youth unemployment using a binary indicator for whether the participant experienced 6+ months (continuous) unemployment between October 2008 - May 2010 (approximately ages 18-20). This period corresponds to the first twenty months following the summer holiday after the normative end date of further education for this cohort. It also overlaps with the beginning of the Global Financial Crisis, following which youth unemployment rates rose precipitously worldwide [10]. We derive the variable from activity history data which participants provided at each wave on main activities carried out since their previous interview. Activities were selected from a list, with unemployment appearing as "Unemployed" or "Unemployed and looking for work".

We chose a 6+ month cut-off to focus on longer-term periods of youth unemployment, excluding short-term frictional episodes of unemployment that frequently appear during school holidays [34]. In sensitivity analyses, we repeat models using cut-offs of 3+, 9+, and 12+ months, instead. Note, we focus on unemployment, specifically, rather than Not in Employment, Education or Training (NEET) – another measure of exclusion from the labour market – given the more heterogeneous set of experiences that NEET incorporates, such as full-time caring, "gap years", and long-term disability. As Furlong [34] notes, the heterogeneity within NEET "means that both research and policy must begin by disaggregating so as to be able to identify the distinct characteristics and needs of the various sub-groups".

Covariates

We add several control variables to partly account for non-random selection into unemployment. To account for mental health-related selection, we use scores from the 12-item GHQ at ages 14/15 and 16/17. We use the *Caseness* score at age 14/15 (1 if has experienced the symptom more than usual, 0 otherwise; range 0-12). Participants were able to respond "don't know" to each item at this interview (we assume this reflects not experiencing the symptom). We use the Likert score at age 16/17 (range 0-36). The GHQ-12 has been shown to have acceptable validity in adolescent samples [29,30].

To account for physical health-related selection, we control for self-rated health at ages 14/15 and 16/17 (categories: very good, fairly good, not very good, not good at all) and whether the participant had a disability at age 14/15 or 15/16 (categories: no disability; disability, but schooling unaffected; disabled and schooling affected).

We also adjust for gender and ethnicity (categories: White, mixed, Indian, Pakistani, Bangladeshi, Black African, Black Caribbean, other), and to account for differences in social background, we include variables for the participant's family social class (categories: higher, intermediate, routine/manual, long-term unemployed) and highest parental education (further education or higher vs secondary or lower), both measured when the participant was aged 13/14. We also include a measure of neighbourhood deprivation at age 14/15, the Index of Multiple Deprivation 2004 (IMD), which is produced by the UK Government and captures local area deprivation across seven dimensions (income, employment, health, education, barriers to housing and services, living environment and crime). We use quintiles of IMD, with higher quintiles indicating greater deprivation [35].

To account for differences in human capital, we control for educational attainment at age 25 (six categories: National Vocational Qualification levels 1-5, no qualifications). (Education data is not publicly available for ages prior to this.) Social adjustment and non-cognitive skills are likely to cause both labour market outcomes and mental health. As proxies for some of these, we include variables for positive attitude to school (age 14/15; summed response to 12-item measure, range 0-48), risk behaviours (age 13/14; summed response to 8-item measure on anti-social behaviour, alcohol, smoking

For peer review only - http://bmjopen.bmj.com/site/about/guidelines.xhtml

and drug use in previous 12 months, range 0-8), and bullying victimisation (number of waves reported being bullied in prior 12 months, age 13/14 – 15/16, range 0-3). We also include a measure of *internal* locus of control (beliefs about the extent to which the participant controls their life), produced from confirmatory factor analysis of six questionnaire item. Further detail on this measure is provided in the Supplementary Information.

Current economic activity at age 25 was derived from a question on main activity (categories: employed, education, unemployed, inactive).

Statistical Analysis

We use quantile regression to estimate the association between youth unemployment and GHQ scores at age 25 at each decile of the outcome variable. We run unadjusted models, covariate adjusted models, and covariate adjusted models with current economic activity as a further control variable. We use bootstrapping to estimate confidence intervals (500 replications) and apply survey weights supplied with the data to account for differences in the likelihood of recruitment to, and exit from, the study. To account for item-missingness, we use multiple imputation (m = 28, burn-in=10), adding the survey weights as linear terms in the imputation models to partly account for weighting in our final analysis [36]. We use data from all sample members interviewed at age 25 in the imputations, but discard participants with missing outcome data in the quantile regressions (final sample n = 7,363). We conduct multiple imputations separately for male and female participants.

A potential issue with our analysis is that some control variables may bias associations. For instance, education may be a mediator and the use of proxies for social adjustment and non-cognitive skills could open backdoor paths between youth unemployment and later mental health [37]. Therefore, as a sensitivity analysis, we run a random selection of 20,000 models drawn from all possible combinations of the covariates defined above. To minimise the computational cost, we use only a single imputed dataset for these regressions.

Another potential issue is that we have included few controls for adolescent family background and socio-economic position, which may cause residual confounding. Therefore, as another sensitivity analysis, we repeat our main analysis including further adjustment for family financial difficulties ("Thinking of how your household is managing on your total household income at the moment, would you say it was...": managing quite well, able to save or spend on leisure; just getting by, unable to save if wanted to; getting into difficulties), number of household children (categorical: 1, 2, 3, 4, 5+), and household type (two parent household, single parent household). Each of these variables was measured at age 13/14 from parental reports. Finally, as noted above, as a final sensitivity analysis, we repeat the main analysis model (no adjustment for current status) using different cut-offs to define youth unemployment: 3+, 9+, and 12+ months unemployment.

Multiple imputations [38] and data analysis were carried out in R version 3.6.3 [39]. The code to replicate this analysis is available at https://osf.io/3gwap/. Next Steps data are available through the UK Data Service [30].

Ethics Approval Statement

Ethical approval for Next Steps has been gained from the National Health Service (NHS) Research Ethics Committee (REC) system (14/LO/0096). This is a secondary data analysis of Next Steps so ethical approval was not required.

Patient and Public Involvement

The public were not involved in the design, or conduct, or reporting, or dissemination plans of our research.

For peer review only - http://bmjopen.bmj.com/site/about/guidelines.xhtml

RESULTS

Descriptive Statistics

Descriptive statistics are displayed in Table 1. Individuals with 6+ months unemployment between ages 18-20 differ from those with less unemployment experience along a number of dimensions, including mental and physical health and socio-economic background. This is consistent with other studies showing that selection into youth unemployment is not random [40,41]. There is little difference according to GHQ scores at age 16/17, which may suggest measurement error in this variable.

Table 1: Descriptive statistics.

		Unweighted Observed Data			Weighted Imputed Data	
	Variable	<6 + Months Unemployment	6+ Months Unemployment	% Missing	<6 + Months Unemployment	6+ Months Unemployment
	n	6,589 (91.76%)	592 (8.24%)		6,599.90 (88.03%)	897.13 (11.97%)
	GHQ-12 @ Age 25	11.53 (6)	13.42 (7.59)	4.46%	11.71 (6.19)	13.62 (7.65)*
Gender	Male	2,799 (42.48%)	317 (53.55%)	0%	3,183.39 (48.23%)	538.54 (60.03%)*
	Female	3,790 (57.52%)	275 (46.45%)		3,416.51 (51.77%)	358.59 (39.97%)
	IMD	22.56 (16.76)	30.42 (18.13)	8.78%	22.18 (16.18)	30.08 (18.26)*
	Locus of Control	0.05 (0.97)	-0.51 (1.14)	12.72%	-0.05 (1.01)	-0.64 (1.13)*
Current Economic Activity	Employed	5,569 (85.19%)	370 (62.93%)	1.12%	5,499.25 (83.32%)	521.59 (58.14%)*
	Education	307 (4.7%)	11 (1.87%)		271.68 (4.12%)	12.64 (1.41%)
	Inactive	402 (6.15%)	92 (15.65%)		523.71 (7.94%)	182.18 (20.31%)
	Unemployed	259 (3.96%)	115 (19.56%)		305.25 (4.63%)	180.73 (20.15%)
	GHQ-12 @ Age 14/15	1.74 (2.53)	2.06 (2.76)	13.16%	1.76 (2.58)	2.1 (2.72)*
	GHQ-12 @ Age 16/17	10.5 (5.92)	10.29 (6.48)	20.85%	10.34 (5.98)	10.3 (6.53)
Self-Rated Health @ Age 14/15	Very Good	2,576 (45.54%)	166 (34.51%)	15.22%	2,902.29 (43.97%)	306.18 (34.13%)*
	Fairly Good	2,903 (51.32%)	288 (59.88%)		3,401.69 (51.54%)	520.99 (58.07%)
	Not Very Good	150 (2.65%)	21 (4.37%)		238.64 (3.62%)	58.41 (6.51%)
	Not Good at All	28 (0.49%)	6 (1.25%)		57.27 (0.87%)	11.55 (1.29%)
Self-Rated Health @	Very Good	2,922 (51.97%)	215 (43.26%)	15.67%	3,315.12 (50.23%)	384.90 (42.9%)*

Age 16/17	Fairly Good	2,308 (41.05%)	246 (49.5%)		2,736.67 (41.47%)	440.17 (49.06%)
	Not Very Good	329 (5.85%)	29 (5.84%)		440.10 (6.67%)	57.85 (6.45%)
	Not Good at All	63 (1.12%)	7 (1.41%)		108.00 (1.64%)	14.21 (1.58%)
Disabled	No	5,619 (87.7%)	466 (80.48%)	2.76%	5,633.49 (85.36%)	701.73 (78.22%)*
	Yes, school not affected	476 (7.43%)	47 (8.12%)		556.52 (8.43%)	74.86 (8.34%)
	Yes, school affected	312 (4.87%)	66 (11.4%)		409.89 (6.21%)	120.54 (13.44%)
	Risk Behaviours	0.76 (1.31)	1.18 (1.65)	12.24%	0.96 (1.5)	1.47 (1.84)*
	Attitude to School	33.49 (7.19)	30.1 (8.02)	10.72%	32.28 (7.72)	28.33 (8.26)*
	# Waves Bullied, 1-3	1.34 (1.15)	1.61 (1.15)	15.47%	1.47 (1.15)	1.72 (1.13)*
Qualifications	NVQ 5	1,167 (17.71%)	19 (3.21%)	0%	927.05 (14.05%)	16.90 (1.88%)*
	NVQ 4	1,846 (28.02%)	57 (9.63%)		1,611.71 (24.42%)	65.08 (7.25%)
	NVQ 3	1,355 (20.56%)	72 (12.16%)		1,147.44 (17.39%)	70.78 (7.89%)
	NVQ 2	1,350 (20.49%)	189 (31.93%)		1,613.46 (24.45%)	252.11 (28.1%)
	NVQ 1	497 (7.54%)	165 (27.87%)		815.63 (12.36%)	338.13 (37.69%)
	No/Other Qual	374 (5.68%)	90 (15.2%)		484.60 (7.34%)	154.14 (17.18%)
Parental NS-SEC	Higher	2,393 (41.27%)	117 (22.72%)	12.33%	2,503.64 (37.93%)	169.41 (18.88%)*
	Intermediate	1,208 (20.83%)	87 (16.89%)		1,393.86 (21.12%)	127.20 (14.18%)
	Routine	1,889 (32.58%)	259 (50.29%)		2,363.27 (35.81%)	516.22 (57.54%)
	LTU	308 (5.31%)	52 (10.1%)		339.13 (5.14%)	84.30 (9.4%)
Parental Education	Degree	1,080 (19.44%)	52 (10.28%)	15.71%	1,098.52 (16.64%)	67.68 (7.54%)*
	Other HE	939 (16.9%)	64 (12.65%)		1,042.91 (15.8%)	91.76 (10.23%)
	A-Level	973 (17.51%)	77 (15.22%)		1,141.75 (17.3%)	128.93 (14.37%)
	GCSE A-C	1,381 (24.86%)	128 (25.3%)		1,844.99 (27.95%)	258.99 (28.87%)

	Other/None	1,183 (21.29%)	185 (36.56%)		1,471.73 (22.3%)	349.78 (38.99%)
Ethnicity	White	4,498 (68.27%)	412 (69.59%)	0%	5,580.25 (84.55%)	776.50 (86.55%)*
	Mixed	298 (4.52%)	30 (5.07%)		165.52 (2.51%)	23.70 (2.64%)
	Indian	439 (6.66%)	15 (2.53%)		146.52 (2.22%)	8.73 (0.97%)
	Pakistani	354 (5.37%)	38 (6.42%)		158.68 (2.4%)	21.96 (2.45%)
	Bangladeshi	289 (4.39%)	41 (6.93%)		76.53 (1.16%)	12.84 (1.43%)
	Black African	197 (2.99%)	28 (4.73%)		95.29 (1.44%)	26.65 (2.97%)
	Black Caribbean	278 (4.22%)	12 (2.03%)		162.33 (2.46%)	10.42 (1.16%)
	Other	236 (3.58%)	16 (2.7%)		214.77 (3.25%)	16.34 (1.82%)
Financial Difficulties	Managing Well	3,407 (53.28%)	203 (35.74%)	4%	3,336.00 (51.37%)	272.56 (31.38%)*
	Getting By	2,622 (41%)	309 (54.4%)		2,791.23 (42.98%)	517.32 (59.55%)
	Having Difficulties	366 (5.72%)	56 (9.86%)		367.05 (5.65%)	78.83 (9.07%)
# Household Children	1	1,428 (22.03%)	139 (24.13%)	2.6%	1,494.96 (23.02%)	237.04 (27.29%)*
	2	2,811 (43.36%)	210 (36.46%)		2,857.47 (44%)	312.58 (35.98%)
	3	1,423 (21.95%)	132 (22.92%)		1,399.61 (21.55%)	196.44 (22.61%)
	4	576 (8.88%)	47 (8.16%)		557.35 (8.58%)	66.61 (7.67%)
	5+	245 (3.78%)	48 (8.33%)		184.88 (2.85%)	56.04 (6.45%)
Household Type	Two Parent	5,202 (80.12%)	396 (68.39%)	2.4%	4,821.42 (74.24%)	514.38 (59.21%)*
	Single Parent	1,291 (19.88%)	183 (31.61%)		1,672.87 (25.76%)	354.33 (40.79%)

* $p < 0.05$ from Meng and Rubin [42] Likelihood Ratio test.

Quantile Regression

The results of the quantile regressions are presented in Table 2. Except at the lowest quantiles of GHQ scores (which indicate good mental health), youth unemployment is associated with worse mental health at age 25 (Column A). Associations are only partly attenuated controlling for adolescent mental health (Column B) or adolescent mental health and other background characteristics (Column C). Consistent with our hypothesis, associations are stronger at quantiles representing greater levels of poor mental health. For example, the predicted GHQ score at the 50th percentile among those with 6+ months unemployment is 0.73 (CI = -0.05, 1.54, b = 0.11) points greater than at the 50th percentile among those with less than 6 months unemployment. At the 90th percentiles, the difference is 3.76 (CI = 1.82, 5.83;

For peer review only - http://bmjopen.bmj.com/site/about/guidelines.xhtml

b = 0.58) points. Further adjustment for current employment status partly attenuates these estimates (Column D), but associations remain substantial at highest quantiles. The results for models in Columns C and D are displayed graphically in Figure 1. (Full regression results are displayed in Supplementary Tables S1 to S6.)

Table 2: Main Regression Results

Quantile	(A)	(B)	(C)	(D)	(E)	(F)
Q10	0<br>(-1, 1)	0<br>(-0.79, 0.57)	0.15<br>(-0.67, 0.98)	0.1<br>(-0.78, 0.95)	0.83<br>(-0.26, 1.78)	-0.49<br>(-1.55, 0.87)
Q20	0<br>(0, 1)	0.36<br>(-0.31, 0.93)	0.49<br>(-0.2, 1.16)	0.42<br>(-0.3, 1.03)	0.79<br>(-0.13, 1.6)	0.31<br>(-1.07, 1.35)
Q30	1<br>(0, 2)	0.48<br>(-0.15, 1.2)	0.51<br>(-0.19, 1.2)	0.28<br>(-0.4, 0.94)	0.61<br>(-0.32, 1.52)	0.49<br>(-0.52, 1.43)
Q40	1<br>(0, 2)	0.67<br>(-0.07, 1.57)	0.55<br>(-0.16, 1.28)	0.22<br>(-0.46, 0.92)	0.59<br>(-0.35, 1.65)	0.46<br>(-0.53, 1.46)
Q50	1<br>(0, 2)	1.1<br>(0.35, 1.79)	0.73<br>(-0.05, 1.54)	0.22<br>(-0.59, 1.09)	0.75<br>(-0.33, 1.92)	0.58<br>(-0.64, 1.96)
Q60	2<br>(0, 2)	1.57<br>(0.67, 2.48)	1.1<br>(0.14, 2.32)	0.52<br>(-0.4, 1.73)	0.99<br>(-0.28, 2.51)	1.19<br>(-0.32, 3.03)
Q70	3<br>(2, 4)	2.41<br>(1.27, 3.85)	2.03<br>(0.72, 3.36)	1.55<br>(0.07, 2.82)	1.76<br>(-0.07, 4.05)	2.11<br>(0.18, 3.84)
Q80	4<br>(3, 6)	4<br>(1.95, 6.2)	2.95<br>(1.12, 4.92)	2.24<br>(0.73, 4.02)	3.22<br>(0.47, 6.4)	2.3<br>(0.33, 4.36)
Q90	5<br>(3, 8)	6.2<br>(3.22, 7.62)	3.76<br>(1.82, 5.83)	2.69<br>(0.92, 4.42)	4.16<br>(1.21, 7.14)	2.48<br>(0.34, 4.59)
Observations	7,363	7,363	7,363	7,363	3,196	4,167
Imputations	28	28	28	28	28	28

Quantile regression of GHQ-12 Likert scores at age 25 on 6+ months unemployment experience between ages 18-20. (A) Bivariate association. (B) adjustment for GHQ-12 scores at ages 14/15 and 16/17. (C) Model B plus adjustment for disability and self-rated health, educational attainment, risk behaviours, attitude to school and bullying victimisation, IMD quintile, parental socio-economic class and education, gender, locus of control, and ethnicity. (D) Model C plus adjustment for current economic activity. (E) Model C estimated for males only. (F) Model C estimated for females only.

INSERT FIGURE 1 HERE

Predicted GHQ-12 scores according to youth unemployment experience derived from fully adjusted model (excluding current activity) in Column C are displayed in Figure 2. (Other covariates kept at the sample means or modes.) Over 30% of those with 6+ months unemployment are predicted to have GHQ Likert scores above 15, 10% points more than those with less youth unemployment experience. For context, one recent Swedish validation study found a cut-off of 11/12 points provides adequate sensitivity and specificity for detecting depression [32].

INSERT FIGURE 2 HERE

For peer review only - http://bmjopen.bmj.com/site/about/guidelines.xhtml

Figure 3 shows the results of the adjusted model not including current economic activity stratifying by gender. Stronger associations are again found at poorest levels of mental health, and effect sizes are similar in each gender. The results are also displayed in Columns E and F of Table 2 (male and female, respectively). Full regression results are displayed in the Supplementary Information.

INSERT FIGURE 3 HERE

Sensitivity Analysis

The results of the 20,000 models, representing a random selection of all possible combinations of the covariates, are displayed in Figure 4. Each line represents a single model run for each decile of GHQ-12 scores at age 25. Overlaid on the figure are the results of the main fully adjusted models, with and without further control for current economic status. The results are very consistent regardless of the set of control variables used.

INSERT FIGURE 4 HERE

The results of models included further adjustment for adolescent socio-economic position and family background are displayed in Supplementary Figure S2, with results from the main analysis (i.e., Column C, Table 2) also shown for comparison. Again, results are very similar, regardless of the control variables used. The results of the sensitivity analysis using different cut-offs to define youth unemployment are shown in Supplementary Figure S3. The same qualitative pattern of stronger associations at higher quantiles is observed in each case, but associations are typically stronger when longer durations are used to define unemployment, though confidence intervals are overlapping.

For peer review only

DISCUSSION

Using a quantile regression approach, we find that, on average, individuals who were continuously unemployed for 6+ months between ages 18-20 have poorer mental health at age 25, but that there is substantial heterogeneity with associations being more pronounced at quantiles of the distribution representing the worse mental health. Effect sizes at poorer levels of mental health are substantial and clinically significant: a higher proportion of those who were unemployed as youths exceed suggested thresholds for detecting depression with GHQ-12 Likert scores [32]. Associations remain after adjusting for adolescent mental and physical health, suggesting results may not be explained by health-related selection into unemployment, though there appears to be measurement error in the instruments we use. Associations were not fully attenuated when adjusting for current employment status and were robust to using different combinations of control variables. Point estimates were larger for males than females, particularly at higher quantiles of GHQ-12 scores.

Our results are consistent with previous studies which show that youth unemployment is related to worse mental health later in life [9–13,43] irrespective of later employment outcomes [12], but we extend the literature by showing that associations differ substantially across individuals. A natural next question is to identify individuals who appear to be most impacted by youth unemployment. Associations in our sample were stronger among males than females (though confidence intervals overlapped), which is consistent with the results of a recent Finnish study [15]. Men are generally found to experience worse mental health effects from (contemporary) unemployment [2], though whether this applies to situations where similar numbers of males and females participate in the labour market has been questioned [29]. Further, studies also show differences in *economic* scarring effects by gender, which could also contribute to long-term negative mental health impacts, but the direction of this difference is not consistent across studies [44,45].

Besides gender, a small number of moderating factors have been explored in the existing literature. Lee et al. [9] find little evidence of differences by neighbourhood deprivation (though analyses appear underpowered), while Bijlsma and colleagues [15] find evidence that scarring effects are stronger among males with low education, and Clark and Lepinteur [22] find evidence of stronger associations

between early unemployment and later life satisfaction among males from disadvantaged households. Other possible moderators that deserve further exploration are personality traits, such as locus of control. Locus of control has been linked to psychological adjustment following adversity [46] [though, also see 47] and also to active job-seeking behaviours among the unemployed [48]. Identifying vulnerable groups would have important implications for the targeting of policy interventions.

Another avenue for future research is to identify the mechanisms through which scarring effects may operate. Biljsma and colleagues [15] find evidence that the association between unemployment and later depression is partly mediated by income, but we know of no study that has assessed whether unemployment during adolescence alters neurobehavioural development. These pathways have different implications for designing policy to reduce scarring effects and potentially for identifying vulnerable groups. At present we are unable to say whether the heterogeneity observed reflects individual differences in psychological resilience or propensities for unemployment to engender future socioeconomic adversity.

Our study has a number of limitations. We use observational data and as such, our results cannot be taken as indicating causality. Associations may reflect health-related selection into unemployment, though we adjust for adolescent mental and physical health and differences in GHQ scores were observed across most of the distribution – mental health-related selection into unemployment is arguably only a plausible explanation where psychological morbidity is severe enough to preclude job-finding or job-holding. Nevertheless, there are several other factors that are unobserved or imperfectly measured in our study, such as personality traits and human capital, which may explain associations. Controlling for current economic activity is likely to have induced collider bias. Estimates may have appeared more attenuated than was accurate. While we included attrition weights in our analysis, over half of the sample did not participate at the age 25 survey. If participants who were most harmed by unemployment were more likely to drop-out of the survey, this would also bias results [though, see, 43].

Another limitation is that, by focusing on unemployment during ages 18-20, we exclude from the unemployed group those that only enter the labour market at a later age (i.e., those that went to university). Previously research suggests scarring effects are smaller among this group [15]. Our use of the GHQ as a measure of mental health could also have biased results as items inquire about current symptoms vis-à-vis typical experience. This may not appropriately capture chronic psychological ill health, though a recent validation study finds that the GHQ displays good sensitivity for detecting depression [32].

Our results may be specific to the cohort we studied. The period in which we measure youth unemployment overlapped with the 2008 Global Financial Crisis, following which youth unemployment rates rose worldwide [1]. The long-term consequences of unemployment may be smaller following a recession as prospective employers may look on periods of unemployment less unfavourably [49], and so social chains of risk may be weaker. Further, outcomes for those that do not become unemployed - who we use to measure long-term effects - may simultaneously get worse as labour markets get weaker [50].

Finally, the results may be specific to the age we studied. Participants were early in their labour market careers when we measured mental health outcomes. Differences between the youth unemployed and their peers may grow through time (e.g., following promotions) or become progressively more important if, for instance, individuals are less able to rely on financial help from their families as they age. Alternatively, differences could dissipate if individuals are able to recover from early labour market adversity and it is “chains of risk” that explain our results. A useful extension to this study would be to repeat the analysis at later ages and in other countries with different labour market institutions.

Figures:

For peer review only - http://bmjopen.bmj.com/site/about/guidelines.xhtml

Figure 1: Association between 6+ months youth unemployment between ages 18-20 and GHQ-12 Likert scores at age 25, by decile of GHQ-12. (a) adjusted for adolescent mental health, disability and self-rated health, educational attainment, risk behaviours, attitude to school and bullying victimisation, IMD quintile, parental socio-economic class and education, gender, locus of control, and ethnicity. (b) additionally adjusted for current status.

Figure 2: Predicted age 25 GHQ-12 Likert scores by youth unemployment experience, by decile of GHQ-12. Models include adjustment for adolescent mental health, disability and self-rated health, educational attainment, risk behaviours, attitude to school and bullying victimisation, IMD quintile, parental socio-economic class and education, gender, locus of control, and ethnicity. Predicted values derived used sample means for covariates.

Figure 3: Association between 6+ months youth unemployment between ages 18-20 and GHQ-12 Likert scores at age 25, by gender and decile of GHQ-12. Adjusted for adolescent mental health, disability and self-rated health, educational attainment, risk behaviours, attitude to school and bullying victimisation, IMD quintile, parental socio-economic class and education, gender, locus of control, and ethnicity.

Figure 4: Association between 6+ months youth unemployment between ages 18-20 and GHQ-12 Likert scores at age 25, by decile of GHQ-12. 20,000 models drawn from all possible combinations of covariates. Black lines are results of fully adjusted models, including (dashed) or not including (solid) further adjustment for current economic activity.

Funding: This work was funded by the Economic and Social Research Council through the UCL, Bloomsbury and East London Doctoral Training Partnership (ES/P000592/1). The funding was awarded to Liam Wright.

Conflicts of Interest: None declared.

Acknowledgements: None.

Author Contributions: All authors contributed to the design of the study. LW carried out the analysis and wrote the manuscript. JH and SJ provided detailed comments on the manuscript.

Data Statement: The code to replicate this analysis is available at https://osf.io/3gwap/. Next Steps data are available through the UK Data Service (http://doi.org/10.5255/UKDA-SN-5545-7).

For peer review only

REFERENCES

1 Bell DNF, Blanchflower DG. US AND UK LABOUR MARKETS BEFORE AND DURING THE COVID-19 CRASH. *NIER* 2020;**252**:R52–69. doi:10.1017/nie.2020.14

2 Paul KI, Moser K. Unemployment impairs mental health: Meta-analyses. *Journal of Vocational Behavior* 2009;**74**:264–82. doi:10.1016/j.jvb.2009.01.001

3 Gathergood J. An Instrumental Variable Approach To Unemployment, Psychological Health and Social Norm Effects. *Health Economics* 2013;**22**:643–54. doi:10.1002/hec.2831

4 Schmitz H. Why are the unemployed in worse health? The causal effect of unemployment on health. *Labour Economics* 2011;**18**:71–8. doi:10.1016/j.labeco.2010.08.005

5 Creed PA, Bartrum D. Explanations for deteriorating wellbeing in unemployed people: Specific unemployment theories and beyond. In: *Unemployment and health – International & interdisciplinary perspectives*. 2006. https://www.researchgate.net/publication/29462374_Explanations_for_Deteriorating_Wellbeing_in_Unemployed_People_Specific_Unemployment_Theories_and_Beyond

6 Jahoda M. *Employment and Unemployment: A Social-Psychological Analysis*. Cambridge: : Cambridge University Press 1982.

7 Blustein DL, Duffy R, Ferreira JA, *et al.* Unemployment in the time of COVID-19: A research agenda. *Journal of Vocational Behavior* 2020;**119**:103436. doi:10.1016/j.jvb.2020.103436

8 Clark AE, Georgellis Y, Sanfey P. Scarring: The psychological impact of past unemployment. *Economica* 2001;**68**:221–41. doi:10.1111/1468-0335.00243

9 Lee JO, Jones TM, Yoon Y, *et al.* Young Adult Unemployment and Later Depression and Anxiety: Does Childhood Neighborhood Matter? *Journal of Youth and Adolescence* 2019;**48**:30–42. doi:10.1007/s10964-018-0957-8

10 Bell DNF, Blanchflower DG. Young people and the great recession. *Oxford Review of Economic Policy* 2011;**27**:241–67. doi:10.1093/oxrep/grr011

11 Wheaton B, Reid S. The role of timing vs. duration in the cumulative work history effects of job exits and nonemployment on women's mental health. *Advances in Life Course Research* 2008;**13**:195–232. doi:10.1016/S1040-2608(08)00008-7

12 Strandh M, Winefield A, Nilsson K, *et al.* Unemployment and mental health scarring during the life course. *European Journal of Public Health* 2014;**24**:440–5. doi:10.1093/eurpub/cku005

13 Thern E, de Munter J, Hemmingsson T, *et al.* Long-term effects of youth unemployment on mental health: Does an economic crisis make a difference? *Journal of Epidemiology and Community Health* 2017;**71**:344–9. doi:10.1136/jech-2016-208012

14 Ben-Shlomo Y, Kuh D. A life course approach to chronic disease epidemiology: conceptual models, empirical challenges and interdisciplinary perspectives. *International Journal of Epidemiology* 2002;**31**:285–93. doi:10.1093/intjepid/31.2.285

15 Bijlsma MJ, Tarkiainen L, Myrskylä M, *et al.* Unemployment and subsequent depression: A mediation analysis using the parametric G-formula. *Social Science and Medicine* 2017;**194**:142–50. doi:10.1016/j.socscimed.2017.10.011

For peer review only - http://bmjopen.bmj.com/site/about/guidelines.xhtml

16 Schmillen A, Umkehrer M. The scars of youth: Effects of early-career unemployment on future unemployment experience. *International Labour Review* 2017;**156**:465–94. doi:10.1111/ilr.12079

17 Fuhrmann D, Knoll LJ, Blakemore SJ. Adolescence as a Sensitive Period of Brain Development. *Trends in Cognitive Sciences* 2015;**19**:558–66. doi:10.1016/j.tics.2015.07.008

18 Koenker R, Bassett G. Regression Quantiles. *Econometrica* 1978;**46**:33. doi:10.2307/1913643

19 Binder M, Coad A. Heterogeneity in the Relationship Between Unemployment and Subjective Wellbeing: A Quantile Approach. *Economica* 2015;**82**:865–91. doi:10.1111/ecca.12150

20 Binder M, Coad A. Unemployment impacts differently on the extremes of the distribution of a comprehensive well-being measure. *Applied Economics Letters* 2015;**22**:619–27. doi:10.1080/13504851.2014.962219

21 Graham C, Nikolova M. Bentham or Aristotle in the Development Process? An Empirical Investigation of Capabilities and Subjective Well-Being. *World Development* 2015;**68**:163–79. doi:10.1016/j.worlddev.2014.11.018

22 Clark AE, Lepinteur A. The causes and consequences of early-adult unemployment: Evidence from cohort data. *Journal of Economic Behavior & Organization* 2019;**166**:107–24. doi:10.1016/j.jebo.2019.08.020

23 Georgellis Y, Gregoriou A, Healy J, *et al.* Unemployment and life satisfaction: a non-linear adaptation process. *Int J of Manpower* 2008;**29**:668–80. doi:10.1108/01437720810908956

24 Galatzer-Levy IR, Bonanno GA, Mancini AD. From marianthal to latent growth mixture modeling: A return to the exploration of individual differences in response to unemployment. *Journal of Neuroscience, Psychology, and Economics* 2010;**3**:116–25. doi:10.1037/a0020077

25 Bernardi F, Grätz M. Making Up for an Unlucky Month of Birth in School: Causal Evidence on the Compensatory Advantage of Family Background in England. *SocScience* 2015;**2**:235–51. doi:10.15195/v2.a12

26 Galatzer-Levy IR, Huang SH, Bonanno GA. Trajectories of resilience and dysfunction following potential trauma: A review and statistical evaluation. *Clinical Psychology Review* 2018;**63**:41–55. doi:10.1016/j.cpr.2018.05.008

27 Etilé F, Frijters P, Johson DW, *et al.* Modelling Heterogeneity in the Resilience to Major Socioeconomic Life Events. Paris School of Economics 2017. https://halshs.archives-ouvertes.fr/halshs-01485989/document

28 Nandi A, Tracy M, Beard JR, *et al.* Patterns and Predictors of Trajectories of Depression after an Urban Disaster. *Annals of Epidemiology* 2009;**19**:761–70. doi:10.1016/j.annepidem.2009.06.005

29 Hammarström A, Gustafsson PE, Janlert U, *et al.* It's no surprise! Men are not hit more than women by the health consequences of unemployment in the Northern Swedish Cohort. *Scandinavian Journal of Public Health* 2011;**39**:187–93. doi:10.1177/1403494810394906

30 Centre for Longitudinal Studies. *Next Steps: Sweeps 1-8, 2004-2016 (SN: 5545)*. 14th ed. UK Data Service 2018. http://doi.org/10.5255/UKDA-SN-5545-6

31 Goldberg DP, Hillier VF. A scaled version of the General Health Questionnaire. *Psychological Medicine* 1979;**9**:139–45. doi:10.1017/S0033291700021644

For peer review only - http://bmjopen.bmj.com/site/about/guidelines.xhtml

32 Lundin A, Hallgren M, Theobald H, *et al.* Validity of the 12-item version of the General Health Questionnaire in detecting depression in the general population. *Public Health* 2016;**136**:66–74. doi:10.1016/j.puhe.2016.03.005

33 Morrell S, Taylor R, Quine S, *et al.* A cohort study of unemployment as a cause of psychological disturbance in Australian youth. *Social Science and Medicine* 1994;**38**:1553–64. doi:10.1016/0277-9536(94)90117-1

34 Furlong A. Not a very NEET solution. *Work, employment and society* 2006;**20**:553–69. doi:10.1177/0950017006067001

35 Ministry of Housing, Communities and Local Government. Index of Multiple Deprivation 2004. 2014.https://data.gov.uk/dataset/59599787-bd50-4500-a409-fc586260dbbd/index-of-multiple-deprivation-2004 (accessed 25 Oct 2019).

36 Carpenter JR, Kenward MG. *Multiple imputation and its application*. 1st ed. Chichester, West Sussex, UK: : John Wiley & Sons 2013.

37 VanderWeele TJ. Principles of confounder selection. *Eur J Epidemiol* 2019;**34**:211–9. doi:10.1007/s10654-019-00494-6

38 van Buuren S, Groothuis-Oudshoorn K. mice: Multivariate Imputation by Chained Equations in R. *J Stat Soft* 2011;**45**. doi:10.18637/jss.v045.i03

39 R Core Team. *R: A Language and Environment for Statistical Computing*. Vienna, Austria: : R Foundation for Statistical Computing 2020. https://www.R-project.org/

40 Egan M, Daly M, Delaney L. Adolescent psychological distress, unemployment, and the Great Recession: Evidence from the National Longitudinal Study of Youth 1997. *Social Science and Medicine* 2016;**156**:98–105. doi:10.1016/j.socscimed.2016.03.013

41 Caspi A, Wright BRE, Moffitt TE, *et al.* Early Failure in the Labor Market: Childhood and Adolescent Predictors of Unemployment in the Transition to Adulthood. *American Sociological Review* 1998;**63**:424. doi:10.2307/2657557

42 Meng X-L, Rubin DB. Performing likelihood ratio tests with multiply-imputed data sets. *Biometrika* 1992;**79**:103–11. doi:10.1093/biomet/79.1.103

43 Wright L, Head JA, Jivraj S. How robust is the association between youth unemployment and later mental health? An analysis of longitudinal data from English schoolchildren. *Occup Environ Med* 2021;:oemed-2021-107473. doi:10.1136/oemed-2021-107473

44 Mooi-Reci I, Ganzeboom HB. Unemployment scarring by gender: Human capital depreciation or stigmatization? Longitudinal evidence from the Netherlands, 1980â€“2000. *Social Science Research* 2015;**52**:642–58. doi:10.1016/j.ssresearch.2014.10.005

45 Manzoni A, Mooi-Reci I. The cumulative disadvantage of unemployment: Longitudinal evidence across gender and age at first unemployment in Germany. *PLOS ONE* 2020;**15**:e0234786. doi:10.1371/journal.pone.0234786

46 Kammeyer-Mueller JD, Judge TA, Scott BA. The Role of Core Self-Evaluations in the Coping Process. *Journal of Applied Psychology* 2009;**94**:177–95. doi:10.1037/a0013214

For peer review only - http://bmjopen.bmj.com/site/about/guidelines.xhtml

47 Buddelmeyer H, Powdthavee N. Can having internal locus of control insure against negative shocks? Psychological evidence from panel data. *Journal of Economic Behavior & Organization* 2016;**122**:88–109. doi:10.1016/j.jebo.2015.11.014

48 Cobb-Clark DA. Locus of control and the labor market. *IZA Journal of Labor Economics* 2015;**4**:3. doi:10.1186/s40172-014-0017-x

49 Kroft K, Lange F, Notowidigdo MJ. Duration dependence and labor market conditions: Evidence from a field experiment. *Quarterly Journal of Economics* 2013;**128**. doi:10.1093/qje/qjt015

50 Green F. Unpacking the misery multiplier: How employability modifies the impacts of unemployment and job insecurity on life satisfaction and mental health. *Journal of Health Economics* 2011;**30**:265–76. doi:10.1016/j.jhealeco.2010.12.005

For peer review only

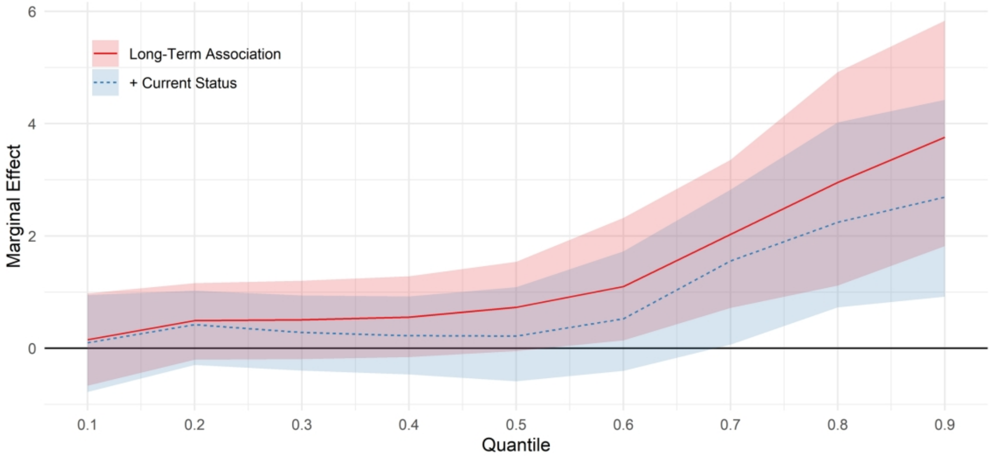

Figure 1: Association between 6+ months youth unemployment between ages 18-20 and GHQ-12 Likert scores at age 25, by decile of GHQ-12. (a) adjusted for adolescent mental health, disability and self-rated health, educational attainment, risk behaviours, attitude to school and bullying victimisation, IMD quintile, parental socio-economic class and education, gender, locus of control, and ethnicity. (b) additionally adjusted for current status.

209x98mm (300 x 300 DPI)

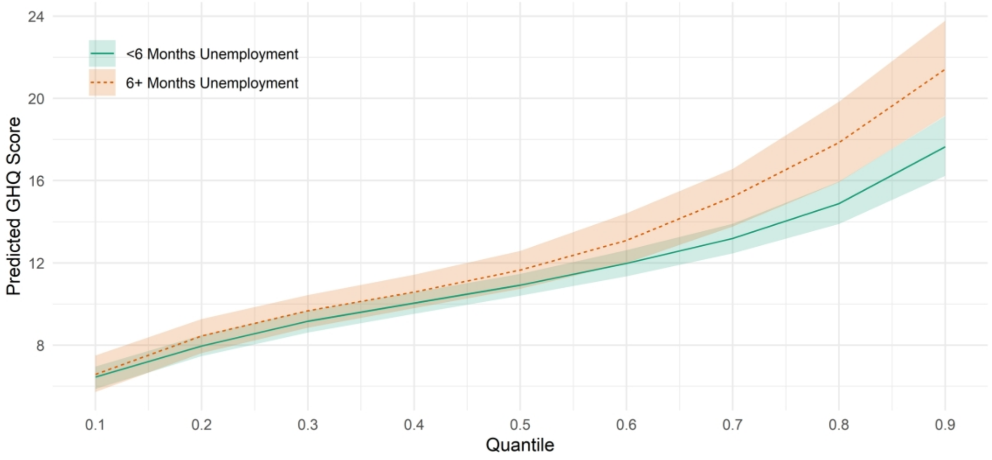

Figure 2: Predicted age 25 GHQ-12 Likert scores by youth unemployment experience, by decile of GHQ-12. Models include adjustment for adolescent mental health, disability and self-rated health, educational attainment, risk behaviours, attitude to school and bullying victimisation, IMD quintile, parental socio-economic class and education, gender, locus of control, and ethnicity. Predicted values derived used sample means for covariates.

209x98mm (300 x 300 DPI)

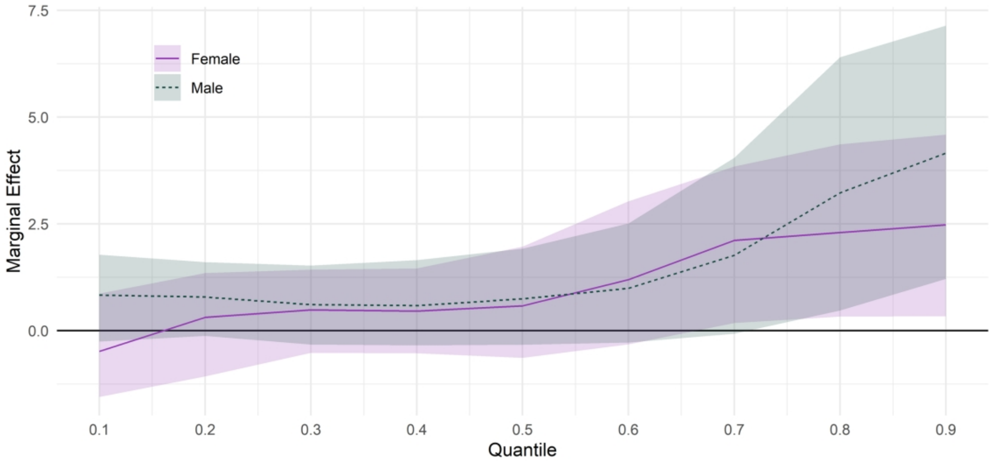

Figure 3: Association between 6+ months youth unemployment between ages 18-20 and GHQ-12 Likert scores at age 25, by gender and decile of GHQ-12. Adjusted for adolescent mental health, disability and self-rated health, educational attainment, risk behaviours, attitude to school and bullying victimisation, IMD quintile, parental socio-economic class and education, gender, locus of control, and ethnicity.

209x98mm (300 x 300 DPI)

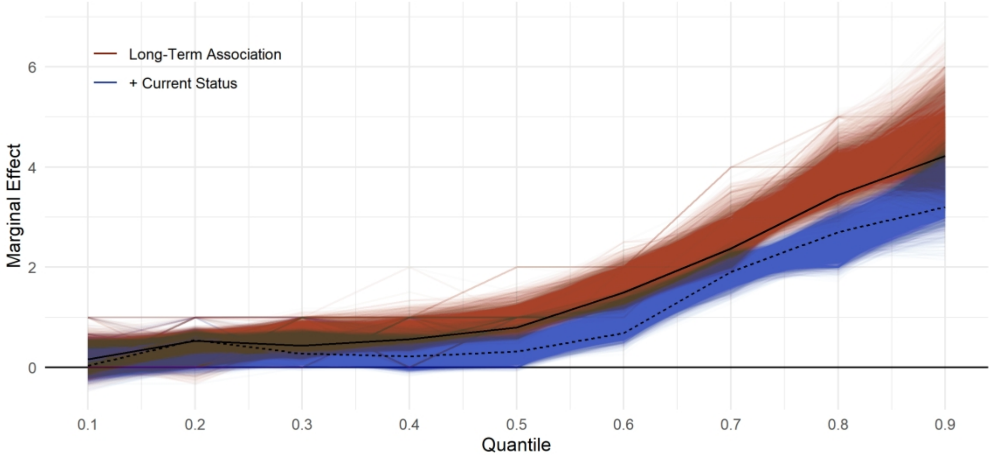

Figure 4: Association between 6+ months youth unemployment between ages 18-20 and GHQ-12 Likert scores at age 25, by decile of GHQ-12. 20,000 models drawn from all possible combinations of covariates. Black lines are results of fully adjusted models, including (dashed) or not including (solid) further adjustment for current economic activity.

209x98mm (300 x 300 DPI)

Supplementary Information

Methods

Locus of Control

Locus of control (LOC) was measured at age 14/15 with participants asked for their level of agreement (strongly agree, agree, disagree, strongly disagree, don't know) with six separate statements. Three of the statements were worded to reflect an internal LOC ("if someone is not a success in life, it is usually their own fault"; "I can pretty much decide what will happen in my life"; "if you work hard at something you'll usually succeed") and three worded to reflect an external LOC ("even if I do well at school, I'll have a hard time getting the right type of job", "people like me don't have much of a chance in life", "how well you get on in this world is mostly a matter of luck"). I place responses onto a five-point scale, centred around "don't know", with external-worded items reverse coded so higher scores indicate less external LOC.

There is no agreed way of combining items to operationalise LOC, both in the Next Steps data specifically and in other large-scale survey datasets which measure LOC (Buddelmeyer & Powdthavee, 2016; Caliendo et al., 2015; Cobb-Clark et al., 2014; Piatek & Pinger, 2016). Researchers using Next Steps data have used different subsets of the LOC items (cf. Crawford et al., 2011; Mendolia & Walker, 2015; Ng-Knight & Schoon, 2017; Wijedasa, 2017),[1] and combined them in several ways, including summing Likert responses (Crawford et al., 2011; Ng-Knight & Schoon, 2017) or extracting latent LOC scores using principal component analysis (Wijedasa, 2017), EFA (Mendolia & Walker, 2015), or CFA (Gladwell et al., 2016).

Though internal and external-worded items were originally conceptualized as tapping opposite ends of a single spectrum (Rotter, 1966), a number of exploratory factor analyses have found internal and external worded items load onto separate factors (Caliendo et al., 2015; Cobb-Clark & Schurer, 2013; Piatek & Pinger, 2016), including an analysis of Next Steps (Mendolia & Walker, 2015). In the Next Steps data, a CFA with two correlated latent factors has superior fit statistics to a single factor CFA model (Table 1). The correlation between the latent factors is just 0.4.

Table 1: Fit Statistics from Confirmatory Factor Analyses of Locus of Control Items

Model	RMSEA (95% CI)	CFI
One Factor	0.073 (0.069, 0.078)	0.818
Two Factor	0.044 (0.039, 0.05)	0.941
Hankins (2008)	0.034 (0.028, 0.04)	0.975

However, an alternative explanation is that responses reflect method effects. A single factor CFA with internal-worded items allowed to covary (i.e akin to Hankins' (2008) GHQ model) has superior fit statistics to the two-factor solution (Table 1). Given the possibility of method effects, the widespread use of only the first factor from EFA (Cobb-Clark, 2015), and the appeal of understanding locus of

[1] Wijedasa (2017) includes another item, "[w]orking hard at school now will help me get on later in life", their LOC measure, while Crawford et al. (2011) include this plus another item, "[d]oing well at school means a lot to me". I do not include these items as they arguably capture opinions about the value of secondary education rather than LOC. Ng-Knight & Schoon (2017) and Gladwell et al. (2016) use the three internal-worded items introduced above, only.

control as a singular construct (Rotter, 1975), we measure LOC by extracting a single factor using the Hankins (2008) CFA model (DWLS estimator).[2]

Note, the items are not drawn from a validated measure of LOC and have poor reliability (Cronbach's α = 0.40). However, the items have been used in published studies before (Chowdry et al., 2011; Crawford et al., 2011; Department for Education et al., 2012; Gladwell et al., 2016; Mendolia & Walker, 2014a, 2014b, 2015; Ng-Knight & Schoon, 2017; Wijedasa, 2017). For instance, Mendolia & Walker (2015) find that participants with external LOC are more likely to be NEET by age 20, while Ng-Knight and Schoon (2017) find evidence that internal LOC moderates the association between low SEP and youth worklessness. The LOC factor we extract is also associated with several variables it should be expected to, including GHQ scores at age 25 and youth unemployment experience.

For peer review only

[2] An alternate model which allows covariance among external-worded items instead has poorer fit statistics.

Figures

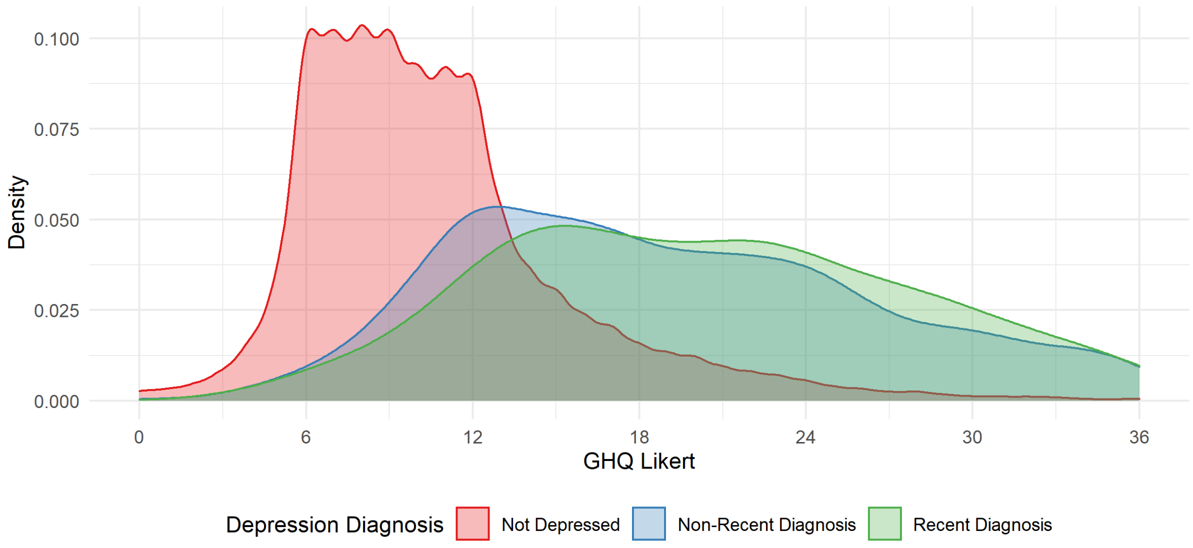

Figure S1: GHQ Likert Scores by recency of depression diagnosis. Recent diagnosis refers to diagnosis in prior two years. Non-recent diagnosis refers to diagnosis more than two years ago. Data drawn from Wave 1 of the United Kingdom Household Longitudinal Study, a representative panel survey of households in the UK.

For peer review only

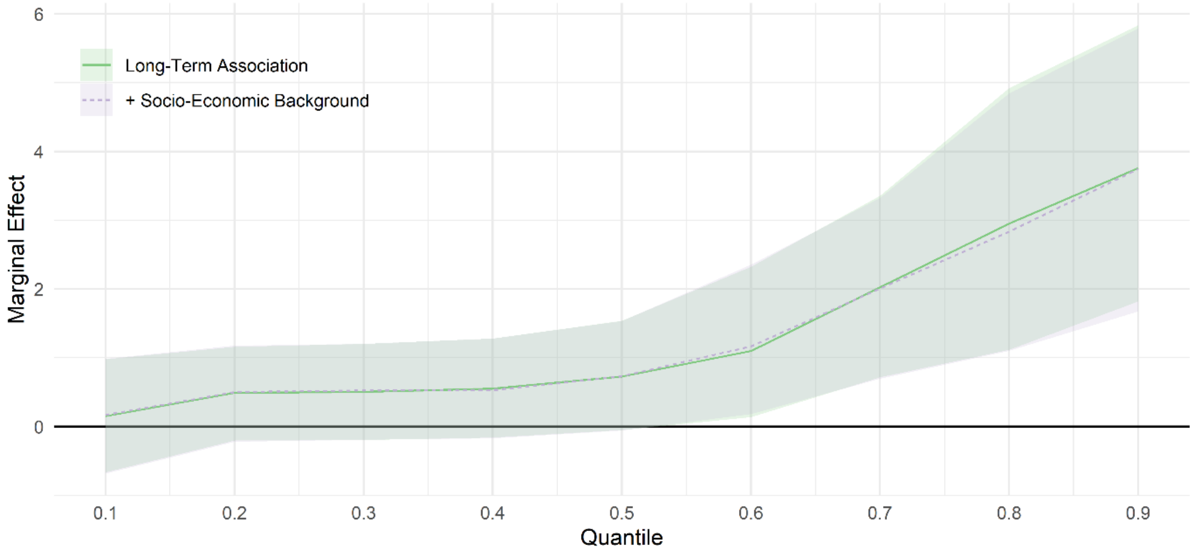

Figure S2: Association between 6+ months youth unemployment between ages 18-20 and GHQ-12 Likert scores at age 25, by decile of GHQ-12. (a) adjusted for adolescent mental health, disability and self-rated health, educational attainment, risk behaviours, attitude to school and bullying victimisation, IMD quintile, parental socio-economic class and education, gender, locus of control, and ethnicity. (b) additionally adjusted for family financial difficulties, number of household children, and household type.

For peer review only

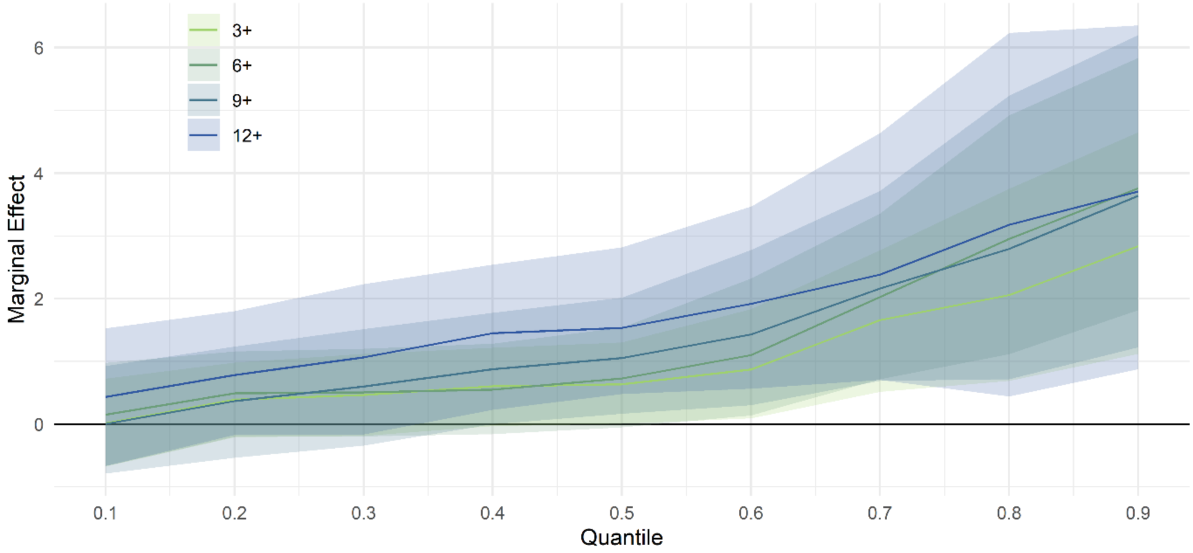

Figure S3: Association between youth unemployment between ages 18-20 and GHQ-12 Likert scores at age 25, by decile of GHQ-12 and cut-off used to define youth unemployment. Models adjusted for adolescent mental health, disability and self-rated health, educational attainment, risk behaviours, attitude to school and bullying victimisation, IMD quintile, parental socio-economic class and education, gender, locus of control, and ethnicity.

For peer review only

Tables

Table S1: Quantile Regression Results. Bivariate association (Column A, Table 2 in main paper)

Variable	Q10	Q20	Q30	Q40	Q50	Q60	Q70	Q80	Q90
6+ Months Unemployment	0<br>(-1, 1)	0<br>(0, 1)	1<br>(0, 2)	1<br>(0, 2)	1<br>(0, 2)	2<br>(0, 2)	3<br>(2, 4)	4<br>(3, 6)	5<br>(3, 8)
Constant	6<br>(5, 6)	7<br>(7, 7)	8<br>(8, 8)	9<br>(9, 10)	11<br>(10, 11)	12<br>(12, 12)	13<br>(13, 13)	16<br>(15, 16)	20<br>(20, 21)
Observations	7,363	7,363	7,363	7,363	7,363	7,363	7,363	7,363	7,363
Imputations	28	28	28	28	28	28	28	28	28

For peer review only

Table S2: Quantile Regression Results. Adjustment for adolescent mental health (Column B, Table 2 in main paper)

Variable	Q10	Q20	Q30	Q40	Q50	Q60	Q70	Q80	Q90
6+ Months Unemployment	0 (-0.79, 0.57)	0.36 (-0.31, 0.93)	0.48 (-0.15, 1.2)	0.67 (-0.07, 1.57)	1.1 (0.35, 1.79)	1.57 (0.67, 2.48)	2.41 (1.27, 3.85)	4 (1.95, 6.2)	6.2 (3.22, 7.62)
GHQ-12 @ Age 14/15	0.12 (0.02, 0.21)	0.14 (0.08, 0.22)	0.16 (0.06, 0.24)	0.18 (0.1, 0.27)	0.25 (0.17, 0.34)	0.33 (0.22, 0.43)	0.42 (0.28, 0.57)	0.56 (0.37, 0.8)	0.67 (0.49, 0.9)
GHQ-12 @ Age 16/17	0.14 (0.1, 0.18)	0.19 (0.15, 0.22)	0.21 (0.18, 0.25)	0.23 (0.2, 0.27)	0.25 (0.2, 0.29)	0.29 (0.24, 0.33)	0.33 (0.26, 0.38)	0.39 (0.32, 0.47)	0.43 (0.33, 0.54)
Constant	4.08 (3.6, 4.47)	5.01 (4.72, 5.36)	5.86 (5.55, 6.2)	6.83 (6.48, 7.2)	7.75 (7.29, 8.2)	8.43 (8, 8.93)	9.3 (8.79, 9.75)	10.5 (9.89, 11.09)	13.56 (12.76, 14.67)
Observations	7,363	7,363	7,363	7,363	7,363	7,363	7,363	7,363	7,363
Imputations	28	28	28	28	28	28	28	28	28

For peer review only

Table S3: Quantile Regression Results. Fully adjusted model excluding current economic activity (Column C, Table 2 in main paper)

	Variable	Q10	Q20	Q30	Q40	Q50	Q60	Q70	Q80	Q90
	6+ Months Unemployment	0.15 (-0.67, 0.98)	0.49 (-0.2, 1.16)	0.51 (-0.19, 1.2)	0.55 (-0.16, 1.28)	0.73 (-0.05, 1.54)	1.1 (0.14, 2.32)	2.03 (0.72, 3.36)	2.95 (1.12, 4.92)	3.76 (1.82, 5.83)
	Female	0.52 (0.17, 0.88)	0.58 (0.26, 0.89)	0.48 (0.16, 0.81)	0.35 (0.01, 0.71)	0.3 (-0.05, 0.65)	0.3 (-0.09, 0.68)	0.21 (-0.27, 0.68)	0 (-0.67, 0.64)	-0.68 (-1.59, 0.22)
	IMD	0 (-0.02, 0.01)	0 (-0.01, 0.01)	0 (-0.01, 0.01)	0 (-0.01, 0.01)	0 (-0.01, 0.02)	0 (-0.01, 0.02)	0.01 (-0.01, 0.02)	0.01 (-0.01, 0.04)	0.03 (-0.01, 0.06)
	Locus of Control	-0.2 (-0.39, -0.01)	-0.11 (-0.32, 0.09)	-0.13 (-0.34, 0.08)	-0.16 (-0.37, 0.06)	-0.12 (-0.35, 0.11)	-0.13 (-0.37, 0.12)	-0.02 (-0.33, 0.28)	0.03 (-0.38, 0.43)	0.02 (-0.54, 0.56)
	GHQ-12 @ Age 14/15	0.08 (-0.02, 0.17)	0.12 (0.04, 0.19)	0.09 (0.01, 0.18)	0.1 (0.01, 0.19)	0.17 (0.07, 0.27)	0.24 (0.14, 0.35)	0.29 (0.16, 0.44)	0.37 (0.18, 0.59)	0.48 (0.24, 0.72)
	GHQ-12 @ Age 16/17	0.13 (0.09, 0.17)	0.17 (0.13, 0.2)	0.2 (0.16, 0.23)	0.21 (0.17, 0.24)	0.23 (0.19, 0.27)	0.27 (0.22, 0.32)	0.31 (0.25, 0.37)	0.35 (0.27, 0.43)	0.4 (0.31, 0.49)
Self-Rated Health @ Age 14/15	Fairly Good	0.13 (-0.27, 0.55)	0.23 (-0.09, 0.55)	0.34 (-0.02, 0.69)	0.39 (0.03, 0.75)	0.37 (-0.02, 0.74)	0.31 (-0.1, 0.71)	0.3 (-0.21, 0.79)	0.51 (-0.15, 1.17)	0.97 (0, 1.98)
	Not Very Good	0.31 (-1.01, 1.58)	0.38 (-0.83, 1.56)	0.42 (-0.82, 2.06)	0.99 (-0.81, 2.23)	0.85 (-0.48, 2.06)	0.67 (-0.73, 2.65)	1.13 (-0.84, 3.71)	1.64 (-0.95, 4.78)	1.7 (-1.14, 5.03)
	Not Good at All	0.12 (-2.52, 1.88)	-0.34 (-1.67, 1.81)	-0.63 (-2.07, 2.18)	-0.31 (-2.67, 2.97)	-0.06 (-3.17, 3.68)	0.08 (-3.01, 4.34)	0.26 (-3.4, 5.97)	-0.01 (-3.86, 7.18)	1.42 (-4.87, 5.87)
Self-Rated Health @ Age 16/17	Fairly Good	0.26 (-0.14, 0.68)	0.18 (-0.16, 0.52)	0.15 (-0.21, 0.52)	0.22 (-0.15, 0.61)	0.17 (-0.23, 0.57)	0.1 (-0.31, 0.54)	0.19 (-0.35, 0.72)	0.24 (-0.47, 0.97)	0.14 (-0.87, 1.22)

	Variable	Q10	Q20	Q30	Q40	Q50	Q60	Q70	Q80	Q90
	Not Very Good	-0.03 (-0.9, 0.75)	-0.09 (-1.1, 0.87)	0.31 (-0.65, 1.32)	0.61 (-0.28, 1.46)	0.59 (-0.25, 1.44)	0.52 (-0.43, 1.85)	0.7 (-0.56, 2.23)	0.61 (-1.01, 2.34)	0.12 (-1.9, 2.15)
	Not Good at All	-0.88 (-2.98, 1.87)	0.15 (-2.45, 1.91)	0.1 (-1.93, 1.93)	0.23 (-1.83, 2.22)	0.69 (-1.93, 2.64)	0.39 (-1.82, 4.8)	1.47 (-1.8, 5.56)	1.59 (-2.31, 5.5)	-0.16 (-3.49, 4.02)
Disabled	Yes, school not affected	0.12 (-0.57, 0.69)	0.08 (-0.42, 0.56)	-0.11 (-0.61, 0.41)	-0.14 (-0.75, 0.55)	0.06 (-0.59, 0.7)	0.24 (-0.51, 1.16)	0.65 (-0.28, 1.51)	0.79 (-0.38, 2.25)	1.49 (-0.39, 3.44)
	Yes, school affected	0.39 (-0.67, 1.32)	0.4 (-0.42, 1.15)	0.33 (-0.4, 1.04)	0.31 (-0.51, 1.23)	0.57 (-0.26, 1.34)	0.6 (-0.29, 1.55)	0.76 (-0.48, 2.51)	1.4 (-0.38, 3.66)	2.24 (-0.18, 4.91)
	Risk Behaviours	-0.03 (-0.18, 0.08)	-0.1 (-0.2, 0.03)	-0.11 (-0.25, 0.06)	-0.07 (-0.23, 0.08)	-0.04 (-0.18, 0.13)	-0.01 (-0.19, 0.18)	0.01 (-0.2, 0.23)	0.01 (-0.29, 0.38)	0.03 (-0.37, 0.41)
	Attitude to School	-0.02 (-0.05, 0.01)	-0.01 (-0.04, 0.02)	-0.02 (-0.05, 0.01)	-0.02 (-0.05, 0.01)	-0.02 (-0.05, 0.01)	-0.02 (-0.05, 0.01)	-0.03 (-0.07, 0.01)	-0.05 (-0.11, 0.01)	-0.06 (-0.13, 0.02)
	# Waves Bullied, 1-3	0.07 (-0.09, 0.24)	0.11 (-0.02, 0.25)	0.17 (0.02, 0.33)	0.23 (0.08, 0.39)	0.27 (0.11, 0.43)	0.33 (0.15, 0.5)	0.39 (0.16, 0.62)	0.55 (0.21, 0.9)	0.7 (0.26, 1.17)
Qualifications	NVQ 4	0.52 (-0.02, 1.08)	0.38 (-0.04, 0.82)	0.48 (0, 0.94)	0.31 (-0.18, 0.82)	0.28 (-0.22, 0.77)	0.35 (-0.19, 0.88)	0.41 (-0.23, 1.06)	0.36 (-0.62, 1.25)	0.27 (-1.23, 1.68)
	NVQ 3	0.55 (-0.05, 1.14)	0.38 (-0.03, 0.82)	0.38 (-0.1, 0.86)	0.32 (-0.23, 0.87)	0.38 (-0.16, 0.9)	0.42 (-0.16, 0.99)	0.46 (-0.21, 1.1)	0.32 (-0.7, 1.32)	0.21 (-1.29, 1.66)
	NVQ 2	0.26 (-0.33, 0.86)	0.26 (-0.19, 0.72)	0.26 (-0.24, 0.77)	0.09 (-0.45, 0.64)	-0.02 (-0.55, 0.5)	-0.06 (-0.67, 0.54)	-0.09 (-0.82, 0.65)	-0.56 (-1.65, 0.49)	-1.33 (-2.89, 0.12)

	Variable	Q10	Q20	Q30	Q40	Q50	Q60	Q70	Q80	Q90
	NVQ 1	-0.48 (-1.42, 0.35)	-0.41 (-1.12, 0.3)	-0.28 (-1.04, 0.46)	-0.32 (-1.1, 0.49)	-0.13 (-0.9, 0.68)	0.13 (-0.73, 0.98)	0.23 (-0.77, 1.46)	0.73 (-0.89, 2.35)	0.69 (-1.45, 3.03)
	No/Other Qual	-0.39 (-1.37, 0.58)	-0.19 (-1.04, 0.59)	-0.11 (-0.93, 0.73)	-0.16 (-1.05, 0.76)	-0.04 (-0.97, 0.91)	0.29 (-0.75, 1.37)	0.74 (-0.56, 2.36)	1.17 (-0.78, 3.17)	0.15 (-2.06, 2.48)
Parental NS-SEC	Intermediate	0.14 (-0.39, 0.64)	0.09 (-0.32, 0.49)	0 (-0.4, 0.41)	-0.08 (-0.52, 0.38)	-0.15 (-0.63, 0.39)	-0.15 (-0.69, 0.4)	-0.08 (-0.7, 0.59)	0.23 (-0.64, 1.16)	0.43 (-0.83, 1.77)
	Routine	0.3 (-0.15, 0.75)	0.24 (-0.15, 0.64)	0.34 (-0.1, 0.75)	0.38 (-0.09, 0.84)	0.3 (-0.2, 0.79)	0.22 (-0.31, 0.78)	0.35 (-0.27, 0.96)	0.69 (-0.12, 1.49)	0.77 (-0.52, 2)
	LTU	-0.19 (-1.51, 0.97)	-0.25 (-1.31, 0.74)	-0.2 (-1.22, 0.9)	-0.05 (-1.2, 1.2)	0 (-1.24, 1.28)	0.32 (-1.15, 1.95)	1.03 (-0.64, 2.78)	1.44 (-0.62, 3.75)	1.98 (-0.85, 4.88)
Parental Education	Other HE	-0.24 (-0.8, 0.32)	-0.11 (-0.57, 0.35)	-0.21 (-0.75, 0.36)	-0.01 (-0.58, 0.54)	0.05 (-0.55, 0.63)	0.09 (-0.54, 0.69)	0.13 (-0.64, 0.89)	0.07 (-0.87, 1.07)	0.33 (-0.97, 1.74)
	A-Level	-0.24 (-0.84, 0.33)	-0.19 (-0.68, 0.31)	-0.37 (-0.88, 0.2)	-0.27 (-0.83, 0.3)	-0.19 (-0.83, 0.45)	-0.11 (-0.78, 0.52)	-0.29 (-1.04, 0.43)	-0.61 (-1.57, 0.42)	-0.36 (-1.83, 1.21)
	GCSE A-C	-0.08 (-0.65, 0.49)	0 (-0.48, 0.49)	-0.11 (-0.6, 0.4)	-0.15 (-0.66, 0.35)	-0.16 (-0.79, 0.47)	-0.04 (-0.71, 0.59)	-0.1 (-0.82, 0.6)	-0.44 (-1.38, 0.54)	-0.5 (-1.95, 0.93)
	Other/None	-0.42 (-1.08, 0.28)	-0.03 (-0.66, 0.57)	-0.06 (-0.72, 0.57)	0.08 (-0.59, 0.81)	0.37 (-0.41, 1.16)	0.51 (-0.35, 1.34)	0.48 (-0.53, 1.5)	0.46 (-0.79, 1.75)	1.43 (-0.58, 3.39)
Ethnicity	Mixed	-0.69 (-1.77, 0.17)	-0.67 (-1.62, 0.16)	-0.6 (-1.56, 0.19)	-0.72 (-1.54, 0.02)	-0.98 (-1.75, -0.01)	-0.86 (-1.94, 0.5)	-0.23 (-1.75, 1.75)	0.47 (-1.27, 3.12)	1.42 (-1.57, 5.03)

Variable	Q10	Q20	Q30	Q40	Q50	Q60	Q70	Q80	Q90
Indian	-1.17 (-1.92, -0.27)	-0.81 (-1.43, -0.22)	-0.86 (-1.52, -0.07)	-0.62 (-1.44, 0.16)	-0.62 (-1.36, 0.13)	-0.48 (-1.35, 0.43)	-0.31 (-1.29, 0.96)	0.13 (-1.37, 1.66)	0.12 (-1.98, 3.03)
Pakistani	-0.65 (-1.49, 0.22)	-0.44 (-1.19, 0.22)	-0.61 (-1.27, 0.07)	-0.89 (-1.65, -0.14)	-1.21 (-1.99, -0.43)	-1.2 (-1.99, -0.29)	-1 (-2.13, 0.16)	-1.24 (-2.62, 0.16)	-1.9 (-3.98, 0.47)
Bangladeshi	-1.72 (-3.04, -0.33)	-1.18 (-2.63, 0.03)	-0.75 (-1.87, 0.13)	-0.86 (-1.84, 0.12)	-1.08 (-2.16, 0.15)	-1.05 (-2.26, 0.07)	-1.42 (-2.66, 0)	-1.95 (-3.68, -0.23)	-3.41 (-5.54, -0.95)
Black African	-0.45 (-1.54, 0.41)	-0.54 (-1.43, 0.46)	-0.44 (-1.54, 0.6)	-0.42 (-1.52, 0.69)	-0.6 (-1.66, 0.47)	-0.73 (-2, 0.73)	-0.69 (-2.04, 0.87)	-1.02 (-2.72, 0.86)	-1.8 (-3.95, 1.38)
Black Caribbean	-1.8 (-2.93, -0.58)	-1.22 (-2.08, -0.33)	-1.06 (-1.89, -0.22)	-1.03 (-1.95, 0.07)	-0.75 (-1.88, 0.17)	-0.76 (-1.68, 0.34)	-0.66 (-1.87, 0.82)	-0.72 (-2.34, 1.28)	-1.28 (-3.57, 1.99)
Other	-0.82 (-3.1, 0.55)	-0.32 (-1.54, 0.75)	-0.18 (-1.23, 1.16)	0.27 (-1.19, 1.63)	0.47 (-0.9, 2.13)	1.02 (-0.72, 2.46)	1.11 (-0.38, 2.64)	0.86 (-0.82, 3.36)	-0.07 (-2.45, 2.77)
Constant	4.37 (2.88, 5.65)	5.07 (3.85, 6.31)	6.28 (5.02, 7.4)	7.06 (5.87, 8.29)	7.72 (6.36, 9.07)	8.02 (6.61, 9.42)	9.03 (7.29, 10.89)	10.91 (8.12, 13.58)	13.59 (10.19, 16.82)
Observations	7,363	7,363	7,363	7,363	7,363	7,363	7,363	7,363	7,363
Imputations	28	28	28	28	28	28	28	28	28

Table S4: Quantile Regression Results. Fully adjusted model including current economic activity (Column D, Table 2 in main paper)

	Variable	Q10	Q20	Q30	Q40	Q50	Q60	Q70	Q80	Q90
	6+ Months Unemployment	0.1 (-0.78, 0.95)	0.42 (-0.3, 1.03)	0.28 (-0.4, 0.94)	0.22 (-0.46, 0.92)	0.22 (-0.59, 1.09)	0.52 (-0.4, 1.73)	1.55 (0.07, 2.82)	2.24 (0.73, 4.02)	2.69 (0.92, 4.42)
	Female	0.45 (0.1, 0.81)	0.54 (0.23, 0.84)	0.46 (0.14, 0.78)	0.31 (-0.03, 0.65)	0.21 (-0.16, 0.57)	0.2 (-0.19, 0.58)	0.11 (-0.37, 0.56)	-0.18 (-0.79, 0.41)	-0.76 (-1.64, 0.11)
	IMD	0 (-0.02, 0.01)	0 (-0.01, 0.01)	0 (-0.01, 0.01)	0 (-0.02, 0.01)	0 (-0.01, 0.01)	0 (-0.01, 0.02)	0 (-0.02, 0.02)	0 (-0.02, 0.02)	0.02 (-0.01, 0.05)
	Locus of Control	-0.19 (-0.38, 0.01)	-0.09 (-0.29, 0.1)	-0.06 (-0.27, 0.16)	-0.08 (-0.29, 0.15)	-0.05 (-0.28, 0.18)	-0.02 (-0.26, 0.2)	0.05 (-0.25, 0.35)	0.14 (-0.27, 0.51)	-0.02 (-0.54, 0.5)
Current Economic Activity	Education	0.67 (-0.26, 1.47)	0.59 (-0.09, 1.26)	0.58 (-0.2, 1.39)	0.68 (-0.14, 1.58)	0.84 (-0.11, 1.78)	1.12 (0.16, 2.2)	1.6 (0.29, 3.03)	2.23 (0.41, 4.37)	2.39 (0.55, 4.47)
	Inactive	0.53 (-0.08, 1.28)	0.4 (-0.26, 1.02)	0.48 (-0.26, 1.23)	0.83 (-0.03, 1.7)	1.21 (0.28, 2.41)	1.95 (0.74, 3.2)	2.78 (1.33, 4.35)	3.74 (1.94, 5.96)	4.64 (2.58, 6.78)
	Unemployed	1.45 (0.34, 2.59)	2.16 (0.98, 3.03)	2.36 (1.53, 3.17)	2.52 (1.65, 3.54)	2.88 (1.67, 4.03)	3.28 (2.13, 4.48)	4.01 (2.51, 5.82)	4.71 (3.02, 6.28)	4.67 (2.84, 6.64)
	GHQ-12 @ Age 14/15	0.09 (-0.01, 0.18)	0.12 (0.04, 0.2)	0.1 (0.02, 0.18)	0.11 (0.02, 0.2)	0.17 (0.07, 0.27)	0.24 (0.13, 0.34)	0.28 (0.16, 0.43)	0.37 (0.2, 0.57)	0.51 (0.29, 0.75)
	GHQ-12 @ Age 16/17	0.13 (0.09, 0.17)	0.16 (0.12, 0.2)	0.19 (0.15, 0.23)	0.21 (0.17, 0.24)	0.23 (0.19, 0.27)	0.26 (0.22, 0.31)	0.29 (0.24, 0.35)	0.32 (0.25, 0.4)	0.37 (0.29, 0.46)
Self-Rated Health @ Age 14/15	Fairly Good	0.11 (-0.29, 0.52)	0.21 (-0.12, 0.53)	0.3 (-0.05, 0.65)	0.42 (0.07, 0.76)	0.46 (0.06, 0.85)	0.37 (-0.04, 0.77)	0.31 (-0.18, 0.79)	0.48 (-0.15, 1.12)	0.99 (0.05, 1.97)

	Variable	Q10	Q20	Q30	Q40	Q50	Q60	Q70	Q80	Q90
	Not Very Good	0.18 (-1.22, 1.5)	0.5 (-0.94, 1.68)	0.46 (-0.73, 1.95)	0.87 (-0.79, 2.16)	0.86 (-0.57, 2.02)	0.47 (-0.77, 2.23)	0.8 (-1.06, 3.7)	1.61 (-1.26, 5.06)	2.45 (-0.4, 5.22)
	Not Good at All	-0.27 (-2.68, 1.73)	-0.46 (-2.57, 1.71)	-0.73 (-2.33, 2.02)	-0.21 (-2.84, 2.66)	-0.24 (-3.1, 3)	-0.56 (-3.13, 3.44)	-0.98 (-3.85, 5.02)	-0.38 (-4.85, 5.76)	-0.11 (-5.23, 5.46)
Self-Rated Health @ Age 16/17	Fairly Good	0.24 (-0.18, 0.66)	0.21 (-0.14, 0.55)	0.13 (-0.23, 0.5)	0.2 (-0.18, 0.6)	0.2 (-0.21, 0.61)	0.08 (-0.33, 0.51)	0.01 (-0.5, 0.53)	0.24 (-0.44, 0.93)	0.19 (-0.8, 1.2)
	Not Very Good	-0.07 (-0.94, 0.82)	-0.03 (-1.06, 0.92)	0.33 (-0.66, 1.31)	0.55 (-0.34, 1.39)	0.52 (-0.37, 1.39)	0.41 (-0.55, 1.6)	0.57 (-0.71, 2.09)	0.69 (-1.01, 2.26)	-0.27 (-1.96, 1.53)
	Not Good at All	-0.93 (-2.87, 1.78)	-0.13 (-2.54, 1.99)	0.24 (-2.38, 2.07)	0.38 (-1.69, 2.36)	0.84 (-1.66, 2.7)	0.61 (-1.66, 4.31)	1.09 (-1.6, 5.38)	1.31 (-2.16, 4.9)	-1.04 (-4.08, 2.68)
Disabled	Yes, school not affected	0.15 (-0.51, 0.72)	0.06 (-0.45, 0.55)	-0.04 (-0.54, 0.48)	-0.11 (-0.69, 0.54)	0.1 (-0.61, 0.79)	0.21 (-0.47, 1.06)	0.63 (-0.34, 1.57)	0.63 (-0.46, 1.91)	1.23 (-0.5, 3.11)
	Yes, school affected	0.25 (-0.88, 1.19)	0.38 (-0.47, 1.13)	0.35 (-0.37, 1.02)	0.16 (-0.56, 0.97)	0.29 (-0.62, 1.2)	0.39 (-0.53, 1.34)	0.48 (-0.61, 1.95)	0.83 (-0.81, 2.66)	1.39 (-1.1, 4.42)
	Risk Behaviours	-0.06 (-0.21, 0.07)	-0.1 (-0.21, 0.03)	-0.1 (-0.24, 0.07)	-0.07 (-0.24, 0.08)	-0.06 (-0.22, 0.11)	-0.04 (-0.22, 0.15)	-0.02 (-0.22, 0.19)	-0.03 (-0.3, 0.29)	-0.09 (-0.43, 0.27)
	Attitude to School	-0.02 (-0.05, 0.01)	-0.01 (-0.04, 0.02)	-0.03 (-0.05, 0.01)	-0.02 (-0.05, 0.01)	-0.02 (-0.05, 0.01)	-0.03 (-0.06, 0.01)	-0.03 (-0.07, 0)	-0.05 (-0.11, 0.01)	-0.05 (-0.12, 0.03)
	# Waves Bullied, 1-3	0.08 (-0.09, 0.25)	0.1 (-0.04, 0.23)	0.16 (0.01, 0.31)	0.21 (0.06, 0.36)	0.23 (0.06, 0.4)	0.3 (0.12, 0.47)	0.34 (0.13, 0.56)	0.5 (0.17, 0.82)	0.63 (0.24, 1.04)

	Variable	Q10	Q20	Q30	Q40	Q50	Q60	Q70	Q80	Q90
Qualifications	NVQ 4	0.49 (-0.04, 1.03)	0.34 (-0.07, 0.79)	0.39 (-0.08, 0.86)	0.31 (-0.18, 0.81)	0.3 (-0.21, 0.8)	0.37 (-0.16, 0.9)	0.47 (-0.18, 1.11)	0.43 (-0.51, 1.31)	0.25 (-1.1, 1.57)
	NVQ 3	0.53 (-0.07, 1.12)	0.41 (-0.02, 0.86)	0.36 (-0.12, 0.85)	0.29 (-0.25, 0.84)	0.38 (-0.19, 0.94)	0.48 (-0.09, 1.05)	0.53 (-0.15, 1.19)	0.37 (-0.63, 1.37)	0.26 (-1.12, 1.56)
	NVQ 2	0.26 (-0.34, 0.89)	0.29 (-0.16, 0.75)	0.26 (-0.24, 0.76)	0.12 (-0.41, 0.67)	0.02 (-0.51, 0.56)	-0.08 (-0.67, 0.53)	-0.12 (-0.88, 0.58)	-0.62 (-1.63, 0.36)	-1.15 (-2.59, 0.19)
	NVQ 1	-0.58 (-1.54, 0.29)	-0.57 (-1.26, 0.19)	-0.35 (-1.05, 0.37)	-0.4 (-1.18, 0.39)	-0.26 (-1.05, 0.56)	0.02 (-0.85, 0.89)	0.08 (-0.95, 1.14)	0.28 (-1.31, 1.95)	0.19 (-1.81, 2.27)
	No/Other Qual	-0.48 (-1.46, 0.5)	-0.24 (-1.11, 0.58)	-0.24 (-1.05, 0.63)	-0.23 (-1.15, 0.67)	-0.14 (-1.09, 0.81)	0.03 (-0.98, 1.05)	0.29 (-1.02, 1.65)	0.08 (-1.64, 1.85)	-1.06 (-3.09, 1.16)
Parental NS-SEC	Intermediate	0.13 (-0.38, 0.66)	0.1 (-0.31, 0.48)	-0.03 (-0.43, 0.36)	-0.11 (-0.55, 0.35)	-0.17 (-0.69, 0.36)	-0.11 (-0.64, 0.44)	0.05 (-0.54, 0.67)	0.32 (-0.54, 1.23)	0.54 (-0.64, 1.83)
	Routine	0.27 (-0.2, 0.74)	0.25 (-0.15, 0.67)	0.29 (-0.13, 0.7)	0.32 (-0.13, 0.78)	0.26 (-0.24, 0.79)	0.17 (-0.35, 0.74)	0.38 (-0.23, 0.98)	0.66 (-0.15, 1.4)	0.41 (-0.77, 1.68)
	LTU	-0.12 (-1.46, 1.04)	-0.2 (-1.23, 0.76)	-0.14 (-1.13, 0.95)	0.08 (-1.09, 1.36)	0.17 (-1.11, 1.36)	0.09 (-1.13, 1.53)	0.72 (-0.96, 2.58)	1.3 (-0.75, 3.62)	1.76 (-0.83, 4.31)
Parental Education	Other HE	-0.2 (-0.76, 0.35)	-0.1 (-0.57, 0.37)	-0.17 (-0.71, 0.41)	0.02 (-0.54, 0.56)	0.08 (-0.5, 0.65)	0.14 (-0.5, 0.74)	0.18 (-0.56, 0.9)	0.21 (-0.73, 1.23)	0.76 (-0.52, 2.1)
	A-Level	-0.22 (-0.83, 0.35)	-0.17 (-0.66, 0.32)	-0.33 (-0.85, 0.22)	-0.26 (-0.81, 0.29)	-0.13 (-0.79, 0.5)	-0.09 (-0.75, 0.53)	-0.22 (-0.96, 0.49)	-0.36 (-1.31, 0.64)	0.09 (-1.32, 1.58)

	Variable	Q10	Q20	Q30	Q40	Q50	Q60	Q70	Q80	Q90
	GCSE A-C	-0.05 (-0.64, 0.55)	0.05 (-0.45, 0.54)	-0.07 (-0.57, 0.43)	-0.13 (-0.62, 0.37)	-0.11 (-0.74, 0.51)	0.01 (-0.64, 0.65)	0.02 (-0.68, 0.7)	-0.25 (-1.12, 0.69)	-0.01 (-1.42, 1.29)
	Other/None	-0.51 (-1.18, 0.2)	-0.12 (-0.72, 0.49)	-0.08 (-0.73, 0.54)	-0.01 (-0.65, 0.69)	0.28 (-0.54, 1.12)	0.45 (-0.4, 1.26)	0.29 (-0.66, 1.27)	0.48 (-0.79, 1.84)	1.67 (-0.16, 3.46)
Ethnicity	Mixed	-0.77 (-1.92, 0.18)	-0.64 (-1.57, 0.13)	-0.63 (-1.54, 0.17)	-0.73 (-1.59, -0.03)	-1 (-1.73, -0.09)	-0.93 (-1.99, 0.45)	-0.37 (-1.78, 1.59)	0.43 (-1.32, 3.03)	1.71 (-1.44, 5.01)
	Indian	-1.17 (-1.88, -0.38)	-0.85 (-1.6, -0.22)	-0.78 (-1.47, -0.05)	-0.64 (-1.42, 0.17)	-0.55 (-1.35, 0.22)	-0.42 (-1.22, 0.44)	-0.18 (-1.21, 1.03)	0.18 (-1.16, 1.37)	-0.77 (-2.28, 1.4)
	Pakistani	-0.59 (-1.5, 0.25)	-0.59 (-1.35, 0.07)	-0.71 (-1.43, -0.04)	-0.98 (-1.71, -0.2)	-1.26 (-2.06, -0.44)	-1.27 (-2.1, -0.35)	-1.03 (-2.19, 0.14)	-1.07 (-2.45, 0.29)	-1.98 (-3.85, 0.47)
	Bangladeshi	-1.71 (-3.03, -0.3)	-1.23 (-2.68, -0.07)	-0.79 (-1.95, 0.13)	-0.84 (-1.85, 0.14)	-1.07 (-2.15, 0.2)	-0.92 (-2.14, 0.25)	-1.02 (-2.31, 0.37)	-1.44 (-3.16, 0.2)	-2.82 (-4.84, -0.58)
	Black African	-0.47 (-1.51, 0.44)	-0.52 (-1.38, 0.35)	-0.52 (-1.48, 0.46)	-0.62 (-1.65, 0.34)	-0.94 (-1.98, 0.34)	-0.76 (-2.16, 0.79)	-0.56 (-1.99, 0.99)	-0.64 (-2.29, 1.28)	-1.28 (-3.4, 1.69)
	Black Caribbean	-1.77 (-2.87, -0.63)	-1.28 (-2.17, -0.42)	-1.16 (-1.99, -0.36)	-1.22 (-2.08, -0.14)	-0.99 (-2.2, 0.06)	-0.82 (-1.83, 0.2)	-0.75 (-1.89, 0.67)	-0.85 (-2.3, 1.2)	-1.46 (-3.66, 1.08)
	Other	-0.92 (-3.08, 0.41)	-0.45 (-1.8, 0.7)	-0.21 (-1.34, 0.89)	-0.07 (-1.33, 1.43)	0.34 (-1.22, 1.88)	0.71 (-0.94, 2.18)	0.91 (-0.73, 2.57)	0.72 (-1.11, 3.13)	0.4 (-2.41, 3.51)
	Constant	4.34 (2.8, 5.66)	4.94 (3.73, 6.2)	6.36 (5.07, 7.49)	7.06 (5.89, 8.29)	7.68 (6.3, 9.05)	8.32 (6.83, 9.78)	9.27 (7.55, 11.14)	11.15 (8.6, 13.56)	13.06 (9.91, 16.24)

Variable	Q10	Q20	Q30	Q40	Q50	Q60	Q70	Q80	Q90
Observations	7,363	7,363	7,363	7,363	7,363	7,363	7,363	7,363	7,363
Imputations	28	28	28	28	28	28	28	28	28

For peer review only

Table S5: Quantile Regression Results. Fully adjusted model, males only (Column E, Table 2 in main paper)

	Variable	Q10	Q20	Q30	Q40	Q50	Q60	Q70	Q80	Q90
	6+ Months Unemployment	0.83 (-0.26, 1.78)	0.79 (-0.13, 1.6)	0.61 (-0.32, 1.52)	0.59 (-0.35, 1.65)	0.75 (-0.33, 1.92)	0.99 (-0.28, 2.51)	1.76 (-0.07, 4.05)	3.22 (0.47, 6.4)	4.16 (1.21, 7.14)
	IMD	-0.01 (-0.03, 0.01)	-0.01 (-0.02, 0.01)	-0.01 (-0.03, 0.01)	-0.01 (-0.03, 0.02)	0 (-0.02, 0.02)	0 (-0.02, 0.02)	0 (-0.03, 0.03)	0 (-0.04, 0.04)	0.01 (-0.04, 0.06)
	Locus of Control	-0.07 (-0.37, 0.25)	-0.06 (-0.35, 0.23)	-0.15 (-0.5, 0.18)	-0.26 (-0.59, 0.11)	-0.22 (-0.54, 0.12)	-0.15 (-0.48, 0.2)	0 (-0.45, 0.44)	0.16 (-0.47, 0.8)	0.32 (-0.46, 1.06)
	GHQ-12 @ Age 14/15	0.06 (-0.13, 0.23)	0.07 (-0.08, 0.2)	0.04 (-0.11, 0.21)	0.08 (-0.09, 0.26)	0.19 (-0.01, 0.38)	0.29 (0.09, 0.47)	0.38 (0.15, 0.67)	0.56 (0.2, 0.93)	0.64 (0.2, 1.09)
	GHQ-12 @ Age 16/17	0.18 (0.12, 0.24)	0.21 (0.15, 0.26)	0.24 (0.18, 0.3)	0.25 (0.19, 0.31)	0.26 (0.19, 0.33)	0.28 (0.21, 0.35)	0.3 (0.21, 0.4)	0.33 (0.2, 0.47)	0.41 (0.24, 0.57)
Self-Rated Health @ Age 14/15	Fairly Good	0.07 (-0.51, 0.68)	0.2 (-0.28, 0.68)	0.25 (-0.29, 0.78)	0.29 (-0.27, 0.82)	0.32 (-0.27, 0.88)	0.21 (-0.44, 0.86)	0.16 (-0.65, 1.02)	0.64 (-0.5, 1.77)	1.38 (-0.18, 2.92)
	Not Very Good	-0.1 (-2.14, 2.2)	0.06 (-2.21, 2.22)	-0.03 (-2.33, 2.48)	-0.03 (-2.37, 2.76)	0.29 (-2.75, 2.87)	0.19 (-2.84, 3.25)	0.02 (-3.23, 5.55)	0.12 (-3.89, 7.7)	2.5 (-4.47, 7.83)
	Not Good at All	1.09 (-4.24, 4.36)	0.76 (-3.2, 4.69)	0.46 (-3.18, 5.69)	0.18 (-3.73, 5.22)	0.08 (-4.13, 5.69)	-0.12 (-4.35, 10.17)	-0.77 (-5.26, 10.26)	-0.85 (-6.44, 9.45)	-1.05 (-6.9, 9.15)
Self-Rated Health @ Age 16/17	Fairly Good	0.29 (-0.37, 0.92)	0.17 (-0.37, 0.71)	0.14 (-0.4, 0.69)	0.19 (-0.41, 0.79)	0.14 (-0.48, 0.77)	0.19 (-0.45, 0.84)	0.28 (-0.59, 1.15)	0.1 (-1.03, 1.21)	0.15 (-1.39, 1.86)
	Not Very Good	0.48 (-1, 1.86)	0.12 (-1.51, 2)	0.46 (-1.5, 2.36)	0.85 (-1.12, 2.26)	0.76 (-0.68, 2.49)	0.85 (-0.83, 3.38)	1.21 (-1.3, 4.2)	1.3 (-1.95, 4.79)	0.53 (-2.83, 4.13)

	Variable	Q10	Q20	Q30	Q40	Q50	Q60	Q70	Q80	Q90
	Not Good at All	0.69 (-4.73, 3.6)	0.83 (-3.31, 3.32)	0.36 (-2.69, 2.98)	0.49 (-2.63, 2.69)	0.58 (-2.96, 6.11)	0.28 (-3.19, 8.45)	0.86 (-3.07, 9.22)	2.01 (-3.52, 8.09)	-1.29 (-5.46, 5.65)
Disabled	Yes, school not affected	0.09 (-0.84, 0.96)	0.08 (-0.66, 0.77)	-0.15 (-0.86, 0.58)	-0.25 (-1.04, 0.64)	-0.11 (-1.03, 0.8)	-0.01 (-0.98, 1.15)	0.18 (-0.97, 1.38)	0.13 (-1.37, 2.26)	1.25 (-1.31, 4.09)
	Yes, school affected	0.68 (-0.86, 1.97)	0.83 (-0.34, 1.93)	0.75 (-0.41, 1.89)	0.69 (-0.49, 1.89)	0.78 (-0.39, 2.02)	0.91 (-0.43, 2.61)	1.35 (-0.6, 3.91)	1.78 (-0.73, 5.54)	3.2 (-0.75, 6.65)
	Risk Behaviours	0 (-0.21, 0.19)	-0.04 (-0.23, 0.16)	-0.03 (-0.23, 0.18)	-0.03 (-0.24, 0.17)	-0.02 (-0.24, 0.24)	0.01 (-0.25, 0.28)	0.01 (-0.32, 0.38)	0.05 (-0.42, 0.63)	0.14 (-0.45, 0.66)
	Attitude to School	0 (-0.05, 0.06)	-0.01 (-0.05, 0.04)	-0.01 (-0.06, 0.04)	-0.02 (-0.06, 0.03)	-0.02 (-0.07, 0.03)	-0.03 (-0.08, 0.02)	-0.05 (-0.12, 0.01)	-0.07 (-0.15, 0.01)	-0.08 (-0.18, 0.03)
	# Waves Bullied, 1-3	0.2 (-0.06, 0.47)	0.15 (-0.06, 0.35)	0.21 (-0.02, 0.45)	0.29 (0.05, 0.52)	0.3 (0.05, 0.54)	0.34 (0.07, 0.61)	0.37 (0.02, 0.73)	0.47 (-0.06, 1.06)	0.71 (-0.01, 1.49)
Qualifications	NVQ 4	0.54 (-0.29, 1.5)	0.45 (-0.23, 1.18)	0.44 (-0.33, 1.17)	0.18 (-0.65, 0.99)	-0.07 (-0.89, 0.79)	-0.06 (-0.91, 0.8)	-0.02 (-1.07, 0.99)	0.1 (-1.52, 1.55)	-0.17 (-2.75, 2.24)
	NVQ 3	0.93 (0.02, 1.87)	0.76 (0.08, 1.45)	0.73 (-0.07, 1.53)	0.62 (-0.27, 1.52)	0.37 (-0.51, 1.28)	0.24 (-0.62, 1.1)	0.09 (-0.96, 1.12)	-0.03 (-1.59, 1.51)	-0.6 (-3.34, 1.87)
	NVQ 2	0.7 (-0.24, 1.7)	0.74 (0.03, 1.44)	0.68 (-0.14, 1.48)	0.39 (-0.51, 1.25)	-0.04 (-0.88, 0.86)	-0.24 (-1.18, 0.69)	-0.31 (-1.45, 0.78)	-0.72 (-2.36, 0.81)	-1.77 (-4.36, 0.62)
	NVQ 1	-0.59 (-1.92, 0.73)	-0.3 (-1.35, 0.72)	-0.18 (-1.26, 0.89)	-0.35 (-1.46, 0.84)	-0.31 (-1.44, 0.89)	-0.2 (-1.43, 1.04)	0 (-1.61, 1.84)	0.52 (-1.78, 3.09)	0.49 (-2.83, 4.04)

	Variable	Q10	Q20	Q30	Q40	Q50	Q60	Q70	Q80	Q90
	No/Other Qual	-0.53 (-1.86, 0.88)	-0.42 (-1.66, 0.96)	-0.13 (-1.53, 1.21)	-0.24 (-1.64, 1.18)	-0.36 (-1.77, 1.1)	-0.24 (-1.8, 1.49)	0.25 (-1.76, 2.67)	0.34 (-2.36, 3.27)	-1.01 (-4.36, 2.66)
Parental NS-SEC	Intermediate	-0.2 (-0.96, 0.56)	-0.36 (-0.96, 0.24)	-0.44 (-1.09, 0.22)	-0.54 (-1.25, 0.17)	-0.51 (-1.27, 0.29)	-0.27 (-1.05, 0.58)	0.12 (-0.89, 1.22)	0.48 (-0.94, 1.97)	0.98 (-1, 3.24)
	Routine	-0.25 (-0.97, 0.49)	-0.22 (-0.82, 0.41)	-0.13 (-0.8, 0.53)	-0.14 (-0.84, 0.54)	-0.17 (-0.86, 0.51)	-0.16 (-0.89, 0.62)	-0.02 (-0.95, 0.95)	0 (-1.3, 1.33)	0.04 (-1.8, 1.96)
	LTU	-0.24 (-1.69, 1.09)	-0.55 (-1.87, 1.14)	-0.39 (-1.95, 1.4)	-0.46 (-2.12, 1.43)	-0.42 (-2.17, 1.44)	-0.29 (-2.18, 1.88)	-0.08 (-2.44, 2.93)	0.54 (-2.67, 4.39)	1.56 (-2.5, 5.76)
Parental Education	Other HE	-0.39 (-1.32, 0.44)	-0.16 (-0.88, 0.54)	-0.18 (-0.98, 0.66)	-0.06 (-0.89, 0.79)	0.15 (-0.75, 0.98)	0.15 (-0.76, 1.08)	0.11 (-1.04, 1.35)	0.18 (-1.37, 1.84)	0.91 (-1.35, 3.62)
	A-Level	0.19 (-0.65, 1)	0.29 (-0.45, 0.98)	0.13 (-0.67, 0.99)	0.26 (-0.61, 1.12)	0.46 (-0.44, 1.34)	0.55 (-0.42, 1.52)	0.46 (-0.69, 1.67)	0.62 (-1.1, 2.31)	1.37 (-0.95, 3.84)
	GCSE A-C	0.23 (-0.67, 1.06)	0.45 (-0.28, 1.16)	0.37 (-0.41, 1.14)	0.32 (-0.48, 1.17)	0.42 (-0.49, 1.25)	0.35 (-0.59, 1.25)	0.2 (-0.93, 1.32)	0.02 (-1.58, 1.5)	-0.14 (-2.43, 2.03)
	Other/None	0.07 (-1, 1.16)	0.27 (-0.67, 1.19)	0.41 (-0.64, 1.5)	0.79 (-0.31, 1.93)	1.21 (0.1, 2.27)	1.33 (0.14, 2.56)	1.67 (0.09, 3.26)	2.1 (0.02, 4.32)	3.42 (0.52, 6.15)
Ethnicity	Mixed	-0.56 (-1.82, 0.7)	-0.46 (-1.8, 0.83)	-0.36 (-1.8, 0.76)	-0.53 (-1.82, 0.55)	-0.84 (-2.01, 0.71)	-0.74 (-2.24, 1.3)	-0.45 (-2.43, 1.78)	-0.55 (-2.77, 2.18)	-0.3 (-3.47, 5.21)
	Indian	-1.28 (-2.3, -0.25)	-0.89 (-1.98, 0.17)	-0.7 (-1.86, 0.52)	-0.59 (-1.79, 0.52)	-0.74 (-1.89, 0.54)	-0.61 (-1.95, 0.96)	-0.41 (-1.96, 1.7)	0.1 (-2.34, 4.45)	1.69 (-2.42, 5.34)

Variable	Q10	Q20	Q30	Q40	Q50	Q60	Q70	Q80	Q90
Pakistani	-0.58 (-2.31, 0.58)	-0.46 (-1.52, 0.44)	-0.84 (-1.93, 0.08)	-1.41 (-2.47, -0.38)	-1.87 (-2.97, -0.59)	-1.84 (-3.17, -0.53)	-1.78 (-3.32, -0.23)	-2.28 (-4.18, -0.28)	-3.17 (-5.89, 0.44)
Bangladeshi	-1.23 (-3.45, 0.67)	-0.67 (-3.02, 1.13)	-0.46 (-2.54, 1.19)	-0.66 (-2.39, 1.06)	-0.92 (-2.74, 0.93)	-0.97 (-2.81, 0.98)	-1.16 (-3.3, 1.14)	-1.93 (-4.41, 0.54)	-3.44 (-6.3, -0.15)
Black African	-0.84 (-3.16, 0.6)	-0.91 (-2.18, 0.38)	-1.15 (-2.5, 0.96)	-0.87 (-2.8, 0.85)	-1.05 (-2.86, 0.42)	-1.63 (-3.16, 0.19)	-2.04 (-4.02, 0.08)	-2.89 (-4.97, -0.36)	-4.23 (-6.67, -0.65)
Black Caribbean	-1.36 (-3.54, 0.94)	-0.07 (-1.71, 1.12)	-0.18 (-1.4, 1.18)	-0.23 (-1.64, 1.15)	-0.23 (-1.74, 1.35)	-0.18 (-1.7, 1.87)	0.09 (-1.83, 2.5)	0.01 (-2.56, 4.32)	-0.89 (-4.21, 4.34)
Other	0.26 (-3.61, 1.92)	0.53 (-1.01, 1.81)	0.41 (-1, 2.57)	0.8 (-1.29, 3.09)	1 (-1.16, 3.3)	1.36 (-0.97, 3.36)	1.28 (-1.06, 3.97)	1.04 (-1.66, 4.99)	-0.11 (-3.26, 4.34)
Constant	3.03 (0.28, 5.34)	4.41 (2.4, 6.27)	5.41 (3.44, 7.29)	6.47 (4.54, 8.33)	7.38 (5.38, 9.4)	8.37 (6.11, 10.66)	9.89 (7, 12.89)	11.75 (7.8, 15.55)	13.71 (8.5, 18.4)
Observations	3,196	3,196	3,196	3,196	3,196	3,196	3,196	3,196	3,196
Imputations	28	28	28	28	28	28	28	28	28

Table S6: Quantile Regression Results. Fully adjusted model, females only (Column F, Table 2 in main paper)

	Variable	Q10	Q20	Q30	Q40	Q50	Q60	Q70	Q80	Q90
	6+ Months Unemployment	-0.49 (-1.55, 0.87)	0.31 (-1.07, 1.35)	0.49 (-0.52, 1.43)	0.46 (-0.53, 1.46)	0.58 (-0.64, 1.96)	1.19 (-0.32, 3.03)	2.11 (0.18, 3.84)	2.3 (0.33, 4.36)	2.48 (0.34, 4.59)
	IMD	0.01 (-0.01, 0.02)	0 (-0.01, 0.02)	0 (-0.01, 0.02)	0 (-0.01, 0.02)	0.01 (-0.01, 0.02)	0.01 (-0.01, 0.03)	0.01 (-0.01, 0.04)	0.02 (-0.01, 0.05)	0.04 (0, 0.09)
	Locus of Control	-0.22 (-0.48, 0.07)	-0.13 (-0.39, 0.13)	-0.08 (-0.33, 0.17)	-0.03 (-0.3, 0.22)	-0.04 (-0.33, 0.25)	-0.09 (-0.43, 0.26)	0 (-0.42, 0.4)	0.04 (-0.5, 0.54)	-0.24 (-0.87, 0.41)
	GHQ-12 @ Age 14/15	0.09 (-0.01, 0.2)	0.13 (0.03, 0.23)	0.13 (0.03, 0.22)	0.14 (0.04, 0.25)	0.18 (0.06, 0.29)	0.22 (0.09, 0.37)	0.25 (0.09, 0.42)	0.3 (0.08, 0.54)	0.45 (0.17, 0.7)
	GHQ-12 @ Age 16/17	0.1 (0.06, 0.15)	0.14 (0.09, 0.19)	0.17 (0.12, 0.21)	0.18 (0.13, 0.23)	0.2 (0.15, 0.26)	0.26 (0.19, 0.33)	0.31 (0.23, 0.38)	0.35 (0.26, 0.44)	0.36 (0.25, 0.46)
Self-Rated Health @ Age 14/15	Fairly Good	0.22 (-0.31, 0.76)	0.25 (-0.21, 0.71)	0.41 (-0.02, 0.84)	0.47 (0.01, 0.91)	0.43 (-0.07, 0.97)	0.51 (-0.06, 1.07)	0.48 (-0.16, 1.1)	0.48 (-0.36, 1.34)	0.81 (-0.33, 1.98)
	Not Very Good	0.86 (-0.9, 2.34)	0.75 (-0.68, 2.21)	1.04 (-0.5, 2.66)	1.45 (-0.27, 2.65)	1.23 (-0.19, 2.74)	1.17 (-0.45, 3.64)	1.72 (-0.52, 5.2)	2.47 (-0.77, 5.54)	2 (-0.98, 4.94)
	Not Good at All	0.08 (-2.2, 1.82)	-0.68 (-2.71, 1.58)	-0.95 (-2.75, 3.35)	-1.01 (-3.36, 4.69)	-0.3 (-4.29, 5.38)	0.58 (-4.7, 6.06)	1.08 (-4.73, 7.59)	0.98 (-4.27, 8.35)	1.8 (-5.52, 7.52)
Self-Rated Health @ Age 16/17	Fairly Good	0.2 (-0.35, 0.71)	0.21 (-0.25, 0.69)	0.14 (-0.29, 0.6)	0.23 (-0.24, 0.7)	0.21 (-0.31, 0.72)	0.06 (-0.55, 0.66)	0.11 (-0.55, 0.77)	0.26 (-0.7, 1.19)	0.27 (-0.89, 1.5)
	Not Very Good	-0.55 (-1.84, 0.4)	-0.26 (-1.45, 0.99)	0.27 (-0.78, 1.29)	0.48 (-0.53, 1.46)	0.53 (-0.58, 1.61)	0.45 (-0.75, 1.85)	0.49 (-0.84, 2.13)	0.3 (-1.42, 2.18)	0.43 (-2.22, 3.01)

	Variable	Q10	Q20	Q30	Q40	Q50	Q60	Q70	Q80	Q90
	Not Good at All	-1.04 (-3.03, 1.79)	-0.51 (-3.23, 1.92)	-0.48 (-2.67, 2.33)	0.06 (-2.55, 3.24)	0.78 (-2.6, 4.77)	1.04 (-2.6, 6.02)	1.62 (-2.64, 5.78)	1.19 (-2.77, 6.38)	1.46 (-3.71, 7.18)
Disabled	Yes, school not affected	0.17 (-0.85, 0.98)	0.19 (-0.57, 0.97)	0.12 (-0.61, 0.86)	0.07 (-0.75, 1.06)	0.46 (-0.69, 1.75)	0.95 (-0.35, 2.26)	1.25 (-0.14, 2.87)	1.48 (-0.16, 3.8)	1.79 (-0.63, 4.17)
	Yes, school affected	0.14 (-1.32, 1.33)	-0.07 (-1.2, 0.96)	-0.08 (-1.15, 0.96)	-0.07 (-1.2, 1.19)	0.26 (-1.05, 1.52)	0.38 (-0.91, 1.72)	0.32 (-1.14, 2.42)	0.79 (-1.48, 3.39)	1.72 (-1.13, 4.66)
	Risk Behaviours	-0.03 (-0.23, 0.1)	-0.14 (-0.27, 0.01)	-0.2 (-0.35, 0.04)	-0.13 (-0.37, 0.12)	-0.06 (-0.3, 0.17)	-0.03 (-0.26, 0.23)	0 (-0.26, 0.28)	0.03 (-0.33, 0.43)	-0.08 (-0.51, 0.34)
	Attitude to School	-0.04 (-0.07, 0)	-0.03 (-0.06, 0)	-0.03 (-0.07, 0)	-0.03 (-0.07, 0.01)	-0.02 (-0.06, 0.02)	-0.01 (-0.05, 0.04)	-0.01 (-0.07, 0.04)	-0.02 (-0.11, 0.05)	-0.03 (-0.12, 0.06)
	# Waves Bullied, 1-3	-0.03 (-0.25, 0.19)	0.08 (-0.12, 0.27)	0.13 (-0.08, 0.33)	0.17 (-0.03, 0.37)	0.23 (0.02, 0.44)	0.34 (0.09, 0.58)	0.46 (0.15, 0.77)	0.61 (0.2, 1.01)	0.68 (0.14, 1.21)
Qualifications	NVQ 4	0.47 (-0.3, 1.18)	0.3 (-0.27, 0.92)	0.38 (-0.2, 0.99)	0.48 (-0.11, 1.06)	0.54 (-0.09, 1.16)	0.7 (-0.03, 1.4)	0.71 (-0.2, 1.54)	0.45 (-0.79, 1.6)	0.39 (-1.11, 1.92)
	NVQ 3	0.24 (-0.57, 0.99)	0.03 (-0.57, 0.65)	-0.05 (-0.66, 0.57)	0.06 (-0.58, 0.7)	0.28 (-0.41, 0.98)	0.54 (-0.25, 1.39)	0.67 (-0.27, 1.54)	0.5 (-0.79, 1.8)	0.8 (-0.79, 2.58)
	NVQ 2	-0.18 (-1.03, 0.67)	-0.29 (-0.97, 0.4)	-0.24 (-0.86, 0.38)	-0.17 (-0.83, 0.51)	-0.03 (-0.77, 0.68)	0.05 (-0.78, 0.88)	0.1 (-0.92, 1.06)	-0.33 (-1.78, 1.05)	-0.85 (-2.51, 0.89)
	NVQ 1	-0.3 (-1.61, 0.78)	-0.47 (-1.42, 0.59)	-0.31 (-1.27, 0.69)	-0.19 (-1.21, 0.86)	-0.07 (-1.2, 1.11)	0.29 (-1.04, 1.52)	0.49 (-0.96, 2.06)	0.63 (-1.44, 2.55)	0.04 (-2.22, 2.6)

	Variable	Q10	Q20	Q30	Q40	Q50	Q60	Q70	Q80	Q90
	No/Other Qual	0.17 (-1.3, 1.42)	-0.01 (-0.95, 1.06)	-0.07 (-1.11, 1.01)	0.02 (-1.04, 1.2)	0.25 (-0.99, 1.51)	0.6 (-0.76, 2.07)	1.1 (-0.69, 3.3)	1.34 (-1.13, 3.68)	0.45 (-2.1, 3.1)
Parental NS-SEC	Intermediate	0.5 (-0.16, 1.21)	0.45 (-0.13, 1.01)	0.46 (-0.08, 0.97)	0.37 (-0.19, 0.94)	0.3 (-0.32, 0.91)	0.09 (-0.66, 0.82)	-0.14 (-0.93, 0.7)	-0.14 (-1.17, 0.98)	0.05 (-1.52, 1.58)
	Routine	0.62 (0.02, 1.29)	0.72 (0.18, 1.26)	0.81 (0.24, 1.35)	0.8 (0.22, 1.37)	0.81 (0.15, 1.46)	0.83 (0.02, 1.62)	0.81 (-0.03, 1.67)	1.17 (0.07, 2.3)	1.44 (-0.09, 2.94)
	LTU	-0.33 (-3, 1.53)	0.08 (-1.51, 1.42)	0.1 (-1.27, 1.45)	0.19 (-1.23, 1.89)	0.58 (-1.19, 2.49)	1.07 (-0.95, 3.32)	1.81 (-0.34, 4.17)	2.21 (-0.37, 5)	2.23 (-0.75, 5.23)
Parental Education	Other HE	-0.04 (-0.83, 0.67)	-0.11 (-0.79, 0.55)	-0.15 (-0.84, 0.58)	-0.01 (-0.78, 0.75)	-0.01 (-0.83, 0.8)	0 (-0.91, 0.89)	0.08 (-0.95, 1.07)	0.14 (-1.15, 1.44)	0.11 (-1.59, 1.76)
	A-Level	-0.39 (-1.24, 0.34)	-0.58 (-1.26, 0.09)	-0.73 (-1.38, -0.04)	-0.71 (-1.45, -0.01)	-0.86 (-1.64, -0.06)	-0.89 (-1.8, 0.01)	-1.03 (-2.03, -0.04)	-1.36 (-2.59, -0.15)	-1.76 (-3.53, 0.09)
	GCSE A-C	-0.42 (-1.24, 0.32)	-0.49 (-1.17, 0.17)	-0.5 (-1.15, 0.14)	-0.54 (-1.21, 0.14)	-0.68 (-1.45, 0.15)	-0.55 (-1.5, 0.44)	-0.43 (-1.42, 0.56)	-0.45 (-1.72, 0.89)	-0.5 (-2.3, 1.26)
	Other/None	-0.3 (-1.31, 0.6)	-0.25 (-1.07, 0.54)	-0.38 (-1.17, 0.43)	-0.36 (-1.22, 0.48)	-0.44 (-1.38, 0.57)	-0.46 (-1.6, 0.71)	-0.69 (-1.97, 0.6)	-0.89 (-2.55, 0.75)	-0.88 (-2.96, 1.34)
Ethnicity	Mixed	-0.78 (-2.17, 0.52)	-0.66 (-1.82, 0.36)	-0.73 (-1.79, 0.24)	-0.95 (-2, 0.24)	-0.99 (-2.19, 0.5)	-0.74 (-2.3, 1.28)	0.17 (-2.15, 3.6)	1.61 (-1.3, 5.99)	3.46 (-0.87, 8.99)
	Indian	-1.22 (-2.47, -0.06)	-0.93 (-1.86, -0.2)	-1.01 (-1.78, -0.1)	-0.81 (-1.77, 0.12)	-0.59 (-1.59, 0.36)	-0.51 (-1.47, 0.53)	-0.27 (-1.5, 1.3)	0.16 (-1.5, 1.83)	-0.45 (-2.45, 1.82)

Variable	Q10	Q20	Q30	Q40	Q50	Q60	Q70	Q80	Q90
Pakistani	-0.75 (-1.99, 0.44)	-0.56 (-1.81, 0.44)	-0.4 (-1.4, 0.51)	-0.44 (-1.46, 0.57)	-0.52 (-1.61, 0.72)	-0.34 (-1.64, 1.03)	0.02 (-1.49, 1.67)	0 (-1.9, 2.07)	-0.34 (-2.96, 2.46)
Bangladeshi	-2.56 (-4.15, -0.34)	-1.72 (-3.95, -0.21)	-1.19 (-2.63, -0.17)	-1.22 (-2.39, 0.04)	-1.24 (-2.64, 0.21)	-1.26 (-2.79, 0.1)	-1.52 (-2.97, 0.22)	-1.85 (-3.99, 0.38)	-2.53 (-5.46, 1.31)
Black African	-0.24 (-1.27, 1)	-0.03 (-1.21, 0.93)	-0.3 (-1.24, 0.88)	-0.34 (-1.57, 1.48)	0.02 (-1.73, 2.05)	0.36 (-1.5, 2.26)	0.3 (-1.52, 2.51)	0.26 (-2.02, 3.37)	0.56 (-2.79, 4.85)
Black Caribbean	-2.4 (-3.58, -1.02)	-1.96 (-3.07, -1.04)	-1.93 (-2.96, -0.87)	-1.8 (-2.95, -0.46)	-1.53 (-3, -0.18)	-1.33 (-2.78, 0.05)	-1.29 (-2.71, 0.2)	-1.53 (-3.32, 0.32)	-2.37 (-4.63, 0.3)
Other	-1.31 (-3.64, 0.12)	-1.41 (-2.77, 0.23)	-0.98 (-2.67, 0.71)	-0.52 (-2.37, 1.32)	-0.02 (-2.08, 1.5)	0.13 (-1.69, 2.43)	0.82 (-1.37, 2.94)	0.76 (-1.56, 3.49)	-0.04 (-2.82, 2.4)
Constant	5.87 (4.36, 7.39)	6.63 (5.1, 8.15)	7.57 (6.12, 8.95)	7.89 (6.38, 9.59)	8.21 (6.23, 10.11)	7.98 (5.92, 9.97)	8.58 (6.26, 11)	10.06 (6.65, 13.69)	12.55 (8.41, 16.47)
Observations	4,167	4,167	4,167	4,167	4,167	4,167	4,167	4,167	4,167
Imputations	28	28	28	28	28	28	28	28	28

References

Buddelmeyer, H., & Powdthavee, N. (2016). Can having internal locus of control insure against negative shocks? Psychological evidence from panel data. *Journal of Economic Behavior & Organization*, *122*, 88–109. https://doi.org/10.1016/j.jebo.2015.11.014

Caliendo, M., Cobb-Clark, D. A., & Uhlendorff, A. (2015). Locus of control and job search strategies. *Review of Economics and Statistics*, *97*(1), 88–103. https://doi.org/10.1162/REST_a_00459

Chowdry, H., Crawford, C., & Goodman, A. (2011). The role of attitudes and behaviours in explaining socio-economic differences in attainment at age 16. *Longitudinal and Life Course Studies*, *2*(1). https://doi.org/10.14301/llcs.v2i1.141

Cobb-Clark, D. A. (2015). Locus of control and the labor market. *IZA Journal of Labor Economics*, *4*(1), 3. https://doi.org/10.1186/s40172-014-0017-x

Cobb-Clark, D. A., Kassenboehmer, S. C., & Schurer, S. (2014). Healthy habits: The connection between diet, exercise, and locus of control. *Journal of Economic Behavior & Organization*, *98*, 1–28. https://doi.org/10.1016/j.jebo.2013.10.011

Cobb-Clark, D. A., & Schurer, S. (2013). Two economists' musings on the stability of locus of control. *Economic Journal*, *123*(570). https://doi.org/10.1111/ecoj.12069

Crawford, C., Dearden, L., & Greaves, E. (2011). *Does when you are born matter?The impact ofmonthof birth onchildren's cognitive and non-cognitive skills in England*. Institue for Fiscal Studies. https://www.ifs.org.uk/bns/bn122.pdf

Department for Education, Crawford, C., & Cribb, J. (2012). *Gap year takers: Uptake, trends and long term outcomes*. Department for Education. https://assets.publishing.service.gov.uk/government/uploads/system/uploads/attachment_data/file/219637/DFE-RR252.pdf https://www.gov.uk/government/publications/gap-year-takers-uptake-trends-and-long-term-outcomes

Gladwell, D., Popli, G., & Tsuchiya, A. (2016). *A Dynamic Analysis of Skill Formation and NEET status*. https://cemapre.iseg.ulisboa.pt/educonf/4e3/files/Papers/Popli.pdf

Hankins, M. (2008). The factor structure of the twelve item General Health Questionnaire (GHQ-12): The result of negative phrasing? *Clinical Practice and Epidemiology in Mental Health*, *4*(1), 10. https://doi.org/10.1186/1745-0179-4-10

For peer review only

Mendolia, S., & Walker, I. (2014a). THE EFFECT OF NONCOGNITIVE TRAITS ON HEALTH BEHAVIOURS IN ADOLESCENCE: THE EFFECT OF NONCOGNITIVE TRAITS ON HEALTH BEHAVIOURS IN ADOLESCENCE. *Health Economics*, *23*(9), 1146–1158. https://doi.org/10.1002/hec.3043

Mendolia, S., & Walker, I. (2014b). The effect of personality traits on subject choice and performance in high school: Evidence from an English cohort. *Economics of Education Review*, *43*, 47–65. https://doi.org/10.1016/j.econedurev.2014.09.004

Mendolia, S., & Walker, I. (2015). Youth unemployment and personality traits. *IZA Journal of Labor Economics*, *4*(1). https://doi.org/10.1186/s40172-015-0035-3

Ng-Knight, T., & Schoon, I. (2017). Can Locus of Control Compensate for Socioeconomic Adversity in the Transition from School to Work? *Journal of Youth and Adolescence*, *46*(10), 2114–2128. https://doi.org/10.1007/s10964-017-0720-6

Piatek, R., & Pinger, P. (2016). Maintaining (Locus of) Control? Data Combination for the Identification and Inference of Factor Structure Models. *Journal of Applied Econometrics*, *31*(4), 734–755. https://doi.org/10.1002/jae.2456

Rotter, J. B. (1966). Generalized expectancies for internal versus external control of reinforcement. *Psychological Monographs*, *80*(1), 1–28. https://doi.org/10.1037/h0092976

Rotter, J. B. (1975). Some problems and misconceptions related to the construct of internal versus external control of reinforcement. *Journal of Consulting and Clinical Psychology*, *43*(1), 56–67. https://doi.org/10.1037/h0076301

Wijedasa, D. (2017). 'People like me don't have much of a chance in life': Comparing the locus of control of young people in foster care with that of adoptees, children from disadvantaged backgrounds and children in the general population. *Adoption & Fostering*, *41*(1), 5–19. https://doi.org/10.1177/0308575916684299

For peer review only

STROBE 2007 (v4) Statement—Checklist of items that should be included in reports of *cohort studies*

Section/Topic	Item #	Recommendation	Reported on page #
**Title and abstract**	1	(*a*) Indicate the study's design with a commonly used term in the title or the abstract	1
		(*b*) Provide in the abstract an informative and balanced summary of what was done and what was found	2
**Introduction**			
Background/rationale	2	Explain the scientific background and rationale for the investigation being reported	3
Objectives	3	State specific objectives, including any prespecified hypotheses	4
**Methods**			
Study design	4	Present key elements of study design early in the paper	4
Setting	5	Describe the setting, locations, and relevant dates, including periods of recruitment, exposure, follow-up, and data collection	4
Participants	6	(*a*) Give the eligibility criteria, and the sources and methods of selection of participants. Describe methods of follow-up	4
		(*b*) For matched studies, give matching criteria and number of exposed and unexposed	
Variables	7	Clearly define all outcomes, exposures, predictors, potential confounders, and effect modifiers. Give diagnostic criteria, if applicable	4-5
Data sources/ measurement	8*	For each variable of interest, give sources of data and details of methods of assessment (measurement). Describe comparability of assessment methods if there is more than one group	*4-5*
Bias	9	Describe any efforts to address potential sources of bias	6
Study size	10	Explain how the study size was arrived at	4
Quantitative variables	11	Explain how quantitative variables were handled in the analyses. If applicable, describe which groupings were chosen and why	4-5
Statistical methods	12	(*a*) Describe all statistical methods, including those used to control for confounding	6
		(*b*) Describe any methods used to examine subgroups and interactions	6
		(*c*) Explain how missing data were addressed	6
		(*d*) If applicable, explain how loss to follow-up was addressed	6
		(*e*) Describe any sensitivity analyses	6
**Results**			

For peer review only

Participants	13*	(a) Report numbers of individuals at each stage of study—eg numbers potentially eligible, examined for eligibility, confirmed eligible, included in the study, completing follow-up, and analysed	4
		(b) Give reasons for non-participation at each stage	
		(c) Consider use of a flow diagram	
Descriptive data	14*	(a) Give characteristics of study participants (eg demographic, clinical, social) and information on exposures and potential confounders	6-8
		(b) Indicate number of participants with missing data for each variable of interest	6-8
		(c) Summarise follow-up time (eg, average and total amount)	4
Outcome data	15*	Report numbers of outcome events or summary measures over time	6
Main results	16	(*a*) Give unadjusted estimates and, if applicable, confounder-adjusted estimates and their precision (eg, 95% confidence interval). Make clear which confounders were adjusted for and why they were included	9
		(*b*) Report category boundaries when continuous variables were categorized	
		(*c*) If relevant, consider translating estimates of relative risk into absolute risk for a meaningful time period	
Other analyses	17	Report other analyses done—eg analyses of subgroups and interactions, and sensitivity analyses	10
**Discussion**			
Key results	18	Summarise key results with reference to study objectives	10
**Limitations**			
Interpretation	20	Give a cautious overall interpretation of results considering objectives, limitations, multiplicity of analyses, results from similar studies, and other relevant evidence	11
Generalisability	21	Discuss the generalisability (external validity) of the study results	11
**Other information**			
Funding	22	Give the source of funding and the role of the funders for the present study and, if applicable, for the original study on which the present article is based	12

*Give information separately for cases and controls in case-control studies and, if applicable, for exposed and unexposed groups in cohort and cross-sectional studies.

Note: An Explanation and Elaboration article discusses each checklist item and gives methodological background and published examples of transparent reporting. The STROBE checklist is best used in conjunction with this article (freely available on the Web sites of PLoS Medicine at http://www.plosmedicine.org/, Annals of Internal Medicine at http://www.annals.org/, and Epidemiology at http://www.epidem.com/). Information on the STROBE Initiative is available at www.strobe-statement.org.

For peer review only
